# Synthesis of novel mono- and bis-pyrazolylthiazole derivatives as anti-liver cancer agents through EGFR/HER2 target inhibition

**DOI:** 10.1186/s13065-023-00921-6

**Published:** 2023-06-08

**Authors:** Mostafa E. Salem, Esraa M. Mahrous, Eman A. Ragab, Mohamed S. Nafie, Kamal M. Dawood

**Affiliations:** 1 Department of Chemistry, Faculty of Science, Cairo University, Giza, 12613 Egypt; 2 Department of Chemistry (Biochemistry program), Faculty of Science, Suez Canal University, Ismailia, 41522 Egypt

**Keywords:** EGFR/HER2, Liver cancer, Thiosemicarbazides bis-pyrazolylthiazoles, Naphthalenes

## Abstract

3-Bromoacetyl-4-(2-naphthoyl)-1-phenyl-1H-pyrazole (6) was synthesized from 2-acetylnaphthalene and was used as a new key building block for constructing the title targets. Thus, the reaction of 6 with the thiosemicarbazones 7a–d and 9–11 afforded the corresponding simple naphthoyl-(3-pyrazolyl)thiazole hybrids 8a–d and 12 ~ 14. The symmetric bis-(2-naphthoyl-pyrazol-3-yl)thiazol-2-yl)hydrazono)methyl)phenoxy)alkanes 18a-c and 21a-c were similarly synthesized from reaction of 6 with the appropriate bis-thiosemicarbazones 17a-c and 19a-c, respectively. The synthesized two series of simple and symmetrical bis-molecular hybrid merging naphthalene, thiazole, and pyrazole were evaluated for their cytotoxicity. Compounds 18b,c and 21a showed the most potent cytotoxicity (IC_50_ = 0.97–3.57 µM) compared to Lapatinib (IC_50_ = 7.45 µM). Additionally, they were safe (non-cytotoxic) against the THLE2 cells with higher IC_50_ values. Compounds 18c exhibited promising EGFR and HER-2 inhibitory activities with IC_50_ = 4.98 and 9.85 nM, respectively, compared to Lapatinib (IC_50_ = 6.1 and 17.2 nM). Apoptosis investigation revealed that 18c significantly activated apoptotic cell death in HepG2 cells, increasing the death rate by 63.6-fold and arresting cell proliferation at the S-phase. Compound 18c upregulated P53 by 8.6-fold, Bax by 8.9-fold, caspase-3,8,9 by 9, 2.3, and 7.6-fold, while it inhibited the Bcl-2 expression by 0.34-fold. Thereby, compound 18c exhibited promising cytotoxicity against EGFR/HER2 inhibition against liver cancer.

**Supplementary Information:**

The online version contains supplementary material available at 10.1186/s13065-023-00921-6.

## Introduction

**Fig. 1:**
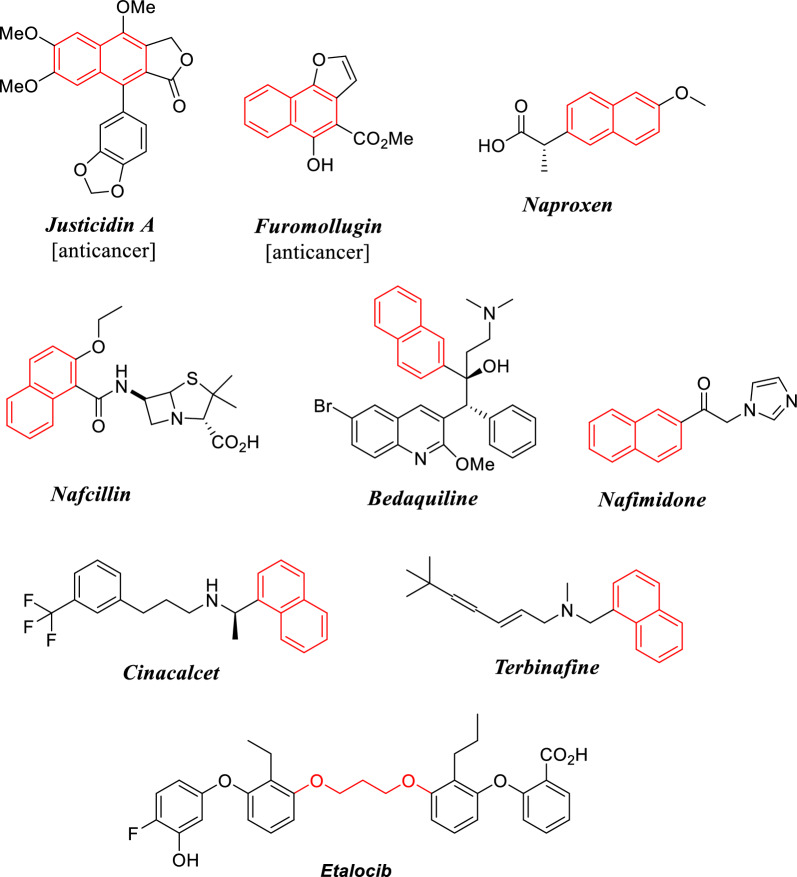
Examples of naphthalene- and (bis-aryl)propylenedioxy-based marketed Drugs

**Fig. 2:**
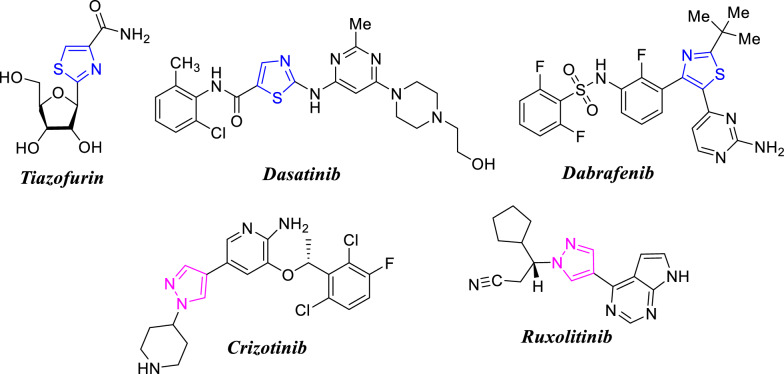
Examples of thiazole- and pyrazole-based anti-cancer drugs

Naphthalene ring is a great part of several significant biologically active synthetic and naturally-based organic compounds. It constitutes a distinguished core of various promising anti-cancer agents [1–7]. Some naturally occurring naphthalene-based compounds, such as *Justicidin A*, and *Furomollugin* (Fig. 1), revealed outstanding anti-cancer activities [8, 9]. In addition, the naphthalene fragment was found in some commercially available marketed drugs such as *Naproxen* (anti-inflammatory drug), *Cinacalcet* (parathyroid carcinoma drug), *Terbinafine* (antifungal), *Nafcillin* (antibiotic), *Bedaquiline* (antitubercular), and *Nafimidone* (anticonvulsant) as shown in Fig. 1 [10, 11]. Interestingly, *Etalocib*, having 1,3-bis-phenoxypropane scaffold, was a drug candidate under phase trial III for the treatment of various types of cancer (Fig. 1) [12, 13]. Furthermore, many reviews reported the importance of thiazole-based heterocyclic compounds as essential cores in several medicinally important compounds [14–18]. Thiazole nucleus is a fundamental part of some clinically applied anticancer drugs, such as *Tiazofurin* (1) [19], *Dasatinib* (2) [20], and *Dabrafenib* (3) [21] as depicted in Fig. 2.

In signal transduction pathways that control cell proliferation and differentiation, phosphotyrosine kinase, also known as receptor protein tyrosine kinase (RPTK), plays a crucial role. Transmembrane protein tyrosine kinase (PTK) epidermal growth factor receptor (EGFR) is a key regulator of cell proliferation, differentiation, and migration through ligand-induced dimerization [22]. The epidermal growth factor receptor (EGFR) tyrosine kinase-mediated cell growth signaling pathway is involved in the initiation and progression of a wide variety of solid tumors, including those of the head and neck, lung, breast, bladder, prostate, and kidney [23]. As a result, EGFR tyrosine kinase is a promising therapeutic target. Over-expression or aberrant activation of EGFR and HER-2 is a major cause of cell malignant transformation, making them two of the most actively studied targets in oncology today [24]. The potentially useful new therapeutic anti-cancer drugs that block EGFR and/or HER-2 kinase activity upon ATP attachment to the receptor. For individuals with non-small-cell lung cancer, the US Food and Drug Administration (FDA) approved the inhibitors Gefitinib (Iressa) and Erlotinib (Tarceva) [25]. A synergistic anti-cancer effect may be displayed by the pyrazole ring in conjunction with the thiazole and naphthalene rings in the combined substructures [26].

Besides, pyrazole moiety is one of the most predominant classes of nitrogen heterocycles that are widely found in a huge number of synthetic and naturally occurring organic compounds that possess significant anti-cancer activity [27–29]. Interestingly, some pyrazole scaffolds were also approved by FDA as commercially available anti-cancer drugs, such as *Crizotinib* [for treatment of non-small cell lung carcinoma (NSCLC)] [30, 31], and *Ruxolitinib* (for treatment of myelofibrosis) [32] (Fig. 2).

**Fig. 3:**
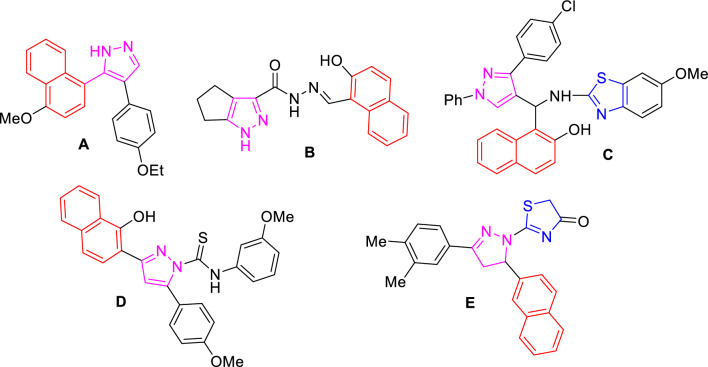
Anti-cancer molecular hybrids **A–E** have pyrazole, thiazole, and naphthalene moieties

Merging two or more aryl(heteroaryl) fragments in one molecule to construct a new hybrid molecule is a beneficial tool for the designing of effective therapeutic agents [33, 34]. In this regard, some naphthyl-pyrazole hybrids **A ~ E** demonstrated potent in vitro anti-cancer efficiency against human breast cancer cell line MCF-7 (Fig. 3) [35–38]. Interestingly, some of these derivatives exhibited high anti-cancer activity against MCF-7 with a fivefold more active than the reference drug cisplatin [37]. In addition, some molecules involving the three scaffolds; pyrazole, thiazole, and naphthalene, were reported to have notable anti-cancer activity targeting EGFR (Fig. 3) [39, 40].

**Fig. 4:**
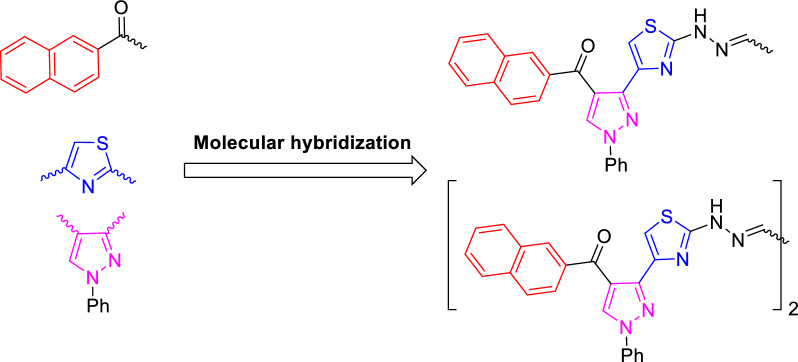
Rationale Design for molecular hybridization towards new anti-cancer agents

Recently, our research was directed towards synthesis of variant simple and bis-heterocyclic hybrids having significant anti-cancer potency against several human cancer cell lines [41–51]. Inspired by the observations mentioned above, herein we designed and synthesized a new series of molecular hybrids merging the three fragments: naphthalene, thiazole, and pyrazole, in addition to benzothiazole, benzofuran or coumarin in a simple- and symmetrical bis-molecularly hybrid forms aiming at the production of more efficient anti-cancer hybrid structures (Fig. 4).

## Results and discussion

### Chemistry

**Scheme 1:**
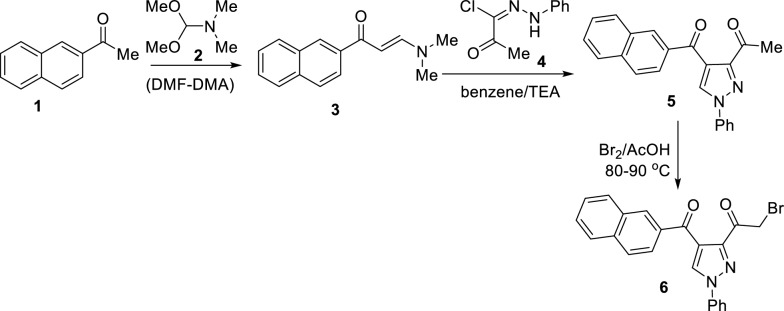
Synthesis of 3-bromoacetyl-4-(2-naphthoyl)-1-phenyl-1H-pyrazole **(6)**

A sequence of processes, as described in Scheme 1, was used to produce the desired key building block; 3-bromoacetyl-4-(2-naphthoyl)-1-phenyl-1H-pyrazole **(6)**. Thus, 2-acetylnaphthalene **(1)** was refluxed with dimethylformamide-dimethylacetal (DMF-DMA) to give 3-(dimethylamino)-1-(naphthalen-2-yl)prop-2-en-1-one **(3)**. Treatment of the latter enaminone **3** with 2-oxo-*N'*-phenylpropanehydrazonoyl chloride (**4**) in refluxing benzene in the presence of triethylamine produced 3-acetyl-4-(2-naphthoyl)-1-phenyl-1*H*-pyrazole (**5**) in a good yield. Bromination of the latter 3-acetylpyrazole **5** with bromine in acetic acid at 80–90 °C furnished the corresponding new 3-bromoacetylpyrazole derivative **6** in 78% yield (Scheme 1). The structures of the *hitherto* unreported naphthoylpyrazoles **5** and **6** were inferred from their elemental and spectral analyses, as described in the experimental section.

**Scheme 2:**
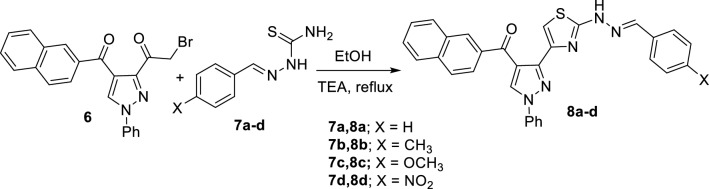
Synthesis of 2-(arylidenehydrazinyl)-4-(3-pyrazolyl)thiazole derivatives **8a–d**

The reaction of 3-bromoacetylpyrazole derivative **6** with the appropriate aldehyde-thiosemicarbazone derivatives **7a–d** in refluxing ethanol in the presence of few drops of triethylamine afforded the corresponding new 2-(benzylidenehydrazinyl)-4-(pyrazol-3-yl)thiazole derivatives **8a–d** (Scheme 2). The structures of the obtained products were substantiated from their elemental and spectral analyses. The ^1^H-NMR of compound **8a**, as a demonstrative example, showed five singlet signals at 7.95, 8.06, 8.51, 9.02 and 12.09 due to the thiazole-5-CH, CH = N, naphthyl-1-CH, pyrazole-5-CH, and NH protons, respectively. The ^13^C-NMR of **8a** revealed 25 signals due to 25 different sp^2^ carbons. Its IR showed two distinctive absorption peaks at 3437 and 1647 due to NH and C = O functions. In addition, its mass spectrometry exhibited a peak at *m/z* 499 due to its molecular ion.

**Scheme 3:**
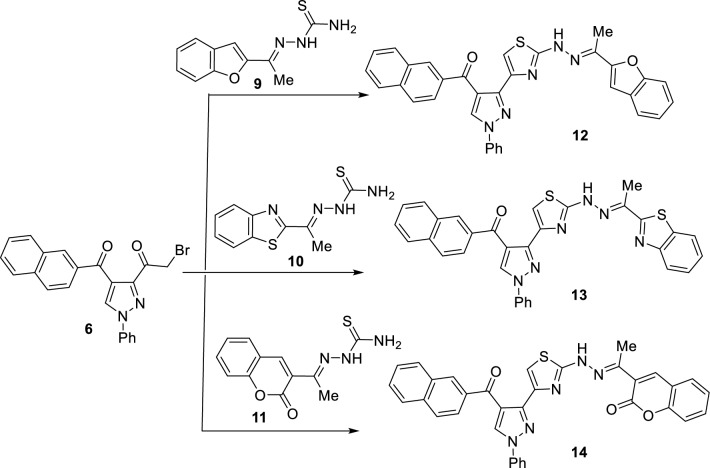
Synthesis of arylidenehydrazinyl-(1*H*-pyrazol-3-yl)thiazole derivatives **12–14**

Similarly, reaction of the 3-bromoacetylpyrazole derivative **6** with three different heterocyclyl-thiosemicarbazones **9**, **10**, and **11**, under similar reaction conditions as mentioned above, yielded the corresponding 4-(3-pyrazolyl)thiazole derivatives **12 ~ 14** having benzofuran, benzothiazole, and coumarin moieties, in 85%, 87%, and 73% yields, respectively (Scheme 3). Compounds **12–14** represent fascinating hybrid compounds that each has a different bioactive heterocyclic moiety. The structures of compounds **12–14** were established from their elemental analyses and spectral data. The ^1^H-NMR spectrum of compound **12** revealed six singlet signals at 2.31, 7.22, 7.89, 8.55, 9.04, and 11.59 due to the methyl, benzofuran-3-H, thiazole-5-CH, naphthyl-1-CH, pyrazole-5-CH, and NH protons, respectively. Its IR showed two distinctive absorption peaks at 3433 and 1648 due to NH and C = O functions. In addition, its mass spectrometry exhibited a peak at *m/z* 533 due to the molecular ion.

**Scheme 4:**
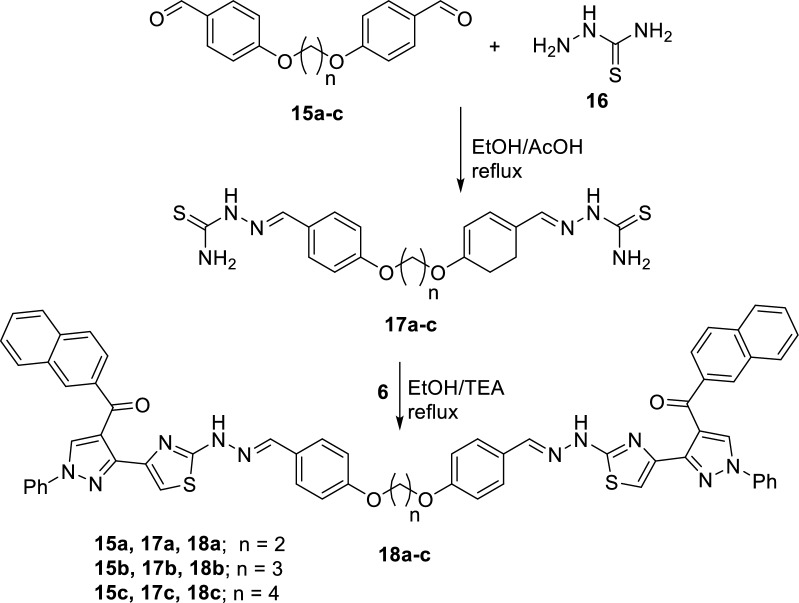
Reaction of bis(thiosemicarbazones) **17a–c** with bromoacetylpyrazole **6**

**Scheme 5:**
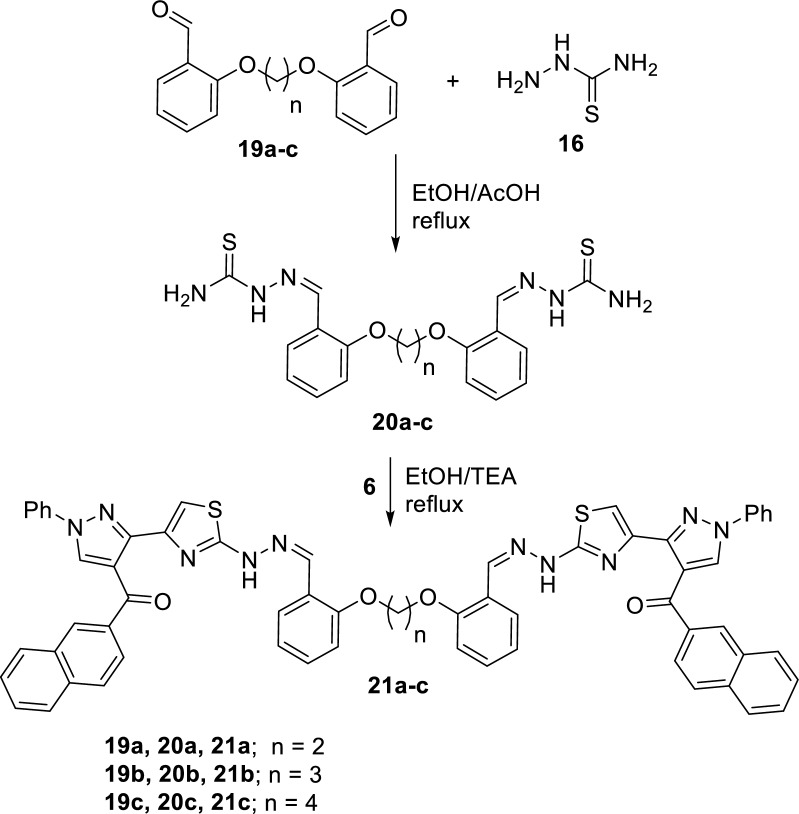
Reaction of bis(thiosemicarbazones) **20a–c** with bromoacetylpyrazole **6**

Next, we intended to construct symmetric *bis*-heterocyclic systems to compare their behaviors with the mono-heterocyclic systems that were described in the above text. Thus, two series of *bis*- pyrazolylthiazoles linked via alkyleneoxy-phenylene spacers at either *ortho-* or *para-*positions were developed. Firstly, the starting building blocks **17a-c** and **20a–c** were prepared according to methodologies reported in the literature via reaction of the appropriate *bis*(aldehydes) **15a-c** and **19a–c** with thiosemicarbazid **16** in refluxing ethanol containing few drops of acetic acid (Schemes 4 and 5). Then, the synthesis of 1,2-bis(4-((2-(4-((2-naphthoyl)-1-phenyl-1H-pyrazol-3-yl)thiazol-2-yl)hydrazono)methyl)phenoxy)ethane (**18a**) was achieved by the reaction of 3-(bromoacetyl)-4-(2-naphthoyl)-1-phenyl-1*H*-pyrazole (**6**) with 4,4'-(ethane-1,2-diylbis(oxy))dibenzaldehyde-thiosemicarbazone (**17a**) in ethanol, in the presence of few drops of triethylamine, at reflux temperature. The *bis*-pyrazolylthiazole derivative **18a** was isolated in a 79% yield (Scheme 4). Further examples; the symmetric bis(4-((2-(4-((2-naphthoyl)-1-phenyl-1H-pyrazol-3-yl)thiazol-2-yl)hydrazono)methyl)phenoxy)alkanes **18b,c** were similarly synthesized under typical reaction condition from the reaction of the bromoacetylpyrazole **6** with the appropriate 4,4'-(alkane-diylbis(oxy))dibenzaldehydethiosemicarbazone derivatives **17b,c.** The symmetric *bis*-pyrazolylthiazoles **18b,c** were obtained in 80 and 83% yields, respectively (Scheme 4). The ^1^H-NMR spectrum of compound **18a** displayed six singlet signals at 4.35, 7.91, 8.06, 8.51, 9.01 and 11.90 due to the CH_2_O, thiazole-5-CH, CH = N, naphthyl-1-CH, pyrazole-5-CH, and NH protons, respectively. The ^13^C-NMR spectrum of compound **18a** exhibited a signal at 65.4 due to an sp^3^ CH_2_O carbon in addition to 26 signals due to 26 different sp^2^ carbons The IR spectrum of compound **18a** showed two absorption bands at 3407 and 1645 cm^−1^ due to NH and C = O functions (Additional file 1).

Similarly, the 2,2'-(alkane-diylbis(oxy))dibenzaldehydethiosemicarbazone derivatives **19a-c** reacted with the bromoacetylpyrazole **6,** under typical reaction conditions as above to give the corresponding bis(2-((2-(4-((2-naphthoyl)-1-phenyl-1H-pyrazol-3-yl)thiazol-2-yl)hydrazono)-methyl)phenoxy)alkanes **21a-c** (*ortho* isomers) in 70–78% yields, as shown in Scheme 5. The structures of the pure products were established from their respective elemental and spectral analyses, as described in the experimental section. For instance, the ^1^H NMR spectrum of **21a** showed a D_2_O-exchangeable signal at 11.95 ppm assigned to NH protons as well as a sharp singlet signal at δ 8.29 due to a methine proton (N = CH). Moreover, it also featured a singlet signal at δ 4.40 due to methylene ether linkage OCH_2_.

### Biology

#### Cytotoxicity

**Fig. 5:**
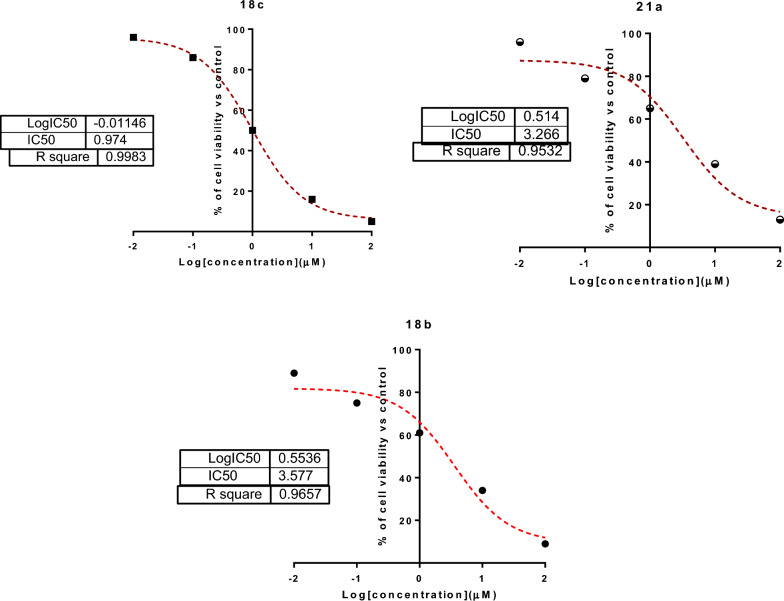
The current most potent anti-cancer mono- and bis-pharmacophores

**Fig. 6:**
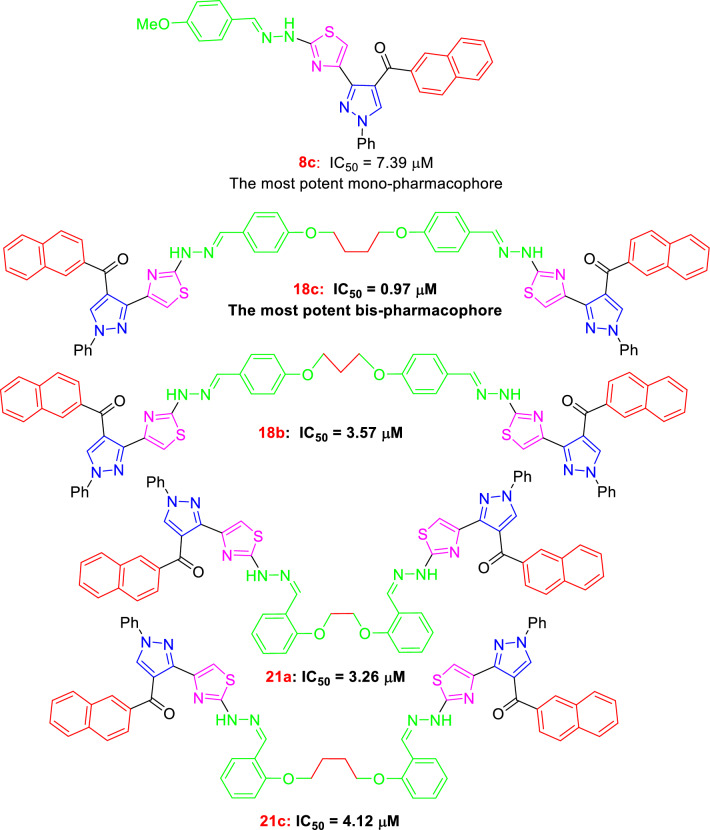
Dose–response nonlinear regression curve fitting the percentage of cell viability *vs*. log [conc. µM], R square ≈ 1 using the GraphPad prism

The synthesized compounds were screened for their cytotoxicity against HepG2 cells using the MTT assay. As seen in Table 1, the bis-pharmacophores exhibited much higher cytotoxicity than the mono-pharmacophoric compounds. Fourteen substrates were tested for their anti-cancer activity. Among them, eight compounds (**8c, 12, 13, 14, 18b,c, 21a,c**) were found to have high anti-cancer potency with IC_50_ values ranging between 0.97 ~ 7.39 µM much better than the reference drug Lapatinib (IC_50_ = 7.45 µM). The order of anti-cancer potency of the tested compounds was arranged in the following order: **18c > 21a > 18b > 21c > 14 > 13 > 12 > 8c.** Interestingly, compounds **18c**, **21a**, and **18b** showed potent cytotoxicity with IC_50_ values of 0.97, 3.26 and 3.57 µM, respectively, compared with the reference anti-cancer drug Lapatinib (IC_50_ = 7.45 µM). These compounds caused cell viability at the highest concentration [100 µM] by 5%, 13%, and 9%, respectively, as shown in Fig. 5. Additionally, they were safe (non-cytotoxic) against the THLE2 cells with higher IC_50_ values. Among the mono-pharmacophoric compounds **8a-d**, the best activity was recorded for the benzaldehyde-hydrazone derivative **8c** having para-methoxy group. For comparison reasons, the activity of the bis-pyrazolylthiazole pharmacophores connected via para-alkanedioxy or ortho-alkanedioxy linkers **18a-c** and **21a-c** were compared with the simple pyrazolylthiazole having para-methoxybenzaldehyde hydrazine **8c**. Interestingly, doubling the pharmacophoric scaffold led to about eightfold enhancement in the anti-cancer potency (IC_50_ = 0.97 µM for **18c** vs. 7.39 µM for **8c**), as presented in Fig. 6. In addition, the mono-pyrazolylthiazole derivatives having acetyl-heterocyclic-hydrazones **12 ~ 14** (benzofuranyl, benzothiazolyl and coumarinyl) were more potent against HepG2 cell line than those having aryl moieties **8a-d**.

**Table 1:** Cytotoxic IC_50_ values of the tested compounds against HepG2 and THLE2 cell lines using the MTT assay

Compounds	IC_50_ ± SD^a^ (µM)	
	HepG2	THLE2
6	16.3 ± 0.29	34.5 ± 1.26
8a	8.64 ± 0.29	46.2 ± 2.01
8b	8.16 ± 0.34	41.6 ± 1.86
8c	7.39 ± 0.29	≥ 50
8d	11.3 ± 0.34	≥ 50
12	5.48 ± 0.66	≥ 50
13	5.16 ± 0.29	≥ 50
14	4.36 ± 0.38	39.5 ± 1.74
18a	8.34 ± 0.48	39.8 ± 1.59
18b	3.57 ± 0.67	≥ 50
18c	0.97 ± 0.09	≥ 50
21a	3.26 ± 0.14	≥ 50
21b	9.36 ± 0.47	49.4 ± 2.1
21c	4.12 ± 0.19	45.6 ± 1.87
Lapatinib	7.45 ± 0.32	≥ 50

^a^Values are expressed as Mean ± SD of three independent triplets (n = 3)

#### EGFR and HER2 kinase inhibitory assay

Compounds **18c, 21a**, and **18b** with the highest cytotoxic activity against HepG2 cells were tested against the EGFR/HER2 inhibitory activities to highlight their mechanistic study. As seen in Table 2, the tested compounds exhibited promising dual EGFR/HER2 inhibition activities. Interestingly, compound **18c** had IC_50_ values of 4.98 and 9.85 nM, respectively compared to Lapatinib (IC_50_ = 6.1 and 17.2 nM). Additionally, compounds **21a** and **18b** exhibited promising EGFR/HER2 inhibition with IC_50_ values of 5.8, 7.04 nM against EGFR and 14.2, 16.3 nM against HER2. Hence, compound **18c** was further investigated for apoptotic cell death in HepG2 cells. These findings agreed with previous studies [52–54] about hybrid structures having pyrazole and thiazole rings, revealing that the two combined moieties might exhibit synergistic anti-cancer effects as potential EGFR and HER-2 inhibitory agents.

**Table 2:** IC_50_ values of EGFR and HER2 kinase activities of the most potent compounds

Compound	IC_50_ [nM]^a^	
	EGFR kinase	HER2 kinase
18b	7.04	16.3
18c	4.98	9.85
21a	5.8	14.2
Lapatinib	6.1	17.2

^a^Values are expressed as an average of three independent replicates. IC_50_ values were calculated using a sigmoidal non-linear regression curve fit of percentage inhibition against five concentrations of each compound

#### Apoptotic investigation

##### Annexin V/PI staining with cell cycle analysis

**Fig. 7:**
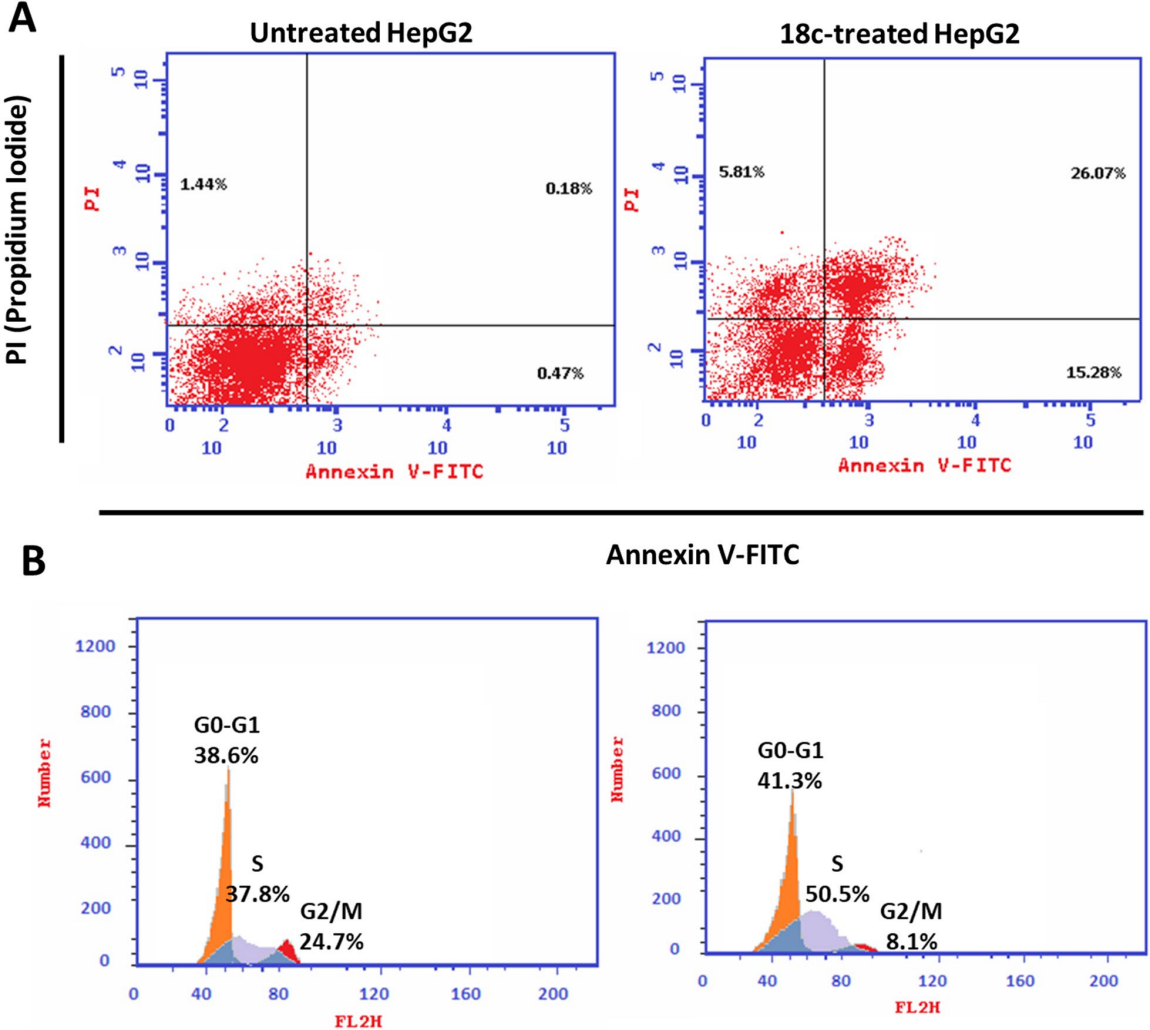
**A** Cryptographs of annexin-V/Propidium Iodide staining of untreated and **18c**-treated HepG2 cells with the IC_50_ values, 48 h, “Q1-UL (necrosis, AV-/PI +), Q1-UR (late apoptotic cells, AV + /PI +), Q1-LL (normal cells, AV-/PI-), Q1-LR (early apoptotic cells, AV + /PI-)”, **B** Percentage of cell population at each cell cycle “G0-G1, S, G2/M” using flow cytometry

The apoptotic activity of compound **18c** (IC_50_ = 0.97 M, 48 h) was evaluated by comparing the percentage of dead cells in untreated and treated HepG2 cells using flow cytometry after staining with Annexin V and PI. As shown in Fig. 7A, compound **18c** significantly activated apoptotic cell death in HepG2 cells, increasing the death rate by 63.6-fold; it induced total apoptosis by 41.35% (15.28% for early apoptosis, 26.07% for late apoptosis) compared to the untreated control group (0.65%).

Afterward, DNA flow cytometry was used to determine the cell population in each cell phase following treatment with a cytotoxic agent. As seen in Fig. 7B, compound **18c** treatment significantly increased cells at the S-phase by 50.5% compared to control 37.8%, while cells in G0-G1 were not significantly increased, and cells in G2/M were decreased. Consequently, compound **18c** induced apoptosis in HepG2 cells arresting the cell proliferation at S-phase.

##### RT-PCR

**Fig. 8:**
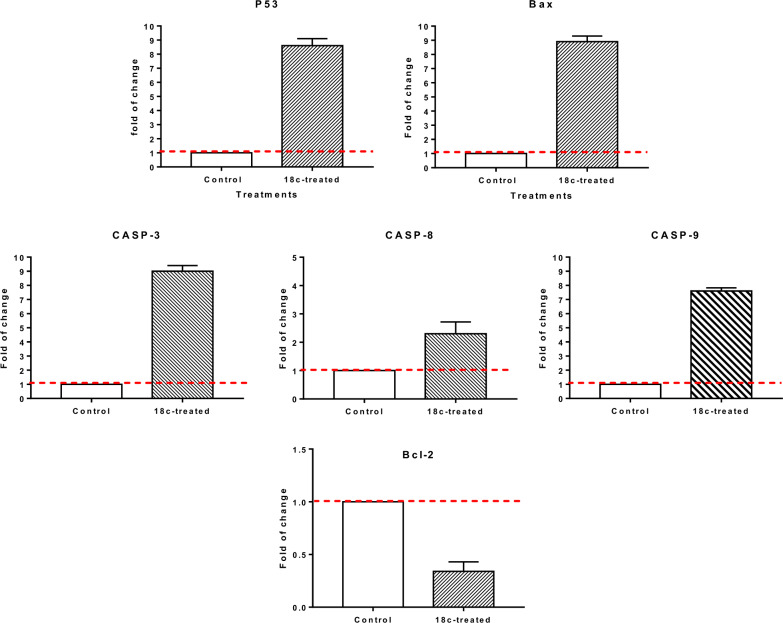
Quantitative RT-PCR results analysis of the apoptosis-related genes; P53, Bax, Caspases 3, 8, 9, and Bcl-2, respectively, in HepG2 cells treated with **18c** with the IC_50_ values, 48 h. The data illustrated is the average of 3 independent experimental runs (Mean ± SD). The red dashed line represents the fold change of control = 1

For validating the apoptotic cell death in HepG2 cells upon treatment with compound **18c,** gene expression level using RT-PCR was investigated for the apoptosis-mediated genes of P53, Bax, caspase-3,8,9 and Bcl-2 in both untreated and treated HepG2 cells. As seen in Fig. 8, compound **18c**, upreguated P53 by 8.6-fold, Bax by 8.9-fold, caspase-3,8,9 by 9, 2.3, and 7.6-fold, while it inhibted the Bcl-2 expression by 0.34-fold. Therefore, our results demonstrated that the intrinsic mechanism of apoptosis was responsible for the cell death induced by treatment with compound **18c**. Caspases are involved in both the beginning and end of the death process in mitochondria-mediated apoptosis. Loss of mitochondrial potential can be triggered by upregulating proapoptotic subunits over antiapoptotic proteins like Bcl-2 protein. By increasing proapoptotic proteins and decreasing antiapoptotic proteins, the intrinsic apoptotic pathway is activated, and mitochondria lose their mitochondrial potential (ΔΨm), releasing cytochrome c, which triggered caspase 3 and 9 activations and, ultimately, cell death via caspase-dependent apoptosis.

## Experimental section

### Chemistry

Melting points were determined in open glass capillaries with a Gallenkamp apparatus. Elemental analyses were carried out at the Microanalytical Center of Cairo University, Giza, Egypt. The infrared spectra were recorded as potassium bromide disks on a Pye Unicam SP 3–300 and Shimadzu FTIR 8101 PC infrared spectrophotometer. NMR spectra were recorded on Varian Mercury VXR-300 NMR spectrometer at 300 MHz (1H NMR) and at 75 MHz (13C NMR) using DMSO-*d*6 as solvent Chemical shifts were reported downfield from TMS (= 0) for ^1^H NMR. For ^13^C NMR, chemical shifts were reported in the scale relative to the solvent used as an internal reference. Mass spectra (EI) were obtained at 70 eV with a type Shimadzu GCMQP 1000 EX spectrometer. The enaminone **3**, [55] hydrazonoyl chloride **4**, [56] aldehyde-thiosemicarbazones **7a-c**, [57, 58] acetyl-thiosemicarbazones **9, 10, 11** [59, 60], bis-aldehydes **15a-c**, **19a-c** [61–63]**,** and bis-thiosemicarbazones **17a-c**, **20a-c**, [64–67] were prepared following procedures reported in the literature.

#### Synthesis of benzylidenehydrazinyl-(1H-pyrazol-3-yl)thiazole derivatives 8a–d

##### General procedure

To a mixture of 3-bromoacetyl-4-(2-naphthoyl)-1-phenyl-1H-pyrazole **(6)** (1.0 mmol) and the appropriate aldehyde-thiosemicarbazone derivatives **7a-d** (1.0 mmol) in ethanol (20 mL), triethylamine (0.2 mL) were added, and the mixture was heated under reflux for 3–5 h. The reaction mixture was allowed to cool to room temperature, and the solvent was evaporated under vacuum. The solid residue was collected by filtration and recrystallized from ethanol/DMF to give the corresponding 2-(benzylidenehydrazinyl)-4-(pyrazol-3-yl)thiazole derivatives **8a–d.**

*2-(Benzylidenehydrazinyl)-4-(4-(2-naphthoyl)-1-phenyl-pyrazol-3-yl)thiazole (8a)*

Yellow crystals, (88% yield), mp. 138–140 °C; IR (KBr) 3437 (NH), 1647 (C = O), 1576 (C = N) cm^−1^; ^1^H-NMR δ 7.35–7.66 (m, 11H, ArH), 7.95 (s, 1H, thiazole-5-H), 7.98–8.03 (m, 3H, ArH), 8.06 (s, 1H, CH = N), 8.12 (d, 2H, ArH, *J* = 7.8), 8.51 (s, 1H, naphthalene-1-H), 9.02 (s, 1H, pyrazole-5-H), 12.09 (s, 1H, NH); ^13^C NMR: δ 108.4, 119.0, 120.9, 124.7, 126.2, 126.8, 127.1, 127.6, 128.2, 128.5, 128.8, 129.2, 129.6, 129.8, 131.6, 132.1, 132.7, 134.3, 134.9, 135.7, 138.9, 141.1, 147.9, 167.6, 189.0; MS: *m/z* 499 (M^+^). Anal. Calcd. for C_30_H_21_N_5_OS: C, 72.12; H, 4.24; N, 14.02; S, 6.42. Found: C, 72.10; H, 4.22; N, 14.03; S, 6.39%.

*2-(4-Methylbenzylidenehydrazinyl)-4-(4-(2-naphthoyl)-1-phenylpyrazol-3-yl)thiazole (8b)*

Brown powder, (83% yield), mp. 207–210 °C; IR (KBr) 3432 (NH), 1648 (C = O), 1578 (C = N) cm^−1^; ^1^H-NMR: δ 2.31 (s, 3H, CH_3_), 7.21 (d, 2H, ArH, *J* = 7.8), 7.37–7.68 (m, 8H, ArH), 7.91 (s, 1H, thiazole-5-H), 7.96–8.03 (m, 3H, ArH), 8.06 (s, 1H, CH = N), 8.12 (d, 2H, ArH, *J* = 8.1), 8.51 (s, 1H, naphthalene-1-H), 9.01 (s, 1H, pyrazole-5-H), 11.96 (s, 1H, NH); ^13^C NMR: δ 23.4, 102.5, 114.9, 115.7, 116.1, 118.3, 121.1, 127.3, 128.4, 128.5, 128.6, 128.7, 128.8, 129.0, 129.2, 130.1, 130.5, 130.8, 131.1, 131.5, 135.5, 136.1, 138.1, 141.1, 147.9, 169.2, 191.1. MS: *m/z* 513 (M^+^). Anal. Calcd. for C_31_H_23_N_5_OS: C, 72.49; H, 4.51; N, 13.64; S, 6.24. Found: C, 72.44; H, 4.52; N, 13.61; S, 6.23%.

*2-(4-Methoxybenzylidenehydrazinyl)-4-(4-(2-naphthoyl)-1-phenyl-pyrazol-3-yl)thiazole (8c)*

Brown powder, (85% yield), mp. 121–123 °C; IR (KBr) 3408 (NH), 1600 (C = O), 1509 (C = N) cm^−1^; ^1^H-NMR: δ 3.79 (s, 3H, OCH_3_), 6.97 (d, 2H, ArH, *J* = 8.7), 7.39–7.66 (m, 8H, ArH), 7.89 (s, 1H, thiazole-5-H), 7.95–8.00 (m, 3H, ArH), 8.03 (s, 1H, CH = N), 8.09 (d, 2H, ArH, *J* = 8.4), 8.51 (s, 1H, naphthalene-1-H), 9.01 (s, 1H, pyrazole-5-H), 11.93 (s, 1H, NH); MS: *m/z* 529 (M^+^). Anal. Calcd. for C_31_H_23_N_5_O_2_S: C, 70.30; H, 4.38; N, 13.22; S, 6.05. Found: C, 70.27; H, 4.35; N, 13.24; S, 6.03%.

*2-(4-Nitrobenzylidenehydrazinyl)-4-(4-(2-naphthoyl)-1-phenyl-pyrazol-3-yl)thiazole (8d)*

Red powder, (81% yield), mp. 231–233 °C; IR (KBr) 3430 (NH), 1643 (C = O), 1547 (C = N) cm^−1^; ^1^H-NMR: δ 7.54 (d, 2H, ArH, *J* = 9.3), 7.58–7.87 (m, 8H, ArH), 7.98 (s, 1H, thiazole-5-H), 8.01–8.04 (m, 3H, ArH), 8.05 (s, 1H, CH = N), 8.24 (d, 2H, ArH, *J* = 9), 8.52 (s, 1H, naphthalene-1-H), 9.03 (s, 1H, pyrazole-5-H), 12.48 (s, 1H, NH); ^13^C NMR: δ 109.2, 119.1, 120.9, 124.1, 124.7, 126.8, 126.9, 127.2, 127.6, 128.2, 128.6, 129.1, 129.6, 129.8, 131.6, 132.1, 132.8, 134.9, 135.6, 138.6, 138.9, 140.8, 147.0, 147.7, 167.2, 188.9; MS: *m/z* 544 (M^+^). Anal. Calcd. for C_30_H_20_N_6_O_3_S: C, 66.16; H, 3.70; N, 15.43; S, 5.89. Found: C, 66.15; H, 3.67; N, 15.41; S, 5.87%.

#### Synthesis of arylidenehydrazinyl-(1H-pyrazol-3-yl)thiazole derivatives 12–14

##### General procedure

To a solution of 3-bromoacetyl-4-(2-naphthoyl)-1-phenyl-1H-pyrazole **6** (1 mmol) in ethanol (25 mL) containing triethylamine (0.2 ml), the appropriate heterocyclic-thiosemicarbazone derivatives **9–11** (1 mmol) was added. The reaction mixture was heated at reflux for 5 h and then left to cool to room temperature. The obtained solid products were filtered off, then recrystallized from ethanol/DMF to afford the 2-(ethylidenehydrazinyl)-4-(4-(2-naphthoyl)-1-phenylpyrazol-3-yl)thiazole derivatives **12–14.**

*2-(2-(1-(Benzofuran-2-yl)ethylidene)hydrazinyl)-4-(4-(2-naphthoyl)-1-phenylpyrazol-3-yl)thiazole (12)*

Brown powder, (85% yield), mp. 164–166 °C; IR (KBr) 3433 (NH), 1648 (C = O), 1539 (C = N) cm^−1^; ^1^H-NMR: δ 2.31 (s, 3H, CH_3_), 7.22 (s, 1H, benzofuran-3-H), 7.25–7.67 (m, 9H, ArH), 7.89 (s, 1H, thiazole-5-H), 7.93–8.07 (m, 4H, ArH), 8.14 (d, 2H, ArH, *J* = 7.8), 8.55 (s, 1H, naphthalene-1-H), 9.04 (s, 1H, pyrazole-5-H), 11.59 (s, 1H, NH); ^13^C NMR: δ 17.0, 103.4, 117.6, 118.4, 118.9, 119.3, 119.8, 125.9, 126.8, 127.7, 127.9, 128.5, 128.8, 129.1, 129.3, 129.6, 130.0, 130.2, 131.2, 131.1, 142.5, 151.6, 153.0, 154.1, 155.2, 158.4, 159.9, 160.3, 172.2, 188.0. MS: *m/z* 553 (M^+^). Anal. Calcd. for C_33_H_23_N_5_O_2_S: C, 71.59; H, 4.19; N, 12.65; S, 5.79. Found: C, 71.56; H, 4.18; N, 12.63; S, 5.77%.

*2-(2-(1-(Benzothiazol-2-yl)ethylidene)hydrazinyl)-4-(4-(2-naphthoyl)-1-phenylpyrazol-3-yl)-thiazole (13)*

Creamy powder, (87% yield), mp. 143–145 °C; IR (KBr) 3410 (NH), 1635 (C = O), 1543 (C = N) cm^−1^; ^1^H-NMR: δ 2.45 (s, 3H, CH_3_), 7.36 (s, 1H, thiazole-5-H), 7.39–8.06 (m, 13H, ArH), 8.12 (d, 2H, ArH, *J* = 8.1), 8.55 (s, 1H, naphthalene-1-H), 9.04 (s, 1H, pyrazole-5-H), 12.04 (s, 1H, NH); ^13^C NMR: δ 13.3, 110.2, 119.1, 120.7, 122.1, 122.9, 124.8, 125.8, 126.2, 126.8, 127.3, 127.6, 128.2, 128.5, 129.6, 129.8, 131.7, 132.1, 133.5, 134.9, 135.0, 135.7, 138.9, 153.2, 168.1, 188.8; MS: *m/z* 570 (M^+^). Anal. Calcd. for C_32_H_22_N_6_OS_2_: C, 67.35; H, 3.89; N, 14.73; S, 11.24. Found: C, 67.34; H, 3.86; N, 14.75; S, 11.23%.

*2-(2-(1-(Coumarin-3-yl)ethylidene)hydrazinyl)-4-(4-(2-naphthoyl)-1-phenylpyrazol-3-yl)-thiazole (14)*

Yellow powder, (73% yield), mp. 239–241 °C; IR (KBr) 3448 (NH), 1635 (C = O), 1519 (C = N) cm^−1^; ^1^H-NMR: δ 2.23 (s, 3H, CH_3_), 7.34–8.14 (m, 17H, ArH, thiazole-5-H and chromen-4-H), 8.53 (s, 1H, naphthalene-1-H), 9.03 (s, 1H, pyrazole-5-H), 11.39 (s, 1H, NH); MS: *m/z* 581 (M^+^). Anal. Calcd. for C_34_H_23_N_5_O_3_S: C, 70.21; H, 3.99; N, 12.04; S, 5.51. Found: C, 70.20; H, 3.96; N, 12.03; S, 5.53%.

#### Synthesis of bis((2-(4-((2-naphthoyl)-1-phenyl-1H-pyrazol-3-yl)thiazol-2-yl)hydrazono)-methyl)phenoxy)alkanes 18a-c and 21a-c

##### General procedure

To a mixture of the appropriate 4,4ʹ-(alkane-diylbis(oxy))dibenzaldehydethiosemicarbazone derivatives **17a-c** or 2,2ʹ-(alkane-diylbis(oxy))dibenzaldehydethiosemicarbazone derivatives **19a-c** (1.0 mmol) and 3-bromoacetyl-4-(2-naphthoyl)-1-phenyl-1H-pyrazole **(6)** (2.0 mmol) in ethanol (25 mL), triethylamine (0.2 mL) were added. The reaction mixture was heated under reflux temperature for 4 ~ 6 h and then allowed to cool to room temperature. The solvent was then evaporated under vacuum, and the solid residue was collected by filtration and recrystallized from ethanol/DMF to give the corresponding bis-pyrazolylthiazoles **18a-c** and **21a-c**.

*1,2-Bis(4-((2-(4-((2-naphthoyl)-1-phenyl-1H-pyrazol-3-yl)thiazol-2-yl)hydrazono)methyl)-phenoxy)ethane (18a)*

Off-white powder, (79% yield), mp. 187–189 °C; IR (KBr) 3407 (NH), 1645 (C = O), 1505 (C = N) cm^−1^; ^1^H-NMR: δ 4.35 (s, 4H, CH_2_O), 7.03 (d, 4H, ArH, *J* = 7.8), 7.36–7.68 (m, 16H, ArH), 7.91 (s, 2H, thiazole-5-H), 7.98–8.02 (m, 6H, ArH), 8.06 (s, 2H, CH = N), 8.11 (d, 4H, ArH, *J* = 7.8), 8.51 (s, 2H, naphthalene-1-H), 9.01 (s, 2H, pyrazole-5-H), 11.90 (s, 2H, NH); ^13^C NMR: δ 66.4, 108.1, 114.8, 119.0, 120.9, 124.7, 126.8, 127.1, 127.2, 127.6, 127.7, 128.2, 128.5, 128.9, 129.6, 129.8, 131.6, 132.1, 132.7, 134.9, 135.7, 138.9, 141.1, 148.0, 159.2, 167.7, 189.0; MS: *m/z* 529 (M^+^/2). Anal. Calcd. for C_62_H_44_N_10_O_4_S_2_: C, 70.44; H, 4.19; N, 13.25; S, 6.07. Found: C, 70.45; H, 4.17; N, 13.25; S, 6.06%.

*1,3-Bis(4-((2-(4-((2-naphthoyl)-1-phenyl-1H-pyrazol-3-yl)thiazol-2-yl)hydrazono)methyl)-phenoxy)propane (18b)*

Off-white powder, (80% yield), mp. 200–202 °C; IR (KBr) 3431 (NH), 1634 (C = O), 1508 (C = N) cm^−1^; ^1^H-NMR: δ 2.21 (m, 2H, CH_2_), 4.17 (m, 4H, CH_2_O), 6.99 (d, 4H, ArH, *J* = 8.1), 7.39–7.65 (m, 16H, ArH), 7.89 (s, 2H, thiazole-5-H), 7.98 (m, 6H, ArH), 8.05 (s, 2H, CH = N), 8.11 (d, 4H, ArH, *J* = 7.8), 8.50 (s, 2H, naphthalene-1-H), 9.01 (s, 2H, pyrazole-5-H), 11.95 (s, 2H, NH); ^13^C NMR: δ 27.4, 67.0, 103.4, 109.1, 114.5, 118.5, 119.9, 121.2, 127.3, 128.6, 128.7, 129.0, 129.1, 129.5, 129.6, 130.2, 133.7, 136.2, 137.2, 137.4, 148.9, 149.9, 157.2, 160.2, 162.0, 166.4, 189.9; MS: *m/z* 529 (M^+^/2). Anal. Calcd. for C_63_H_46_N_10_O_4_S_2_: C, 70.64; H, 4.33; N, 13.08; S, 5.99. Found: C, 70.63; H, 4.30; N, 13.07; S, 5.97%.

*1,4-Bis(4-((2-(4-((2-naphthoyl)-1-phenyl-1H-pyrazol-3-yl)thiazol-2-yl)hydrazono)methyl)-phenoxy)butane (18c)*

Off-white powder, (83% yield), mp. 223–225 °C; IR (KBr) 3435 (NH), 1647 (C = O), 1566 (C = N) cm^−1^; ^1^H-NMR: δ 2.24 (m, 4H, CH_2_), 4.36 (m, 4H, CH_2_O), 7.02 (d, 4H, ArH, *J* = 7.5), 7.39–7.70(m, 16H, ArH), 7.87–7.91 (m, 8H, thiazole-5-H, ArH), 7.98 (s, 2H, CH = N), 8.13 (d, 4H, ArH, *J* = 6.9), 8.53 (s, 2H, naphthalene-1-H), 9.02 (s, 2H, pyrazole-5-H), 11.11 (s, 2H, NH); MS: *m/z* 543 (M^+^/2). Anal. Calcd. For C_64_H_48_N_10_O_4_S_2_: C, 70.83; H, 4.46; N, 12.91; S, 5.91. Found: C, 70.81; H, 4.45; N, 12.91; S, 5.90.

*1,2-Bis(2-((2-(4-((2-naphthoyl)-1-phenyl-1H-pyrazol-3-yl)thiazol-2-yl)hydrazono)methyl)-phenoxy)ethane (20a)*

Off-white powder, (71% yield), mp. 166–168 °C; IR (KBr) 3432 (NH), 1645 (C = O), 1571 (C = N) cm^−1^; ^1^H-NMR: δ 4.40 (s, 4H, CH_2_O), 7.13 (d, 4H, ArH, *J* = 7.5), 7.36–7.77 (m, 16H, ArH), 7.95 (s, 2H, thiazole-5-H), 7.98 (m, 6H, ArH), 8.06 (d, 4H, ArH, *J* = 8.7), 8.29 (s, 2H, CH = N), 8.45 (s, 2H, naphthalene-1-H), 8.99 (s, 2H, pyrazole-5-H), 11.99 (s, 2H, NH); ^13^C NMR: δ 67.7, 102.1, 109.1, 112.1, 115.1, 116.7, 117.0, 119.8, 121.0, 124.8, 127.3, 127.6, 127.7, 129.1, 129.3, 131.7, 135.4, 136.4, 138.7, 139.2, 141.0, 144.0, 153.4, 156.4, 162.2, 148.0, 170.1, 192.0; MS: *m/z* 529 (M^+^/2) Anal. Calcd. for C_62_H_44_N_10_O_4_S_2_: C, 70.44; H, 4.19; N, 13.25; S, 6.07. Found: C, 70.45; H, 4.17; N, 13.25; S, 6.06%.

*1,3-Bis(2-((2-(4-((2-naphthoyl)-1-phenyl-1H-pyrazol-3-yl)thiazol-2-yl)hydrazono)methyl)-phenoxy)propane (20b)*

Off-white powder, (75% yield), mp. 209–211 °C; IR (KBr) 3429 (NH), 1645 (C = O), 1573 (C = N) cm^−1^; ^1^H-NMR: δ 2.22 (m, 2H, CH_2_), 4.24(m, 4H, CH_2_O), 7.08 (d, 4H, ArH, *J* = 6.6), 7.29–7.75 (m, 16H, ArH), 7.90 (s, 2H, thiazole-5-H), 7.98–8.06 (m, 6H, ArH), 8.11 (d, 4H, ArH, *J* = 7.5), 8.33 (s, 2H, CH = N), 8.50 (s, 2H, naphthalene-1-H), 9.01 (s, 2H, pyrazole-5-H), 11.99 (s, 2H, NH); MS: *m/z* 529 (M^+^/2). Anal. Calcd. for C_63_H_46_N_10_O_4_S_2_: C, 70.64; H, 4.33; N, 13.08; S, 5.99. Found: C, 70.63; H, 4.30; N, 13.07; S, 5.97%.

*1,4-Bis(2-((2-(4-((2-naphthoyl)-1-phenyl-1H-pyrazol-3-yl)thiazol-2-yl)hydrazono)methyl)-phenoxy)butane (20c)*

Off-white powder, (84% yield), mp. 230–232 °C; IR (KBr) 3439 (NH), 1601 (C = O), 1507 (C = N) cm^−1^; ^1^H-NMR: δ 1.94 (m, 4H, CH_2_), 4.09 (m, 4H, CH_2_O), 7.04(d, 4H, ArH, *J* = 8.1), 7.32–7.75 (m, 16H, ArH), 7.97 (s, 2H, thiazole-5-H), 7.99 (m, 6H, ArH), 8.08 (d, 4H, ArH, *J* = 7.5), 8.34 (s, 2H, CH = N), 8.49 (s, 2H, naphthalene-1-H), 9.00 (s, 2H, pyrazole-5-H), 12.02 (s, 2H, NH); MS: *m/z* 543 (M^+^/2). Anal. Calcd. for C_64_H_48_N_10_O_4_S_2_: C, 70.83; H, 4.46; N, 12.91; S, 5.91. Found: C, 70.81; H, 4.45; N, 12.91; S, 5.90.

### Biological part

#### Cytotoxicity

HepG2 liver cancer cells and THLE2 normal liver cells were bought from the National Research Institute in Egypt and cultured in “RPMI-1640” media containing L-Glutamine (Lonza Verviers SPRL, Belgium, cat#12-604F). Fetal bovine serum (Sigma-Aldrich, MO, USA) and penicillin–streptomycin at a concentration of 10% and 1%, respectively, were added to both cell lines (Lonza, Belgium). All cells were incubated following routine tissue culture work. Cells were treated with the compounds at (0.01, 0.1, 1, 10, and 100 µM) concentrations. Cell viability was assessed after 48 h using MTT solution (Promega, USA) [68]. Finally, Absorbance was subsequently measured (at 570 nm) using ELISA microplate reader (BIO-RAD, model iMark, Japan). The percentage of cell viability was calculated, and IC_50_ values were recorded using the GraphPad prism 7, as previously reported in the literature [69, 70].

#### EGFR and HER2 kinase inhibitory assay

Anti-EGFR and anti-HER2 activities were measured using EGFR Kinase Assay Kit “BPS Bioscience kit, Cat#40321” and HER2 Kinase Assay Kit “BPS Bioscience kit, Cat# 40721”. Kinase inhibitory assays were performed to evaluate the inhibitory potency of compounds **18b, 18c,** and **21a** against the EGFR and HER2. The autophosphorylation percentage inhibition of compounds was calculated by: $100-[\frac{A control}{A treated}-Control)]$. GraphPad prism7 was used to determine IC_50_ from percent inhibition curves at five doses of each compound [71].

#### Investigation of apoptosis

##### Annexin V/PI staining and cell cycle analysis

Overnight, 6-well culture plates were stocked with HepG2 cells (3–5 × 10^5^ cells/well). After determining the IC_50_ values, cells were treated with compound **18c** for 48 h. After that, the cells and the medium supernatants were separated and washed with ice-cold PBS. The next step was suspending the cells in 100 µL of annexin binding buffer solution "25 mM CaCl2, 1.4 M NaCl, and 0.1 M Hepes/NaOH, pH 7.4″ and incubation with “Annexin V-FITC solution (1:100) and propidium iodide (PI)” at a concentration equals 10 µg/mL in the dark for 30 min. After the cells were stained, they were gathered by Cytoflex FACS equipment. We used cytExpert to examine the data [72–74].

##### Real-time-polymerase chain reaction for the selected genes

Genes of P53, Bax, and Caspases-3,8,9 were screened as proapoptotic genes, while Bcl-2 was screened as antiapoptotic, therefore, we measured their gene expression to investigate the apoptotic process. HepG2 cells were then treated with compound **18c** at their IC_50_ values for 48 h. After treatment, RT-PCR reaction was carried out following routine work. The Ct values were then used to determine the fold change in gene expression between samples after normalization to the β-actin reference gene [72, 75].

## Conclusions

In this article, we introduced 2-naphthoyl moiety into the 3-position of the pyrazolyl-thiazole backbone to generate novel combined naphthalene-pyrazole-aminothiazole hybrids as simple and bis-forms. The results revealed that some of the obtained molecular hybrids had remarkable potential as anti-cancer agents. Investigating the cytotoxicity, compound **18c** showed potent cytotoxicity with an IC_50_ value of 0.97 µM compared to Lapatinib (IC_50_ = 7.45 µM). Additionally, they were safe (non-cytotoxic) against the THLE2 cells. Compounds **18c** exhibited promising EGFR/HER2 inhibitory activities with IC_50_ values of 4.98 and 9.85 nM, respectively, compared to Lapatinib (IC_50_ = 6.1 and 17.2 nM). Compound **18c** significantly activated liver apoptotic cell death, increasing the death rate by 63.6-fold and arresting cell proliferation at S-phase. Additionally, compound **18c** affected the apoptosis-related genes by upregulating the proapoptotic and downregulating the antiapoptotic gene. Thereby compound **18c** exhibited promising cytotoxicity against HepG2 cells through EGFR/HER2 inhibition. Interestingly, doubling the molecular hybrids in the form of bis-heterocycles led to an extraordinary improvement in the cytotoxic efficiency compared with the simple molecular hybrids, where doubling the pharmacophoric scaffold led to about an eightfold increase in the cytotoxic potency (IC_50_ = 0.97 µM for **18c** vs. 7.39 µM for **8c**).

## Supplementary Information

**Additional file 1: Figure S1.** 1H NMR spectrum of compound 6. **Figure S2.** 1H NMR spectrum of compound 8a. **Figure S3.** 13C NMR spectrum of compound 8a. **Figure S4.** 1H NMR spectrum of compound 8b. **Figure S5.** 13C NMR spectrum of compound 8b. **Figure S6.** 1H NMR spectrum of compound 8c. **Figure S7.** 1H NMR spectrum of compound 8d. **Figure S8.** 13C NMR spectrum of compound 8d. **Figure S9.** 1H NMR spectrum of compound 12. **Figure S10.** 13CNMR spectrum of compound 12. **Figure S11.** 1H NMR spectrum of compound 13. **Figure S12.** 13C NMR spectrum of compound 13. **Figure S13.** 1H NMR spectrum of compound 14. **Figure S14**. 1H NMR spectrum of compound 18a. **Figure S15.** 13C NMR spectrum of compound 18a. **Figure S16.** 1H NMR spectrum of compound 18b. **Figure S17.** 13C NMR spectrum of compound 18b. **Figure S18.** 1H NMR spectrum of compound 18c. **Figure S19.** 1HNMR spectrum of compound 20a. **Figure S20.** 13C NMR spectrum of compound 20a. **Figure S21.** 1H NMR spectrum of compound 20b. **Figure S22.** 1H NMR spectrum of compound 20c. **Figure S23.** IR spectrum of compound 8a. **Figure S24.** Mass spectrum of compound 8a. **Figure S25.** IR spectrum of compound 8b. **Figure S26.** Mass spectrum of compound 8b. **Figure S27.** IR spectrum of compound 8c. **Figure S28.** Mass spectrum of compound 8c. **Figure S29.** IR spectrum of compound 12. **Figure S30.** Mass spectrum of compound 12. **Figure S31.** IR spectrum of compound 13. **Figure S32.** Mass spectrum of compound 13. **Figure S33.** IR spectrum of compound 14. **Figure S34.** Mass spectrum of compound 14. **Figure S35.** IR spectrum of compound 18b. **Figure S36.** Mass spectrum of compound 18b. **Figure S37.** IR spectrum of compound 18c. **Figure S38.** Mass spectrum of compound 18c. **Figure S39**. IR spectrum of compound 20a. **Figure S40.** Mass spectrum of compound 20a. **Figure S41.** IR spectrum of compound 20b. **Figure S42.** Mass spectrum of compound 20b. **Figure S43.** IR spectrum of compound 20c. **Figure S44.** Mass spectrum of compound 20c.

## Data Availability

All data and analyses are available from the corresponding author on reasonable request.

## Abbreviations

EGFR: Epidermal growth factor receptor
HER2: Human epidermal growth factor receptor 2
RT-PCR: Reverse transcription polymerase chain reaction (RT-PCR)
ΔΨm: Mitochondrial potential; Bcl-2: B-cell lymphoma 2
Bax: Bcl-2-associated X protein
SD: Standard deviation
IC_50_: Half-maximal inhibitory concentration
NSCLC: Non-small cell lung carcinoma;
HepG2: Liver cancer cells
THLE2: Normal liver cells
G2/M, S, G1, G0: Cell cycle phases
