# Supplementary material for: Synthesis of novel mono- and bis-pyrazolylthiazole derivatives as anti-liver cancer agents through EGFR/HER2 target inhibition
Source: BMC Chem. 2023 Jun 8;17(1):51. doi: 10.1186/s13065-023-00921-6 (PMC10251674; doi:10.1186/s13065-023-00921-6)
Supplement: Supplementary file 1 — Additional file 1: Figure S1. 1H NMR spectrum of compound 6. Figure S2. 1H NMR spectrum of compound 8a. Figure S3. 13C NMR spectrum of compound 8a. Figure S4. 1H NMR spectrum of compound 8b. Figure S5. 13C NMR spectrum of compound 8b. Figure S6. 1H NMR spectrum of compound 8c. Figure S7. 1H NMR spectrum of compound 8d. Figure S8. 13C NMR spectrum of compound 8d. Figure S9. 1H NMR spectrum of compound 12. Figure S10. 13CNMR spectrum of compound 12. Figure S11. 1H NMR spectrum of compound 13. Figure S12. 13C NMR spectrum of compound 13. Figure S13. 1H NMR spectrum of compound 14. Figure S14. 1H NMR spectrum of compound 18a. Figure S15. 13C NMR spectrum of compound 18a. Figure S16. 1H NMR spectrum of compound 18b. Figure S17. 13C NMR spectrum of compound 18b. Figure S18. 1H NMR spectrum of compound 18c. Figure S19. 1HNMR spectrum of compound 20a. Figure S20. 13C NMR spectrum of compound 20a. Figure S21. 1H NMR spectrum of compound 20b. Figure S22. 1H NMR spectrum of compound 20c. Figure S23. IR spectrum of compound 8a. Figure S24. Mass spectrum of compound 8a. Figure S25. IR spectrum of compound 8b. Figure S26. Mass spectrum of compound 8b. Figure S27. IR spectrum of compound 8c. Figure S28. Mass spectrum of compound 8c. Figure S29. IR spectrum of compound 12. Figure S30. Mass spectrum of compound 12. Figure S31. IR spectrum of compound 13. Figure S32. Mass spectrum of compound 13. Figure S33. IR spectrum of compound 14. Figure S34. Mass spectrum of compound 14. Figure S35. IR spectrum of compound 18b. Figure S36. Mass spectrum of compound 18b. Figure S37. IR spectrum of compound 18c. Figure S38. Mass spectrum of compound 18c. Figure S39. IR spectrum of compound 20a. Figure S40. Mass spectrum of compound 20a. Figure S41. IR spectrum of compound 20b. Figure S42. Mass spectrum of compound 20b. Figure S43. IR spectrum of compound 20c. Figure S44. Mass spectrum of compound 20c. [file 13065_2023_921_MOESM1_ESM.pdf]

Additional Information

Synthesis of novel mono- and bis-pyrazolylthiazole derivatives as anti-liver cancer agents through EGFR/HER2 target inhibition

Mostafa E. Salem,[a,b] Esraa M. Mahrous,[a] Eman A. Ragab,[a] Mohamed S. Nafie,[c] and Kamal M. Dawood*[a]

[a] Department of Chemistry, Faculty of Science, Cairo University, Giza, 12613, Egypt.

[b] Department of chemistry, College of Science, Imam Mohammad Ibn Saud Islamic University (IMSIU), Riyadh, Saudi Arabia.

[c] Department of Chemistry, Faculty of Science, Suez Canal University, Ismailia, 41522, Egypt.

*Corresponding author: Kamal M. Dawood: E-Mail: kmdawood@sci.cu.edu.eg,

Tel: (+202) 35676602, Fax: (+202) 35727556; ORCID: https://orcid.org/0000-0002-1351-9886

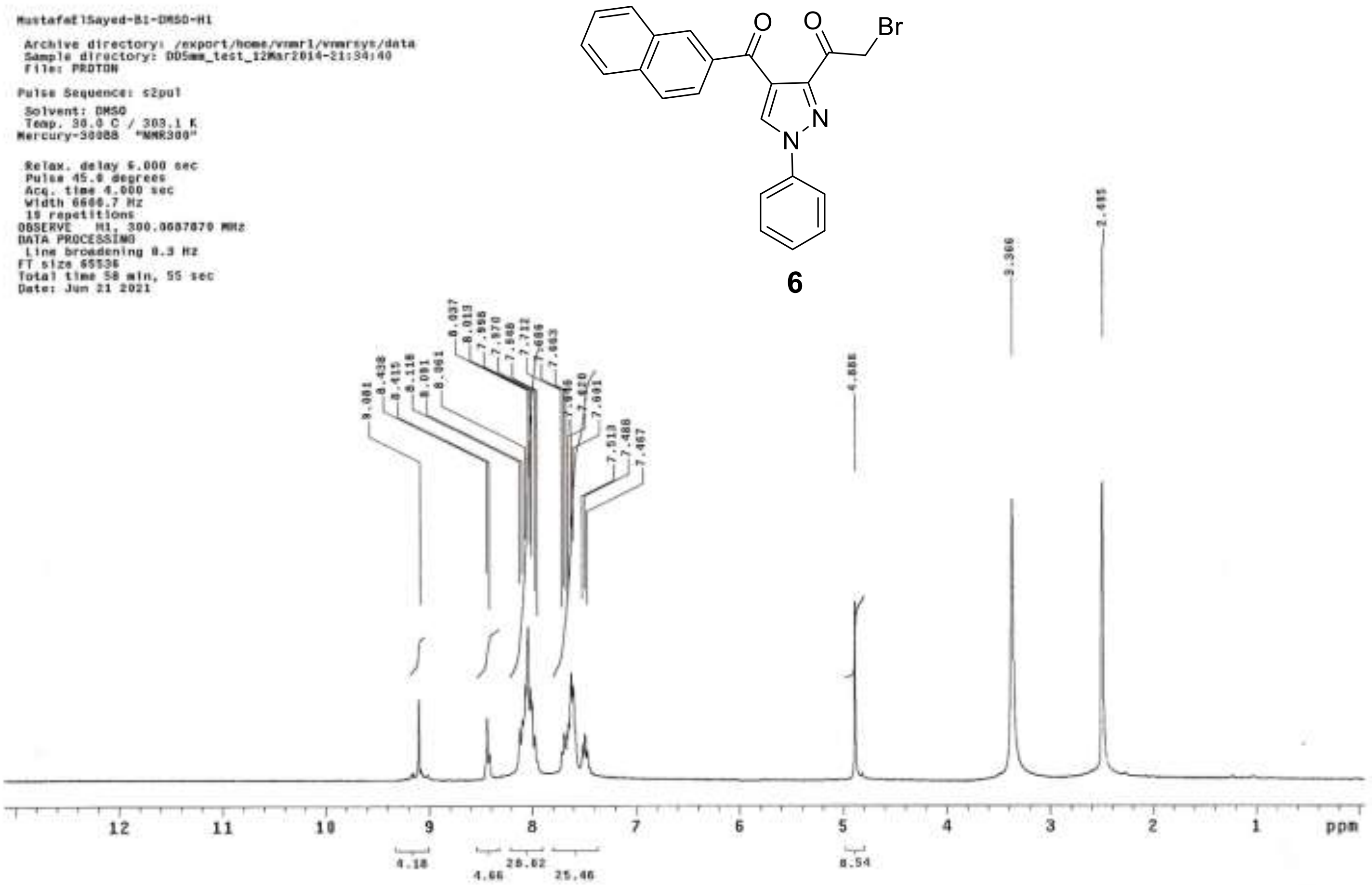

Figure S1: ^{1}H NMR spectrum of compound 6

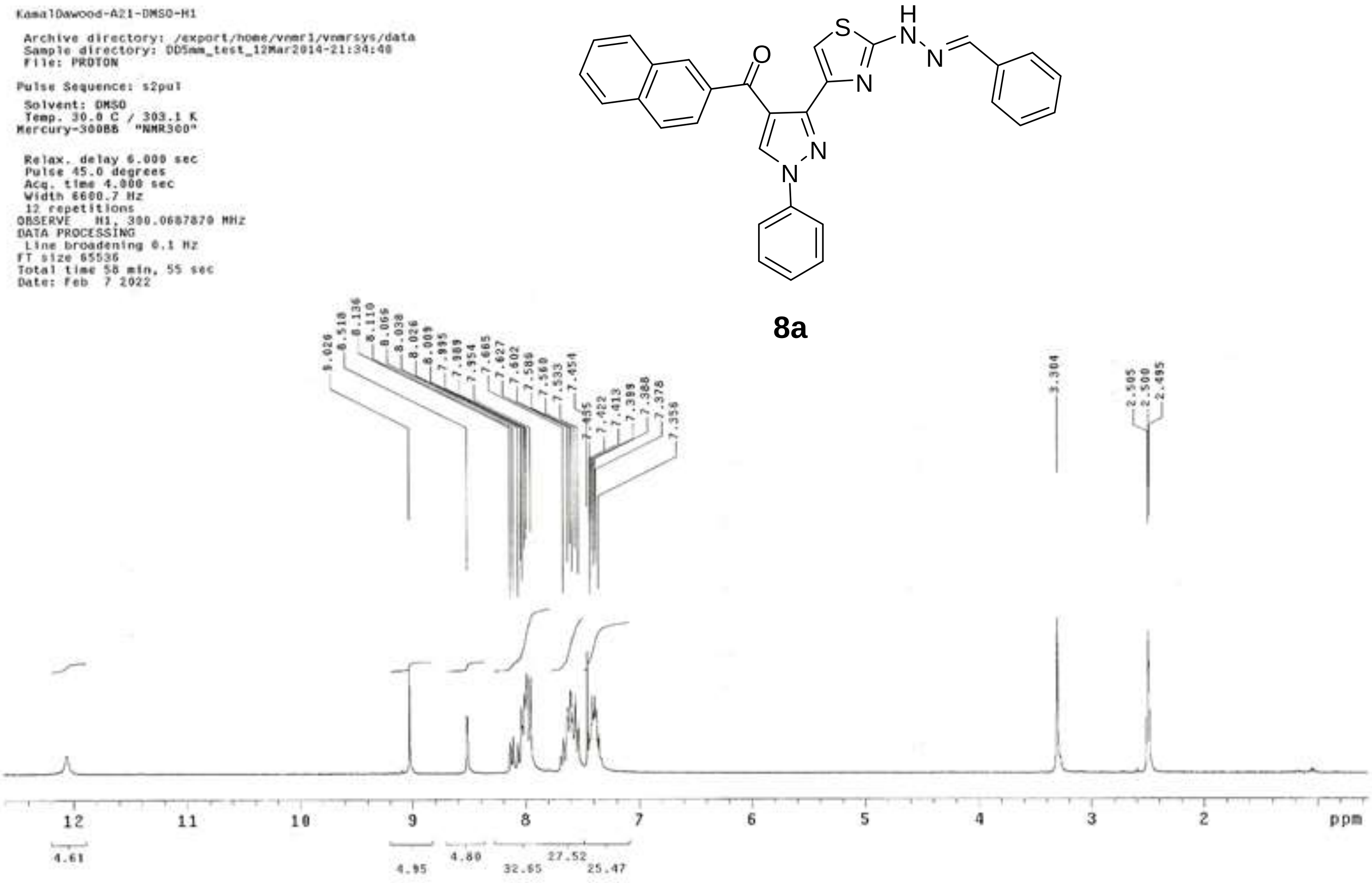

Figure S2: ^{1}H NMR spectrum of compound 8a

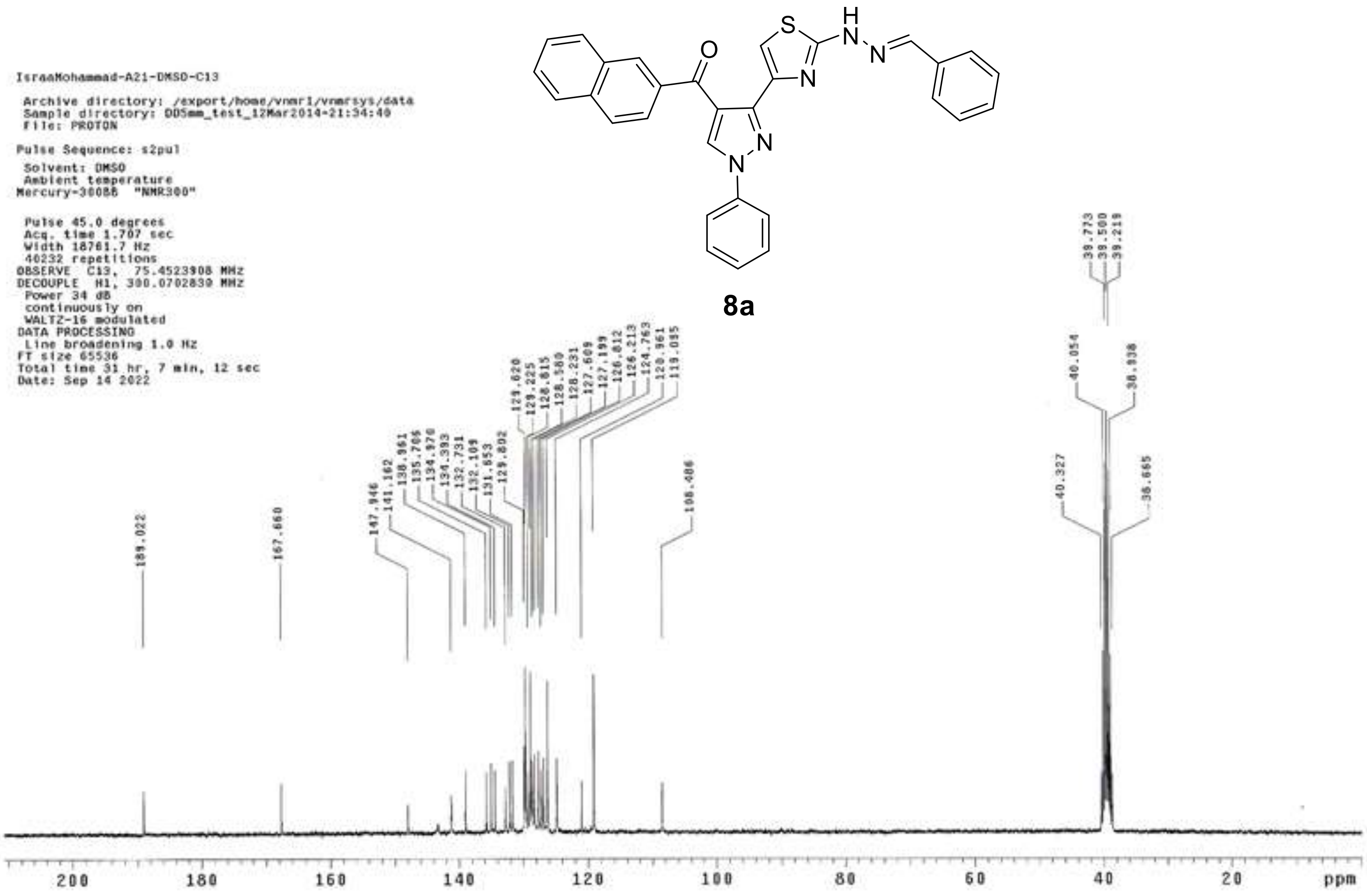

Figure S3: ^{13}C NMR spectrum of compound 8a

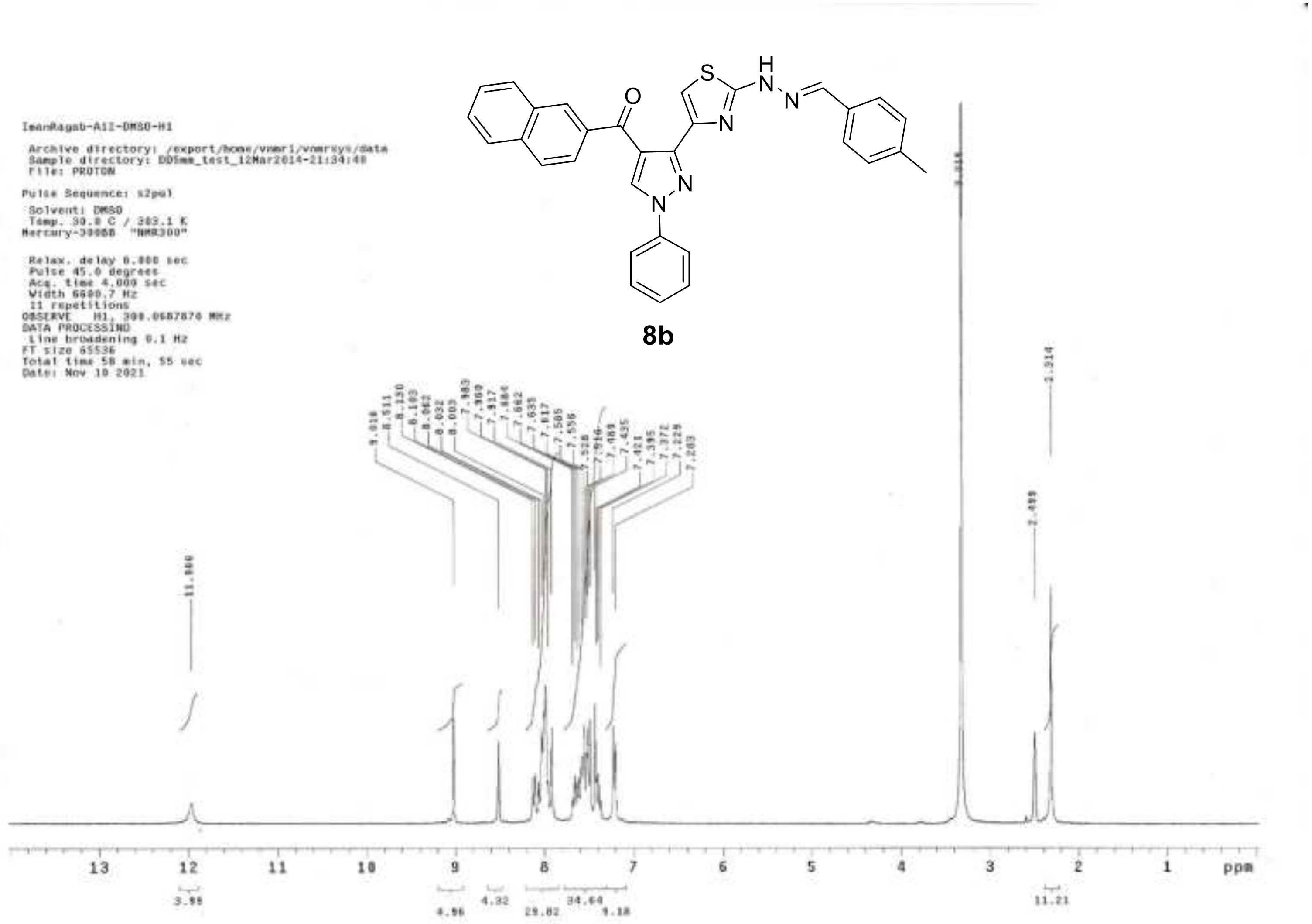

Figure S4: ^{1}H NMR spectrum of compound 8b

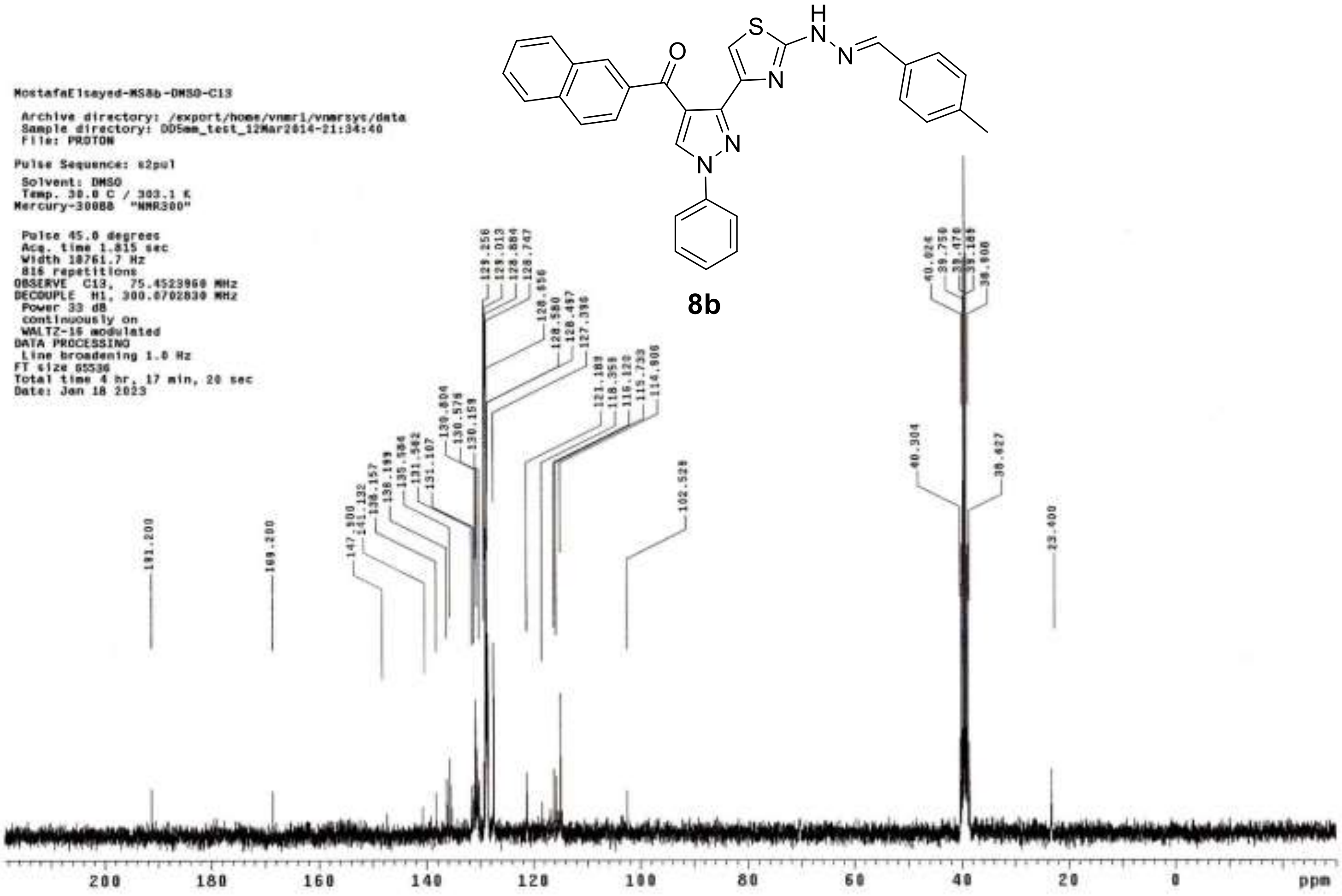

Figure S5: ^{13}C NMR spectrum of compound 8b

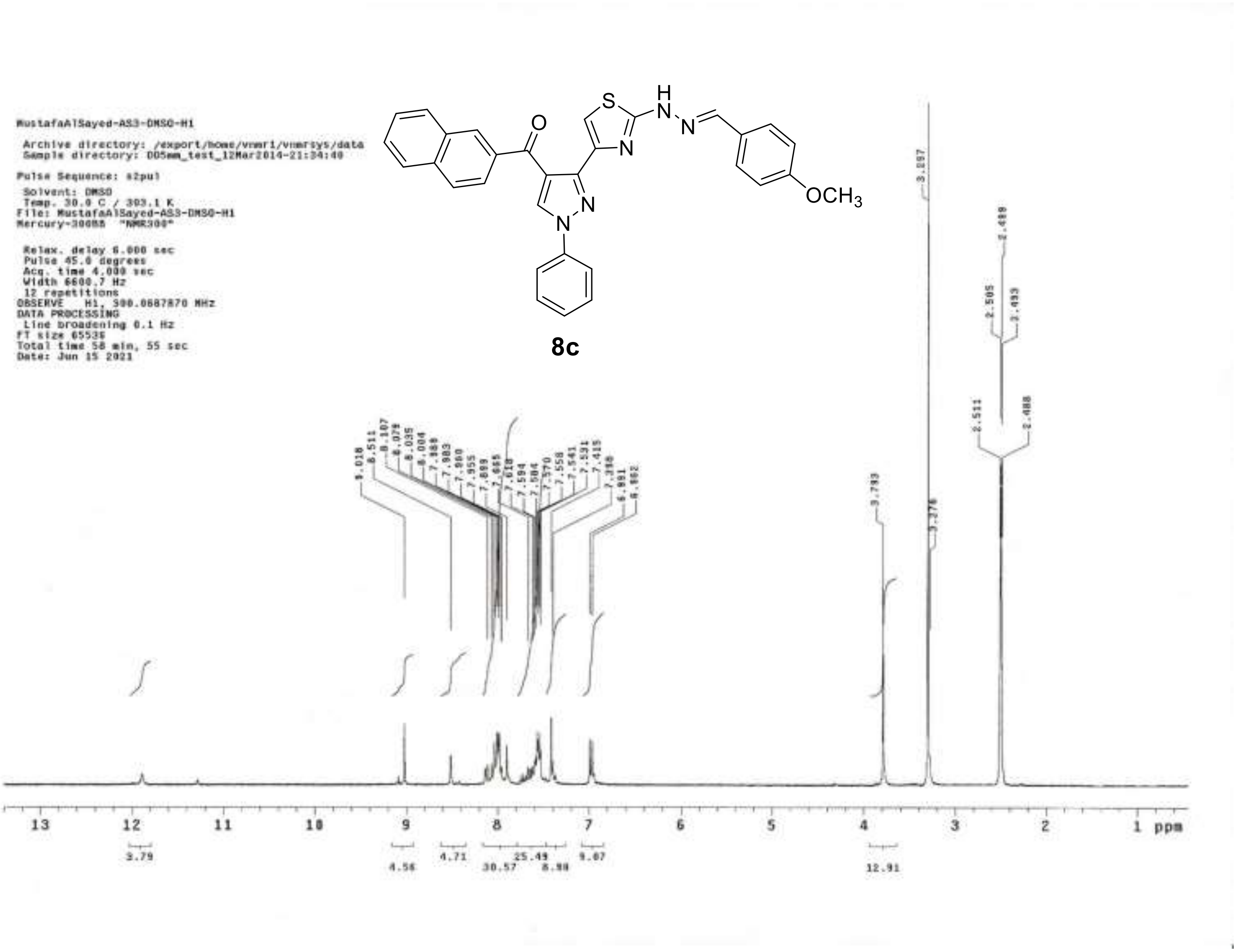

Figure S6: ^{1}H NMR spectrum of compound 8c

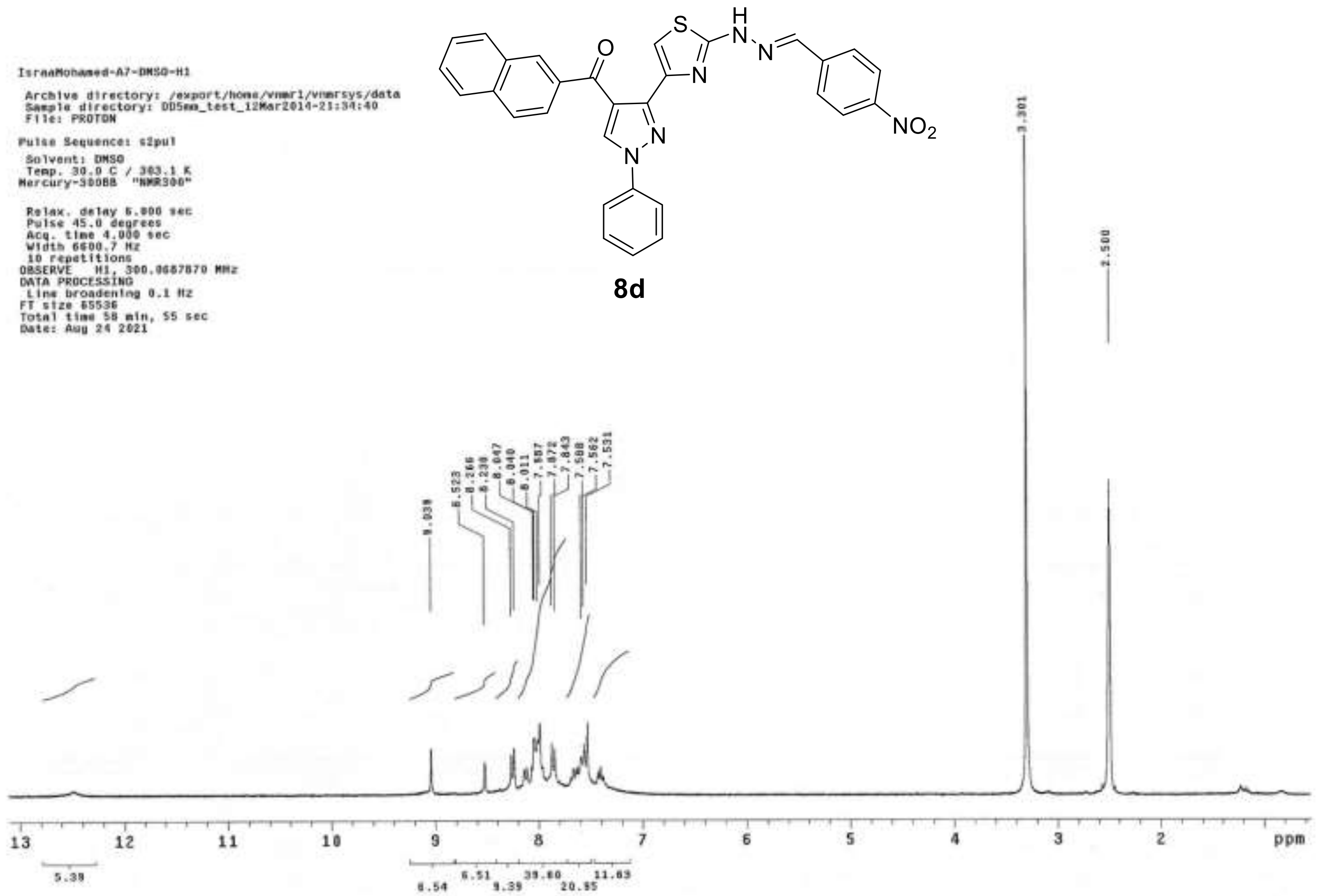

Figure S7: ^{1}H NMR spectrum of compound 8d

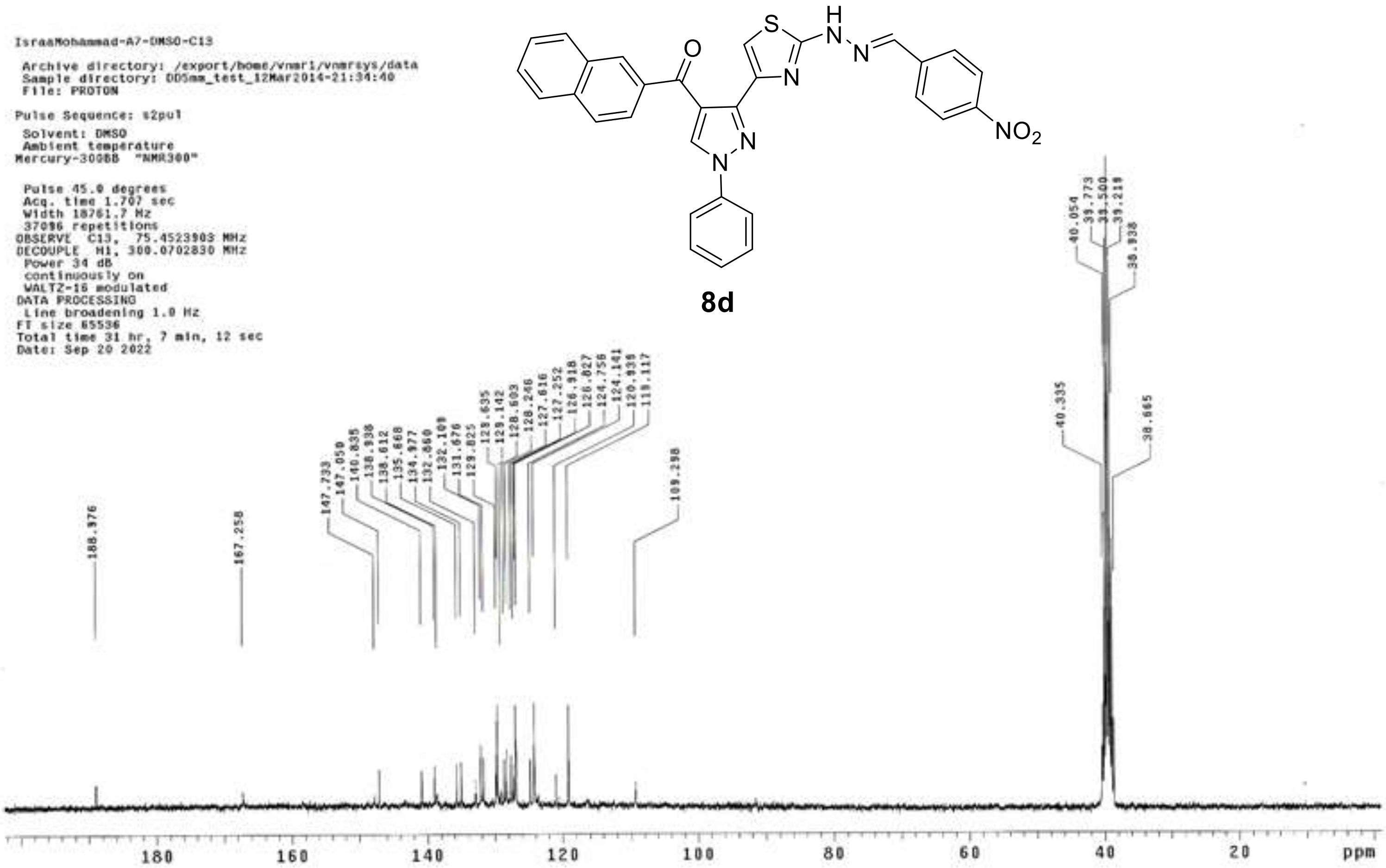

Figure S8: ^{13}C NMR spectrum of compound 8d

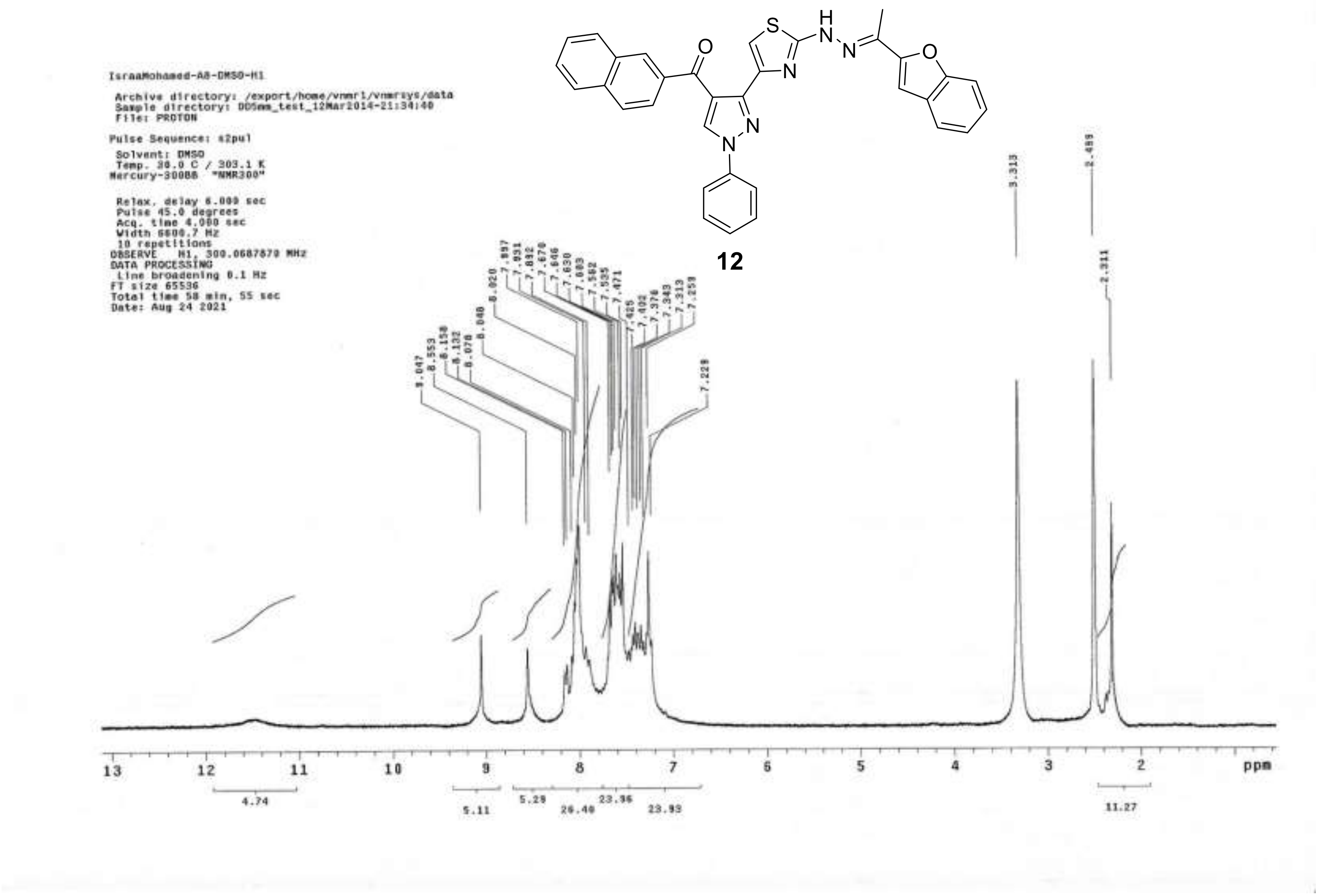

Figure S9: ^{1}H NMR spectrum of compound 12

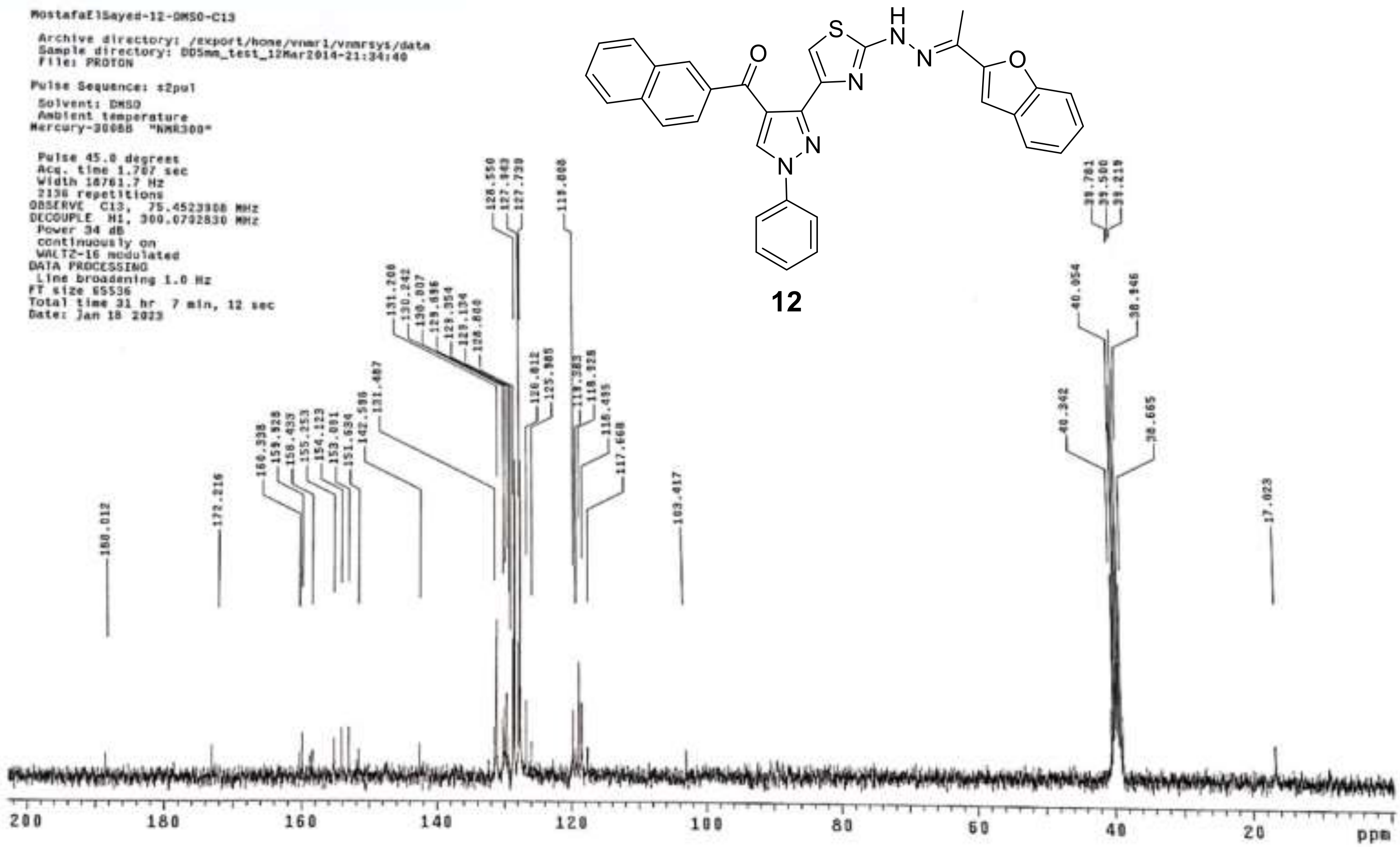

Figure S10: ^{13}CNMR spectrum of compound 12

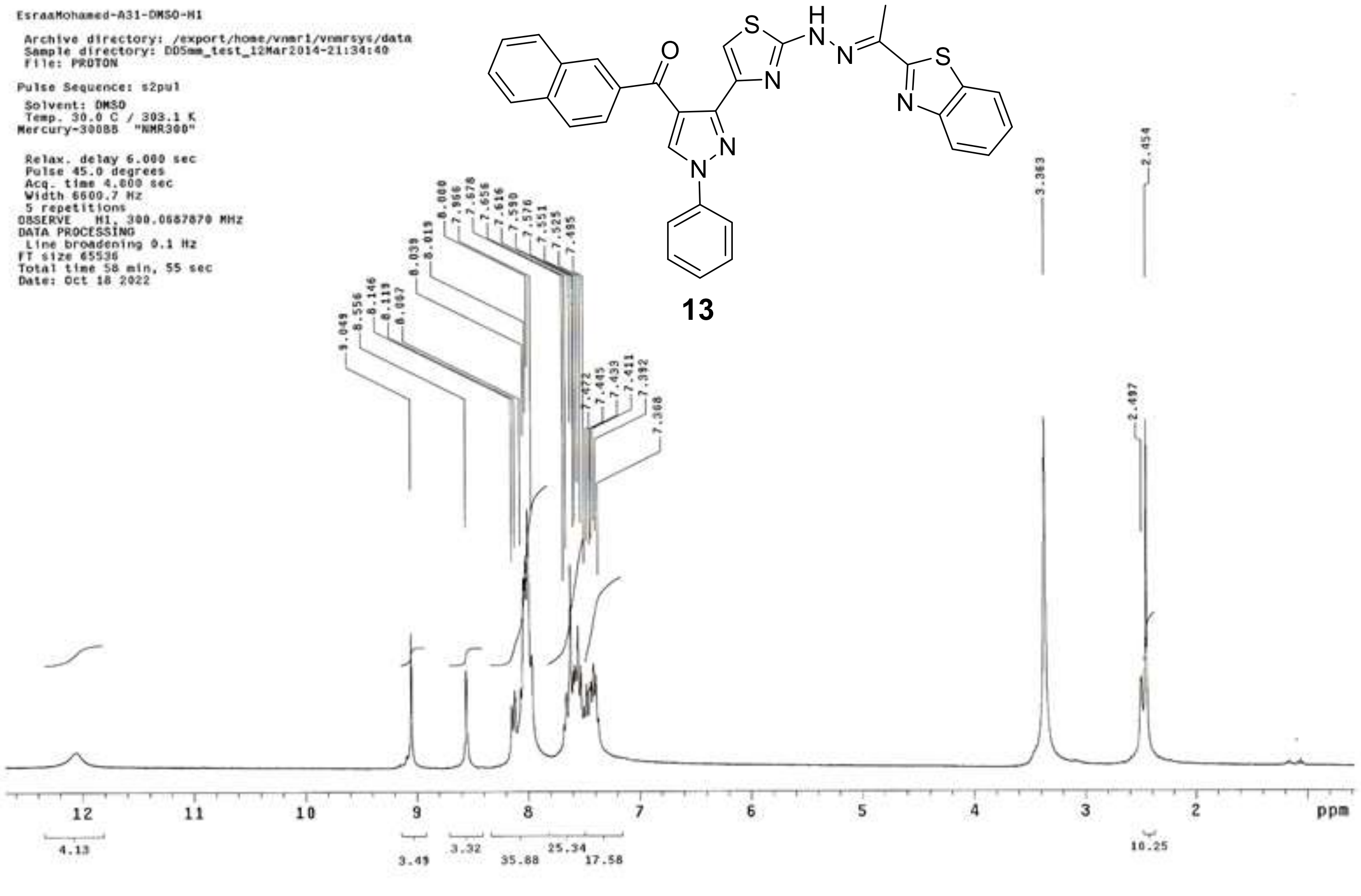

Figure S11: ^{1}H NMR spectrum of compound 13

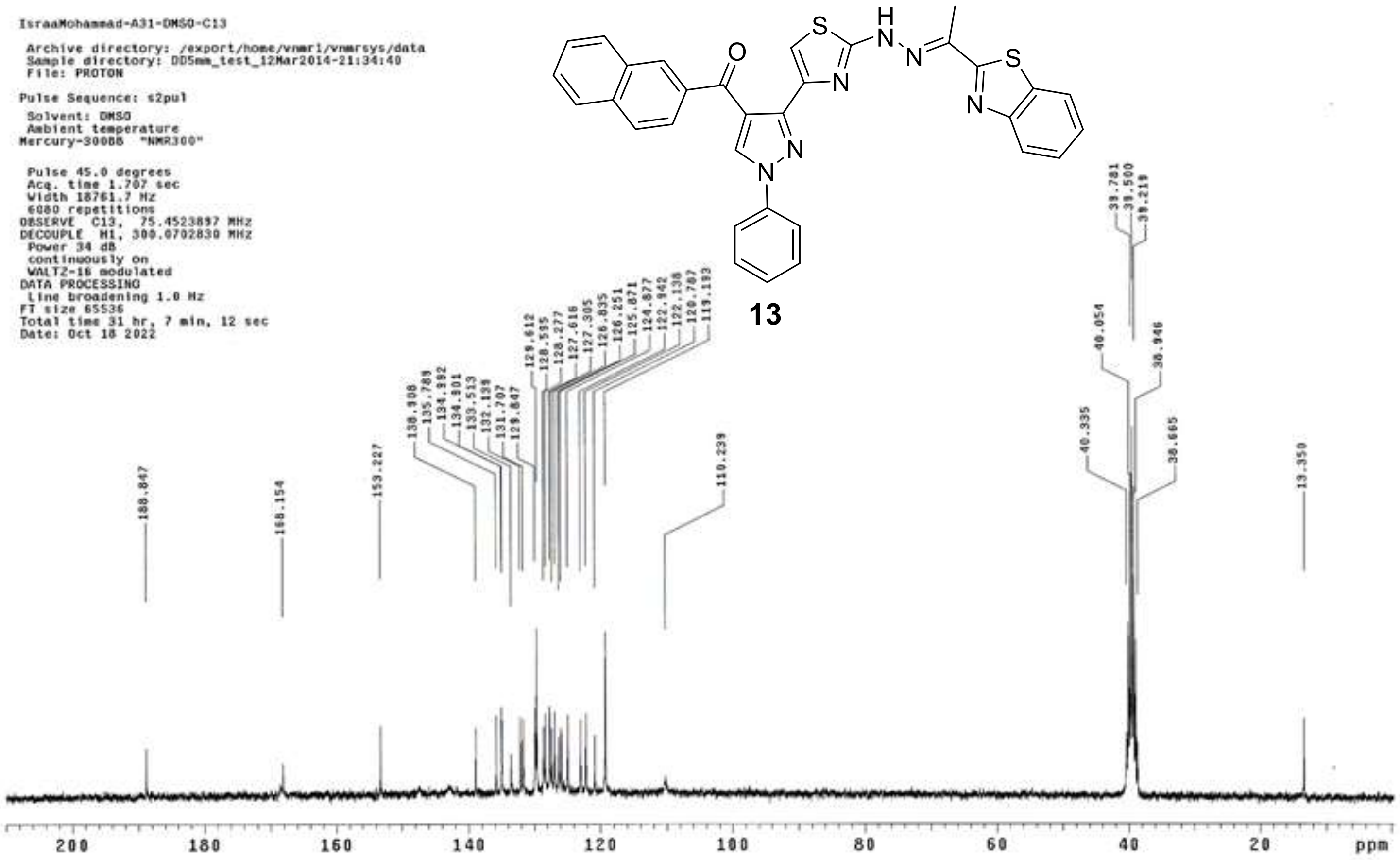

Figure S12: ^{13}C NMR spectrum of compound 13

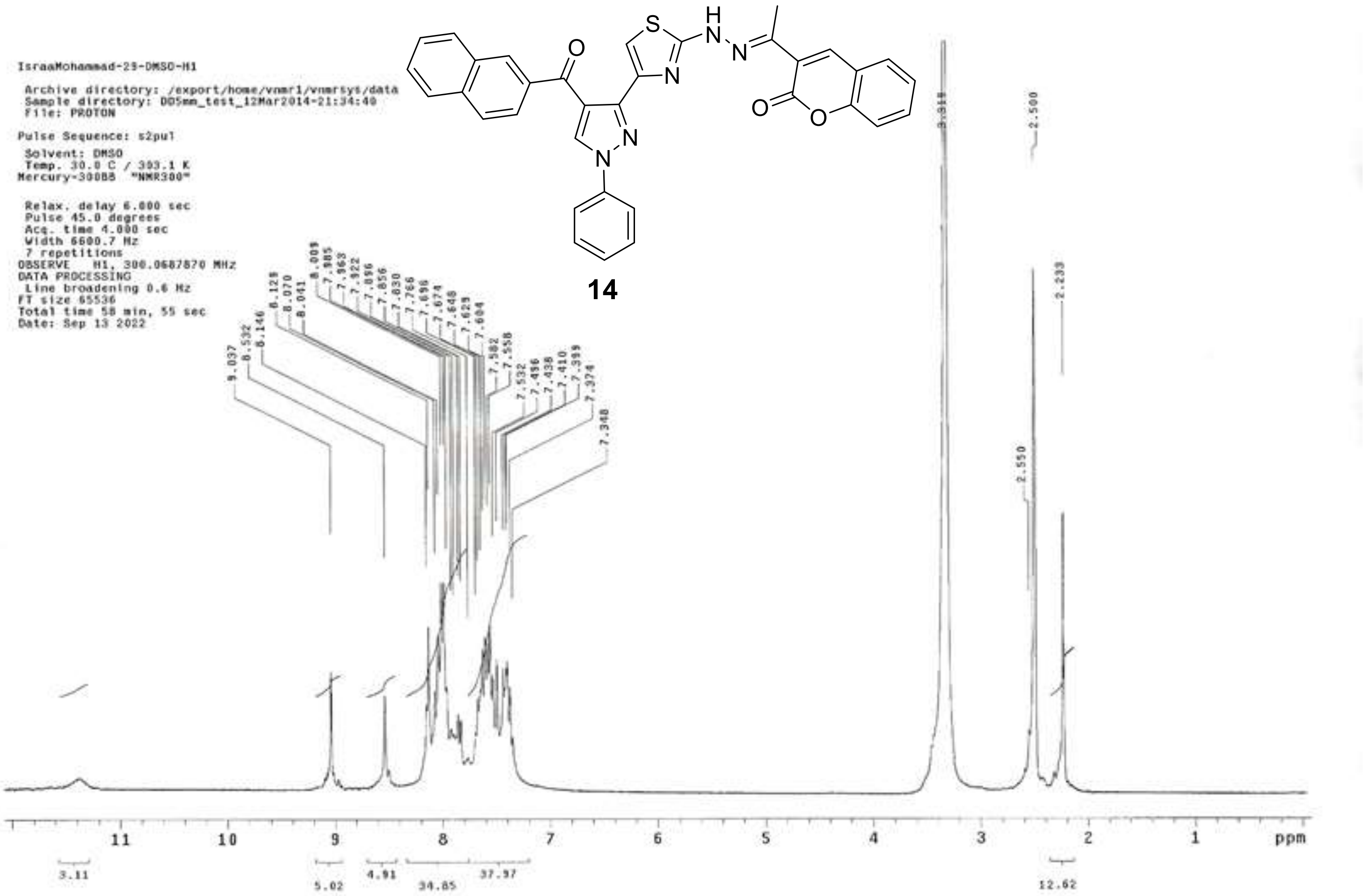

Figure S13: ^{1}H NMR spectrum of compound 14

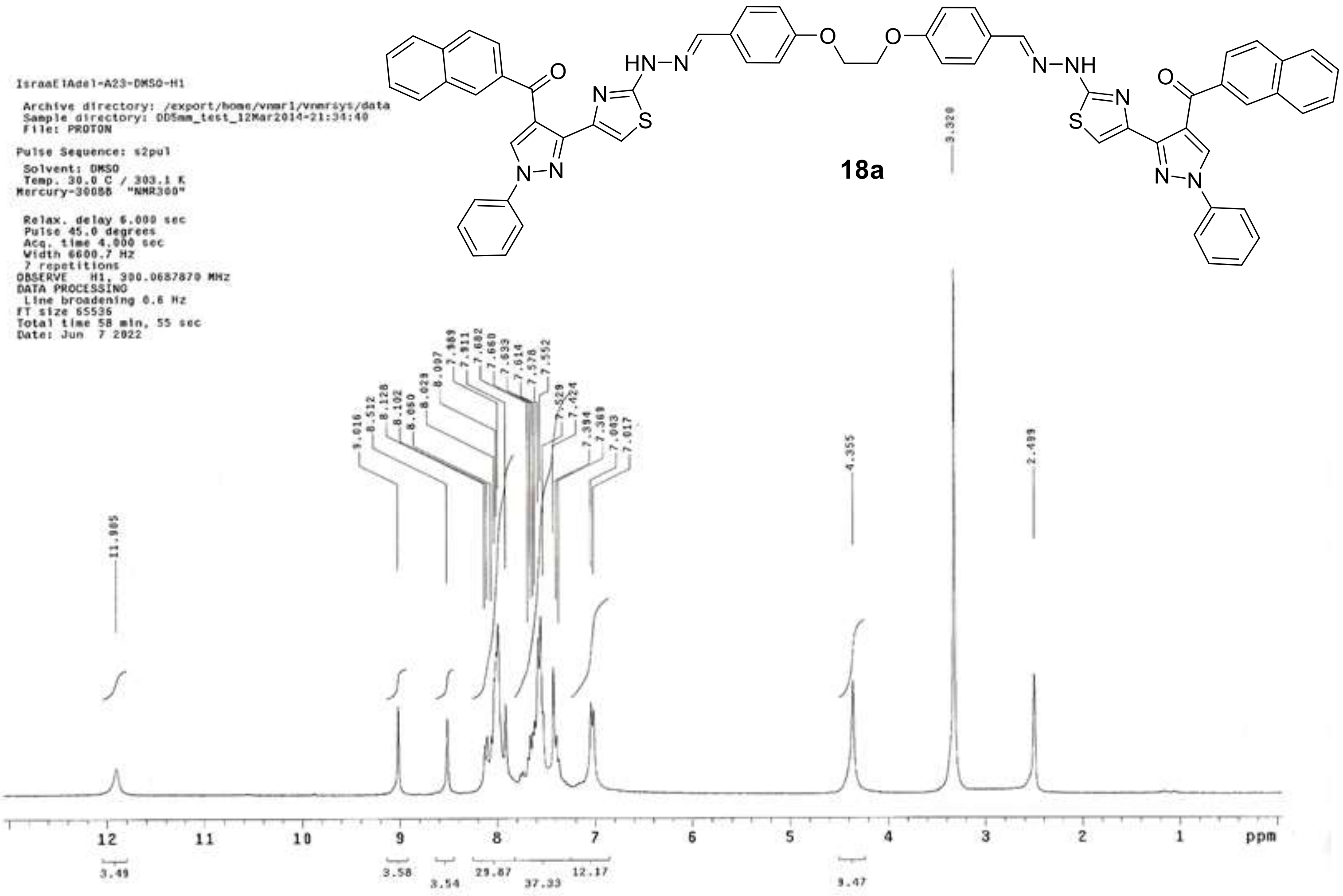

Figure S14: ^{1}H NMR spectrum of compound 18a

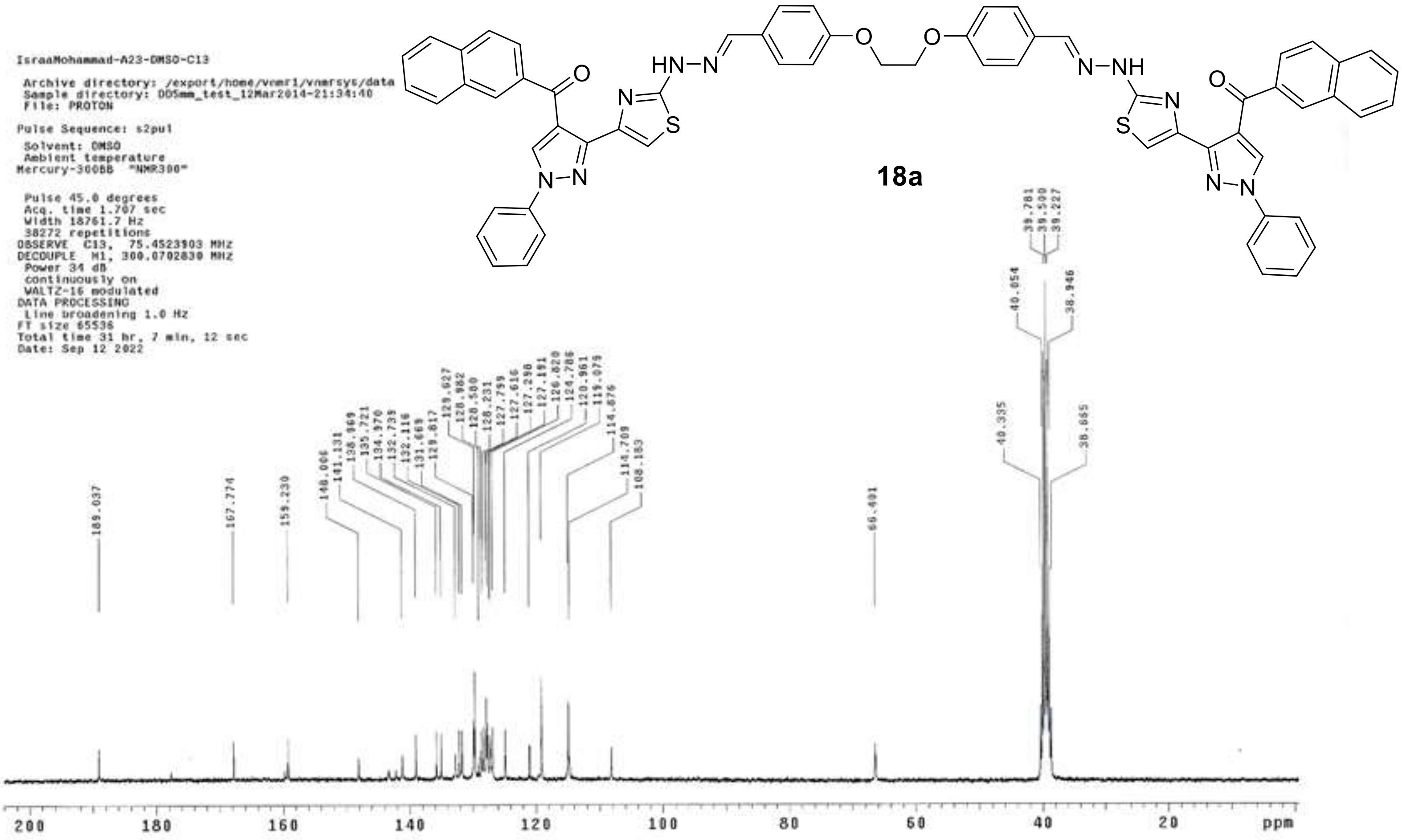

Figure S15: ^{13}C NMR spectrum of compound 18a

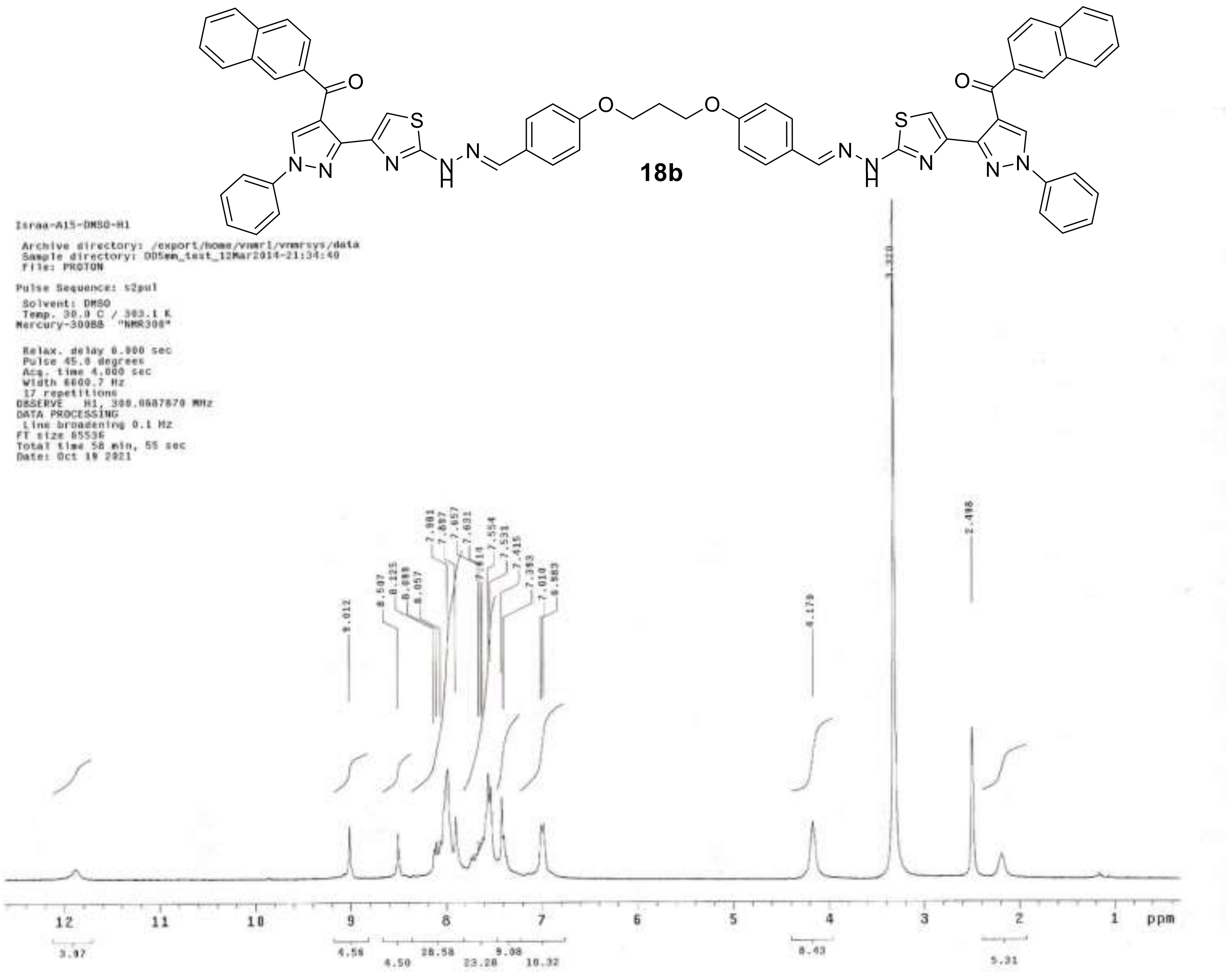

Figure S16: ^{1}H NMR spectrum of compound 18b

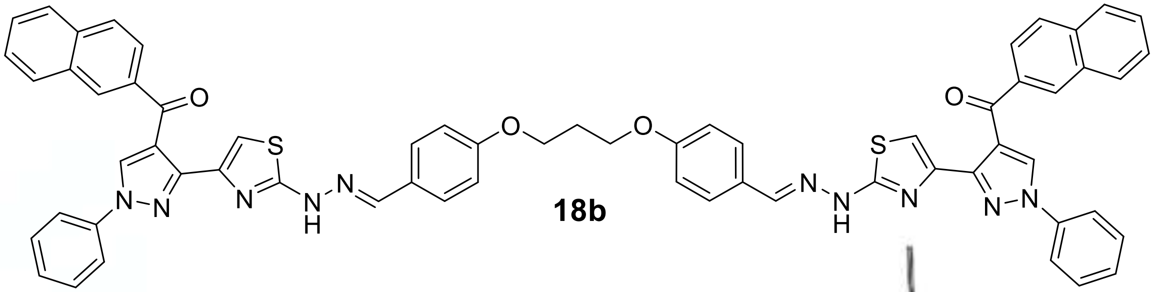

18b

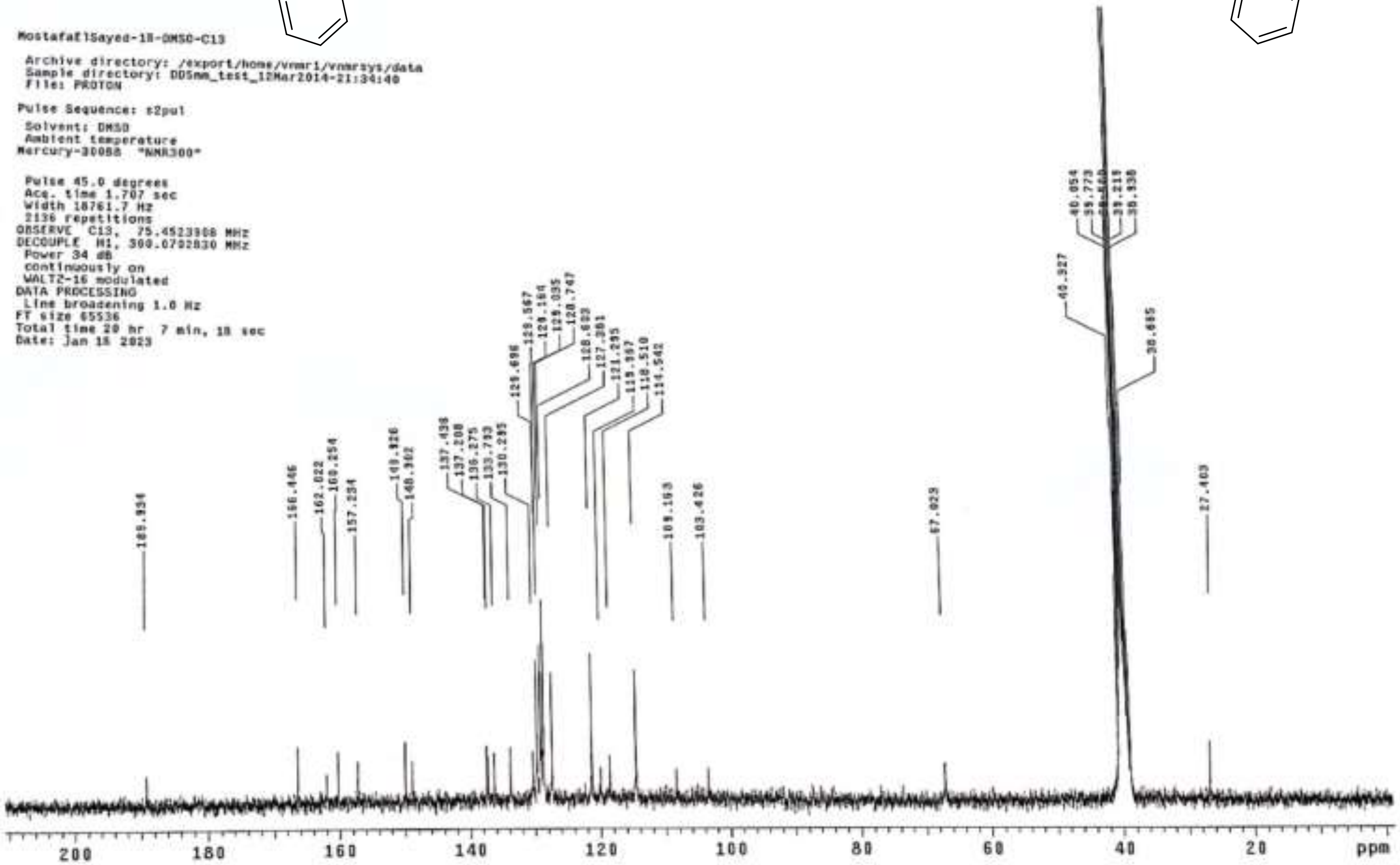

Figure S17: ^{13}C NMR spectrum of compound 18b

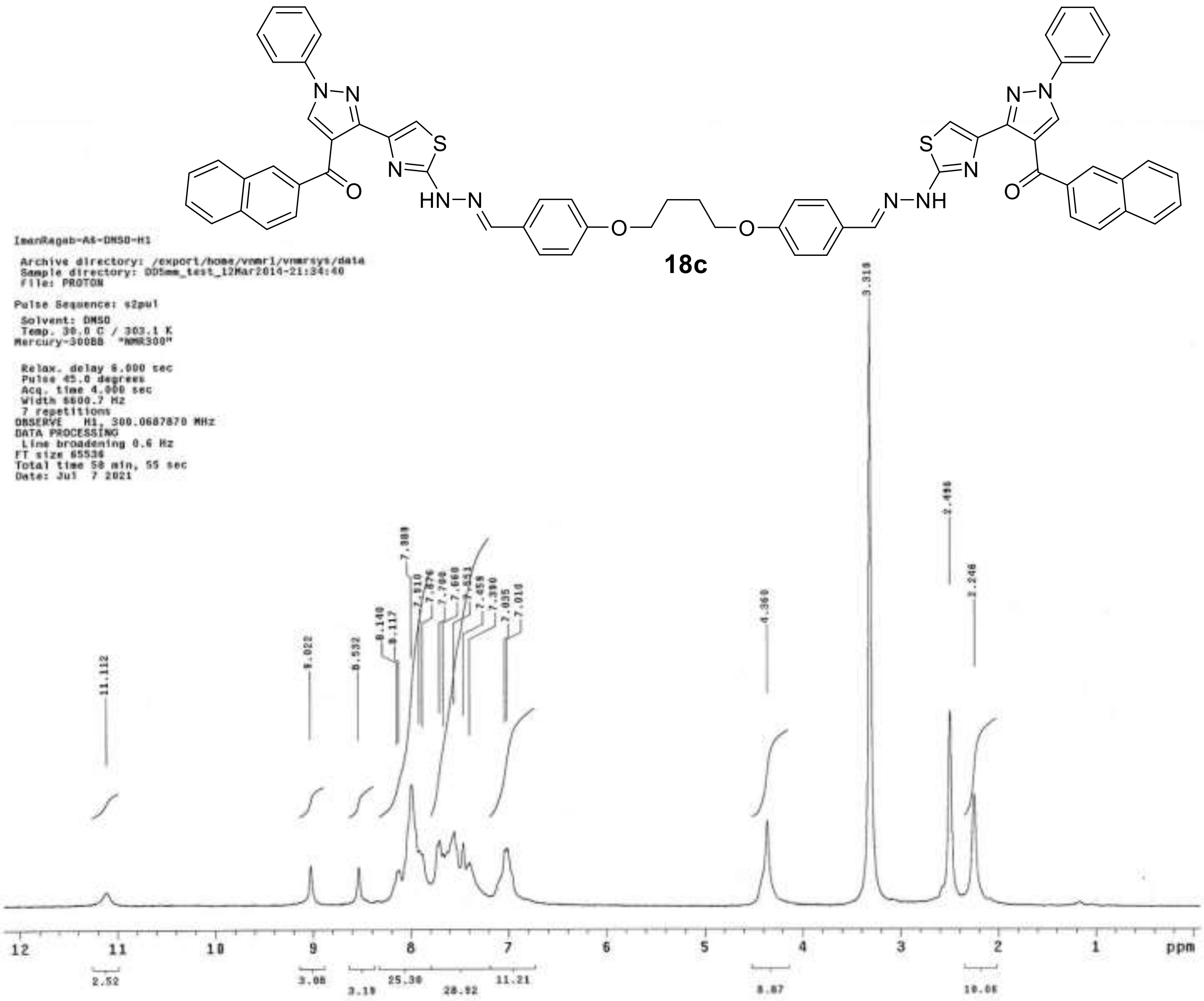

Figure S18: ^{1}H NMR spectrum of compound 18c

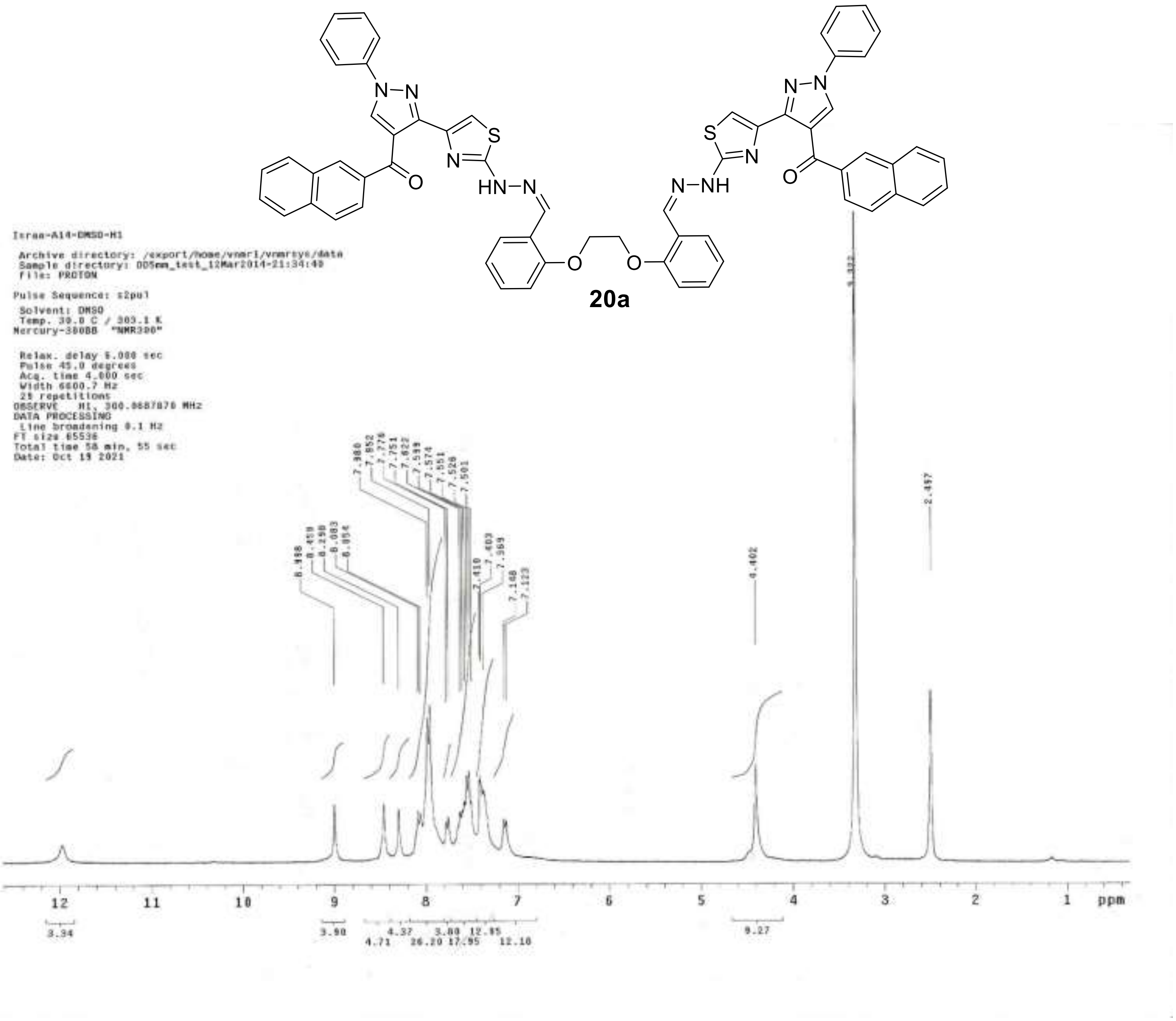

Figure S19: ^{1}HNMR spectrum of compound 20a

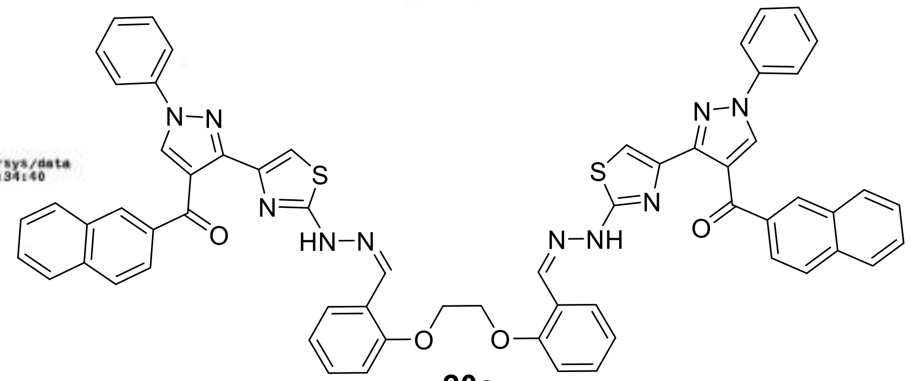

20a

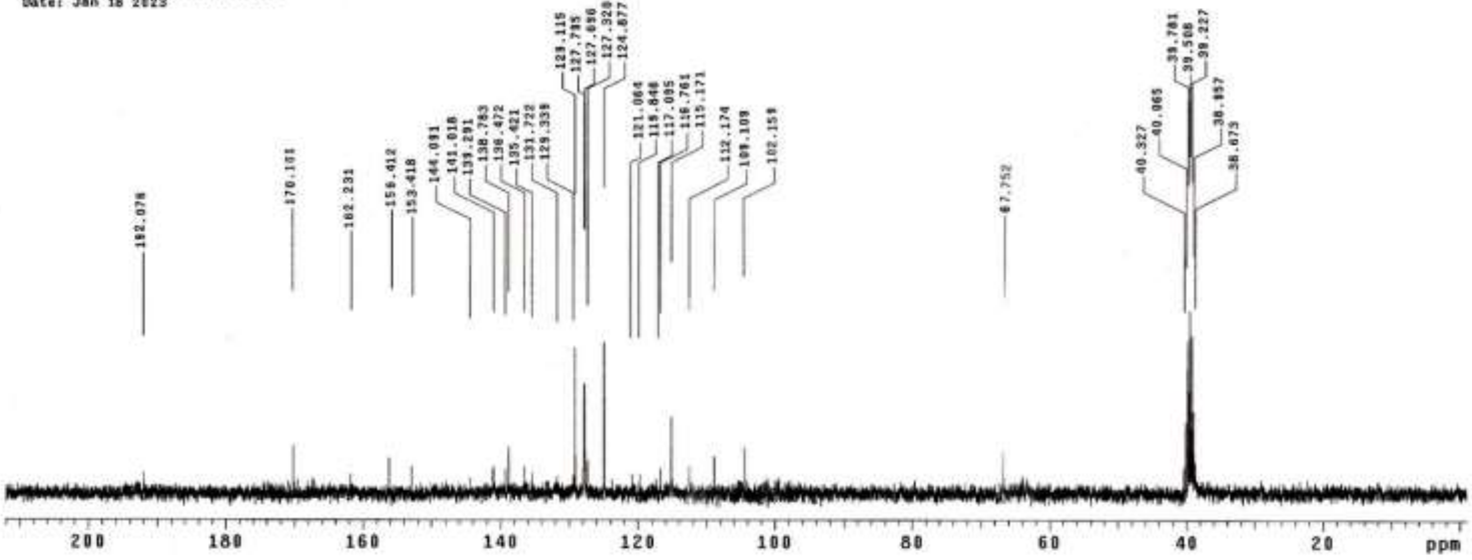

Figure S20: ^{13}C NMR spectrum of compound 20a

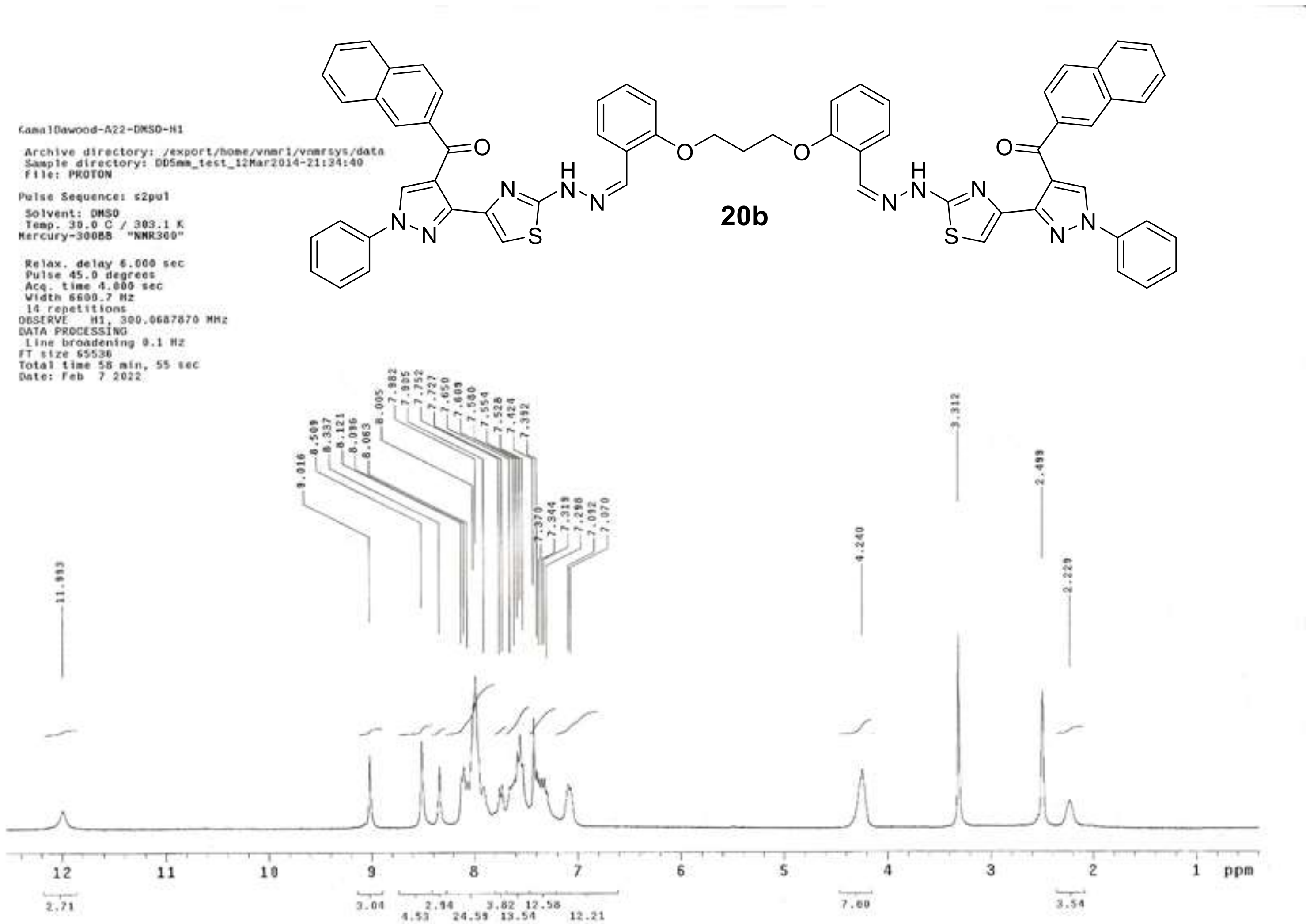

Figure S21: ^{1}H NMR spectrum of compound 20b

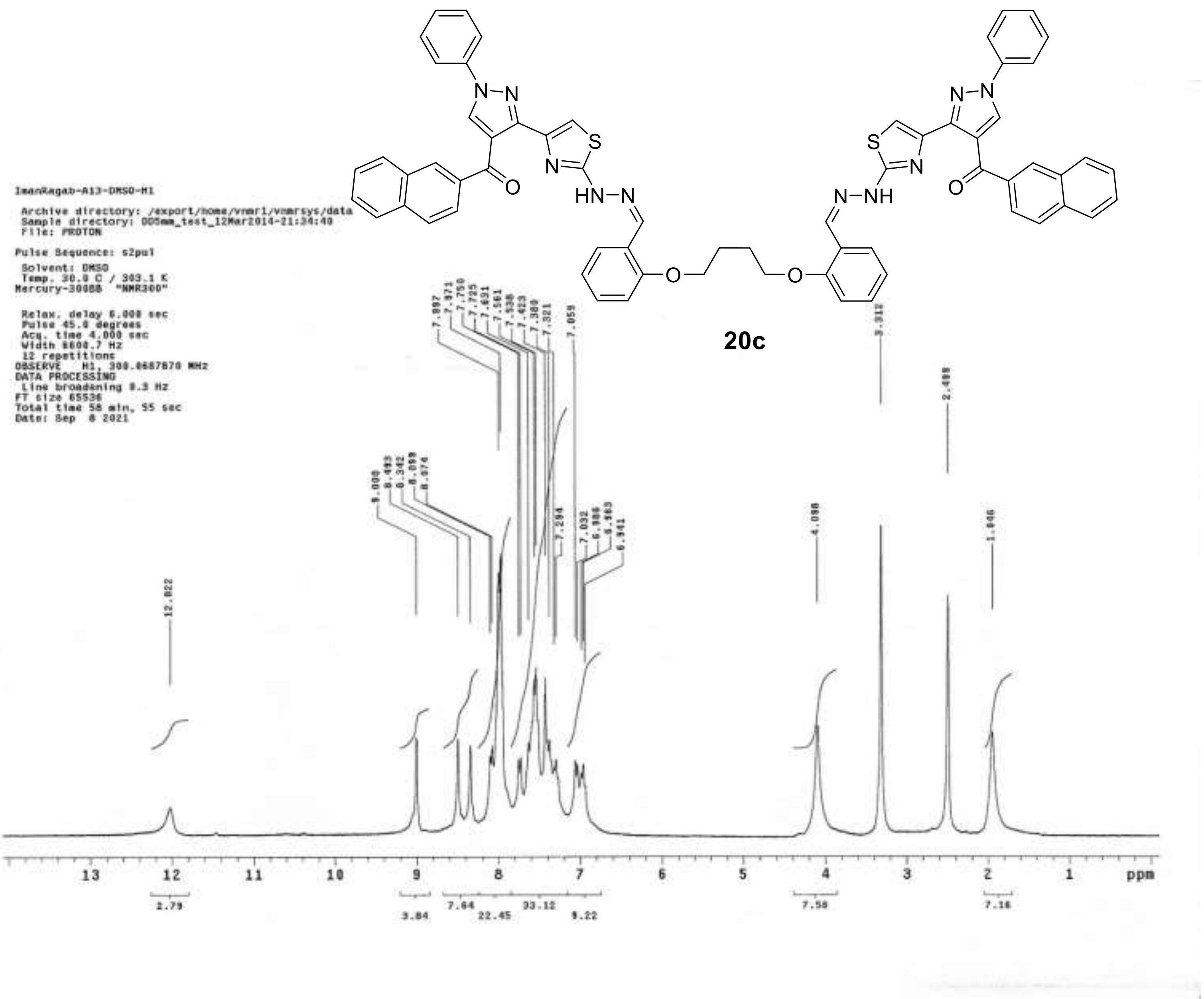

Figure S22: ^{1}H NMR spectrum of compound 20c

Some selected IR and Mass spectra

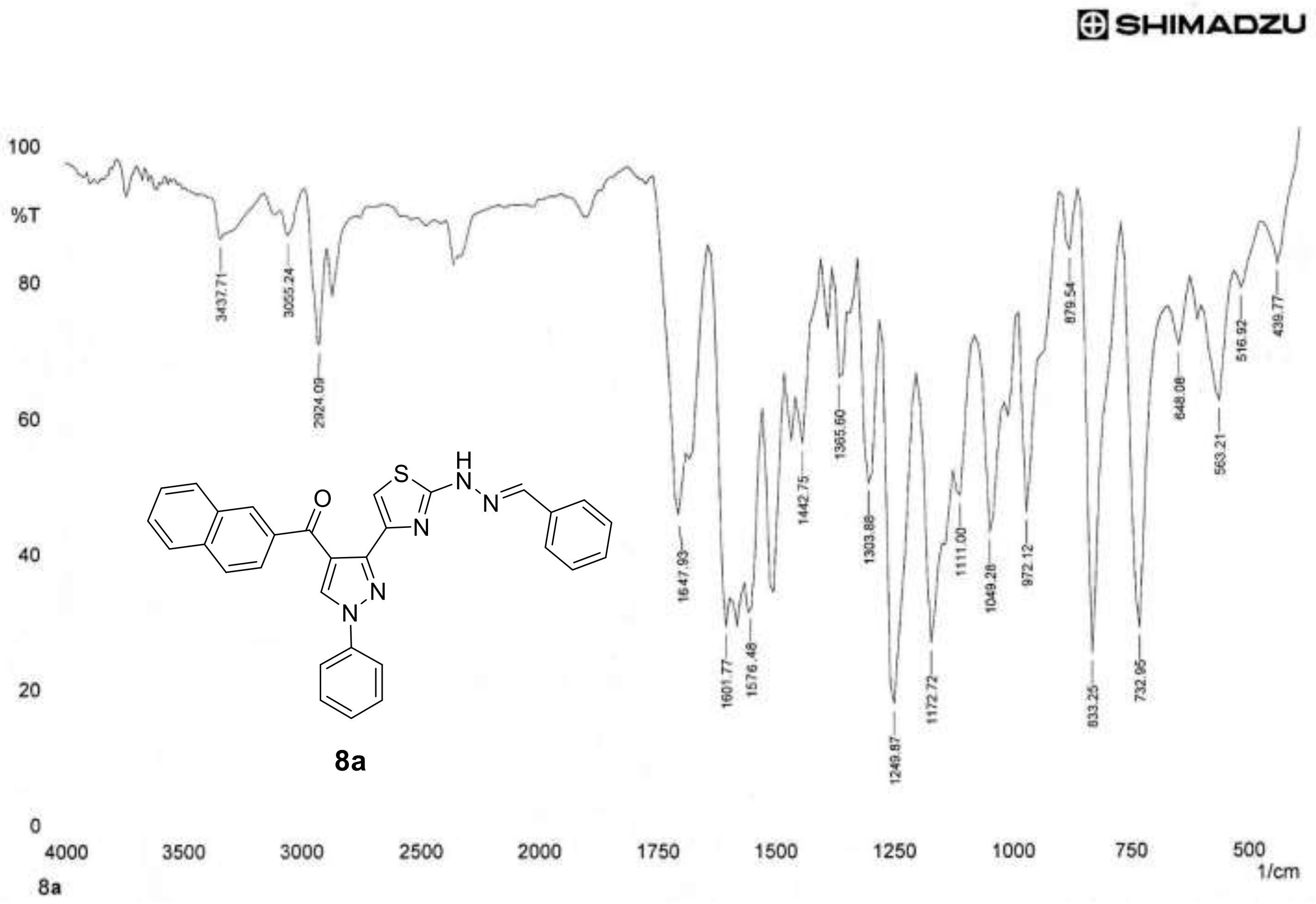

Figure S23: IR spectrum of compound 8a

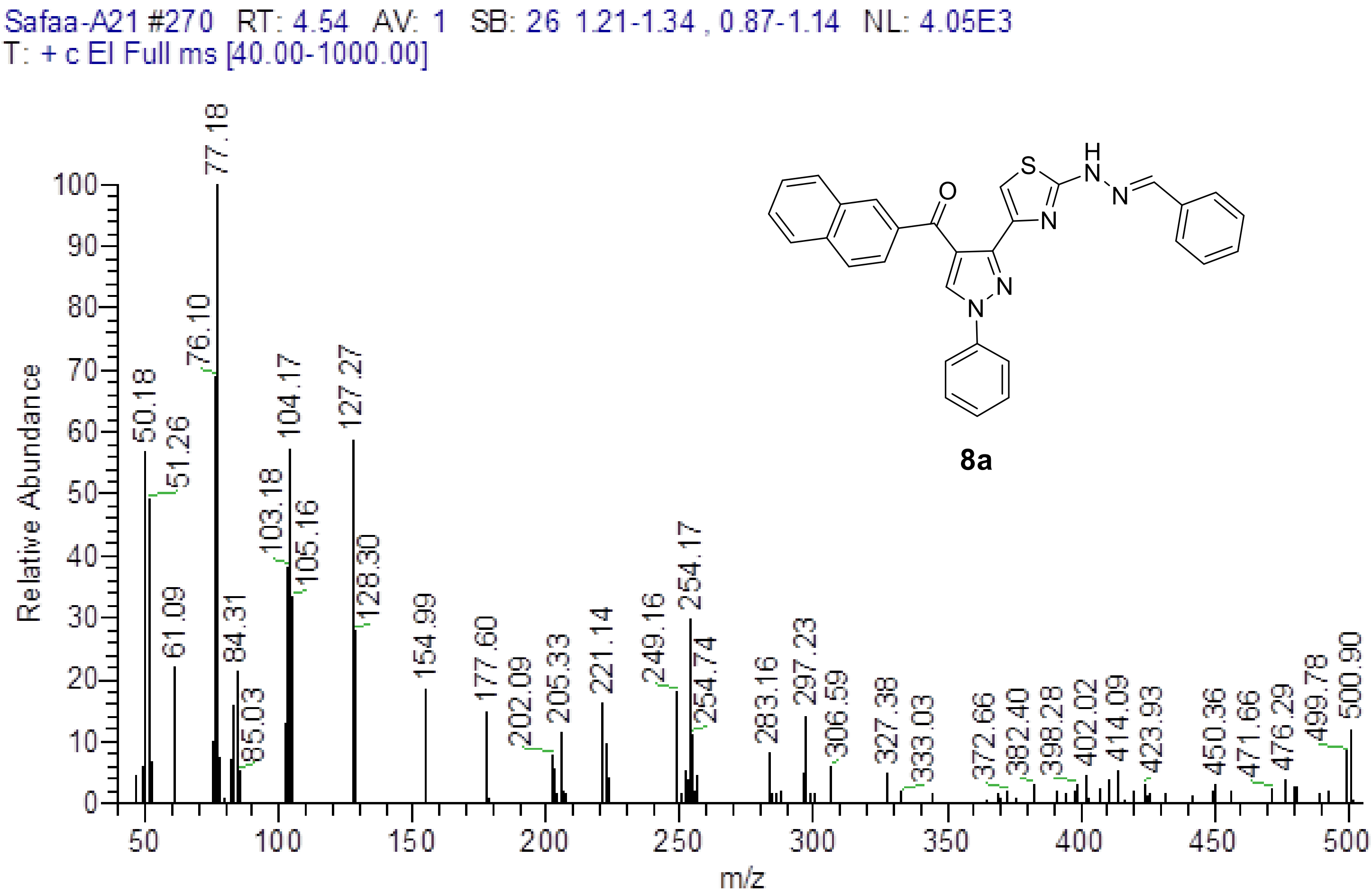

Figure S24: Mass spectrum of compound 8a

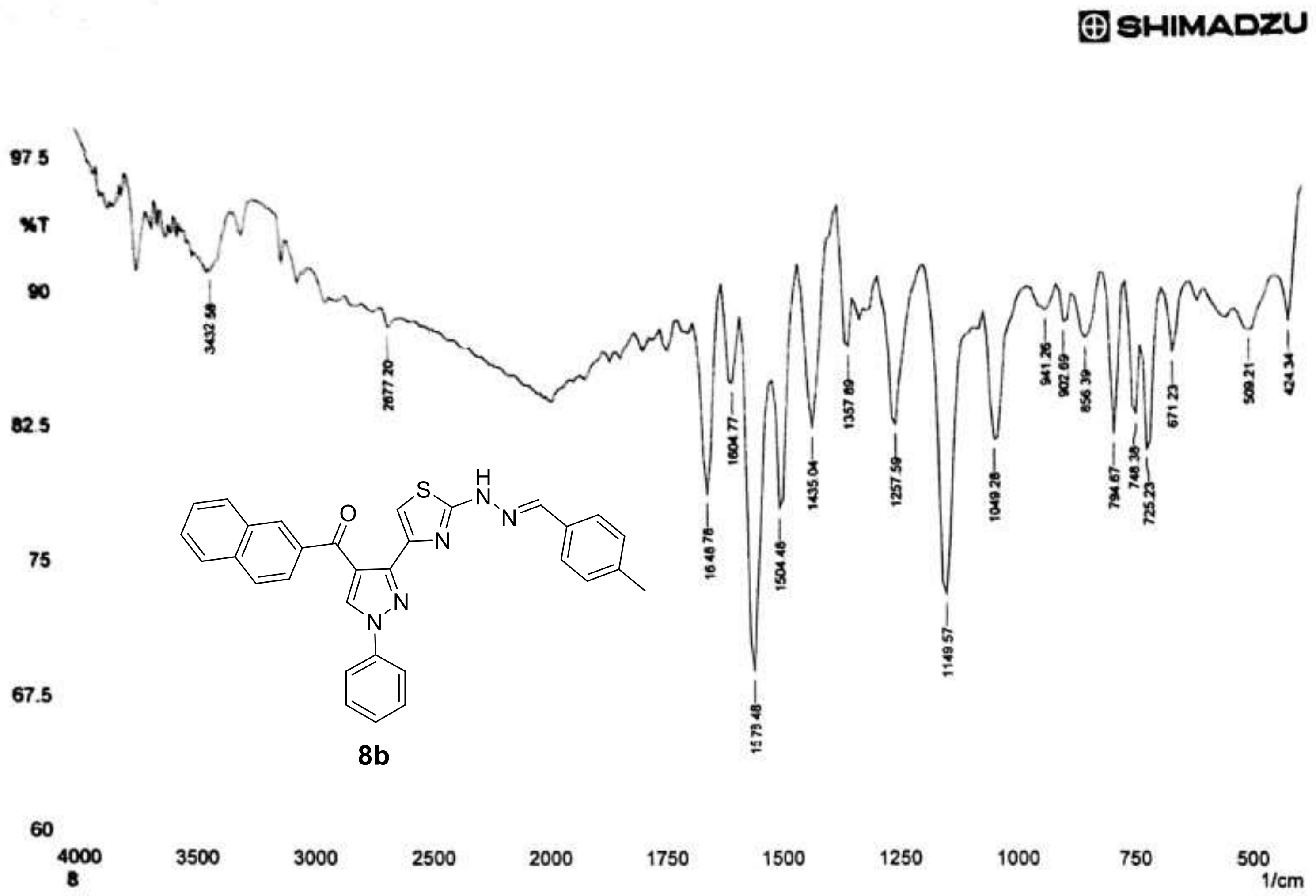

Figure S25: IR spectrum of compound 8b

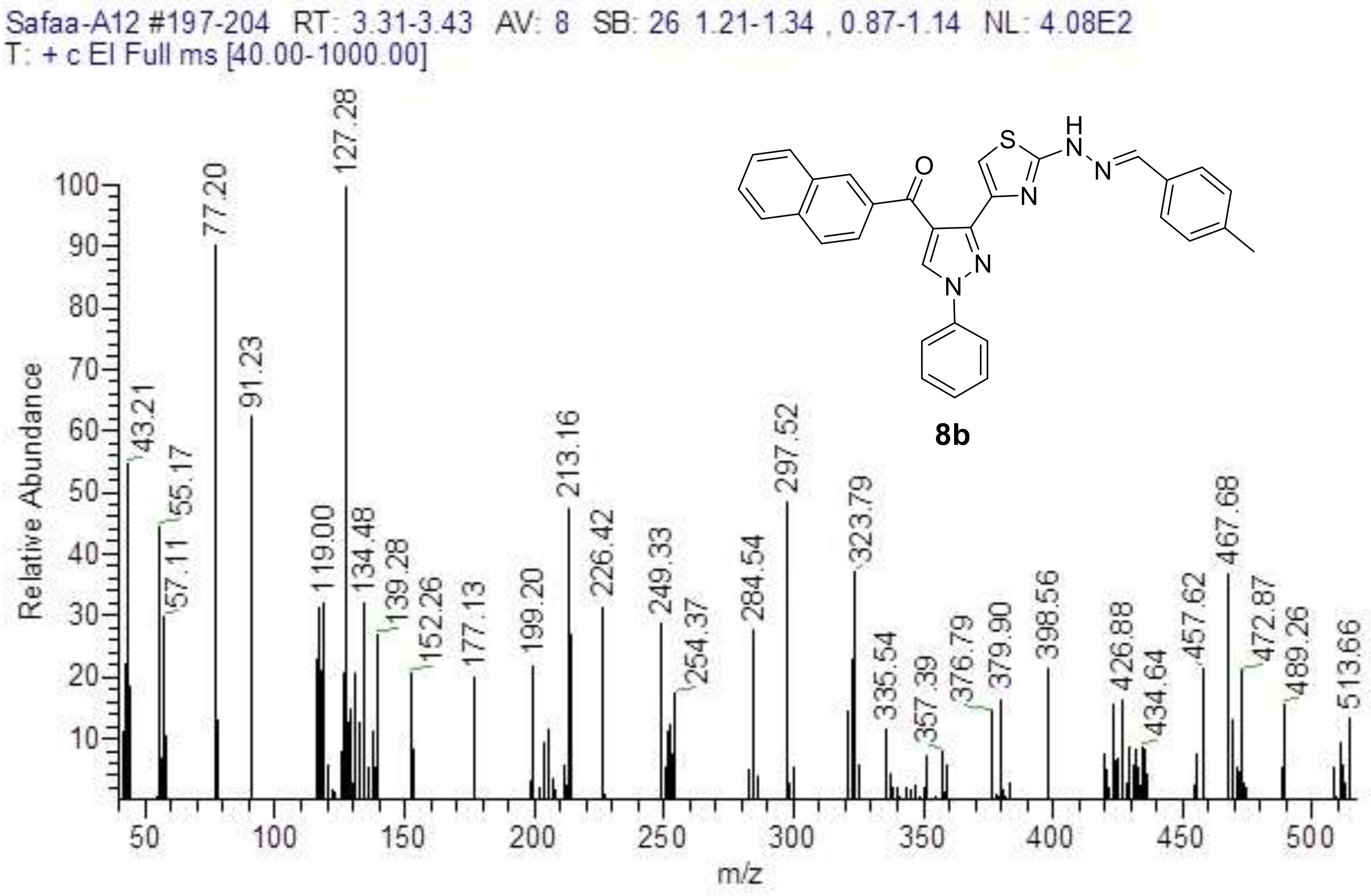

Figure S26: Mass spectrum of compound 8b

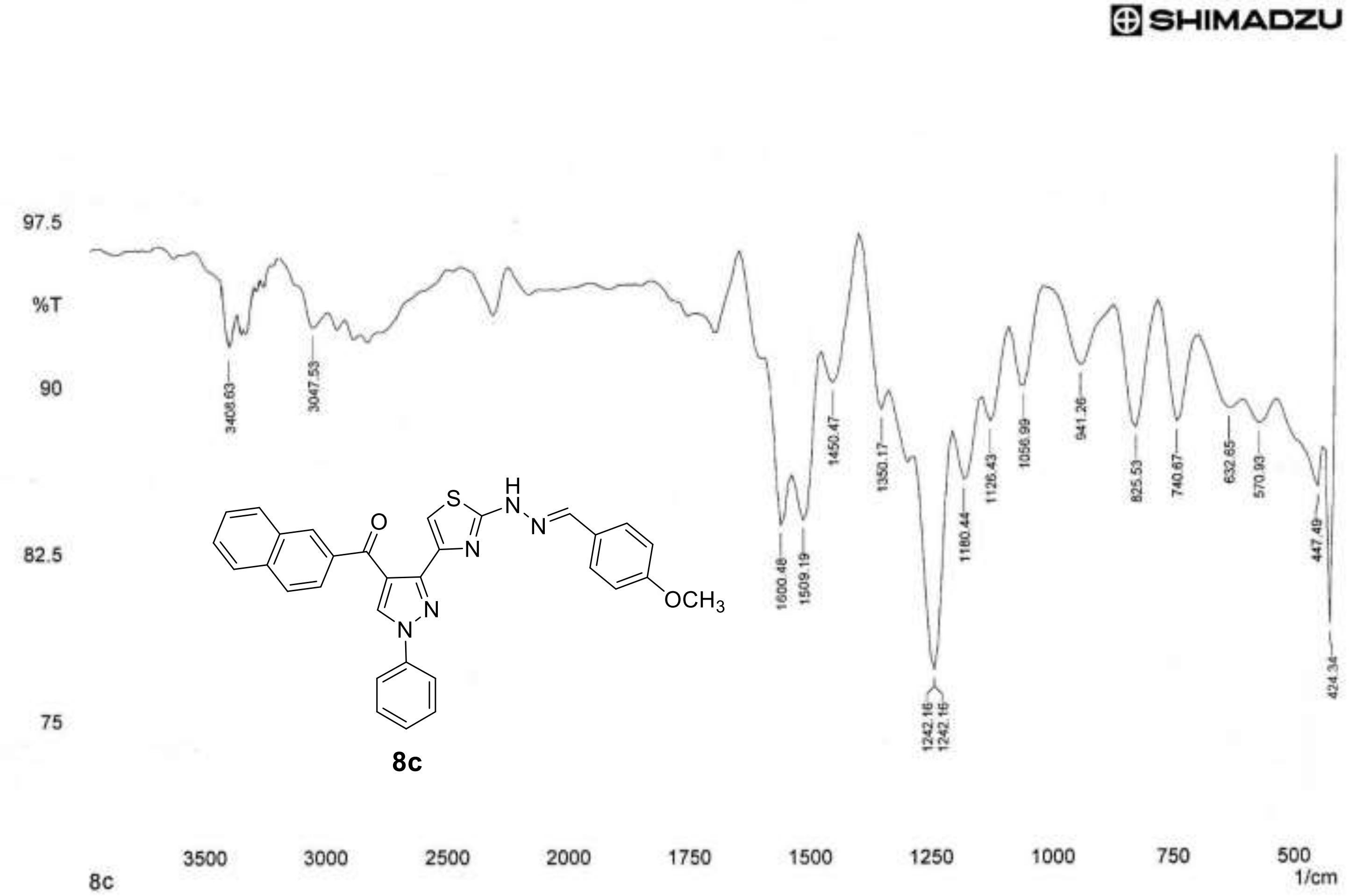

8c

Figure S27: IR spectrum of compound 8c

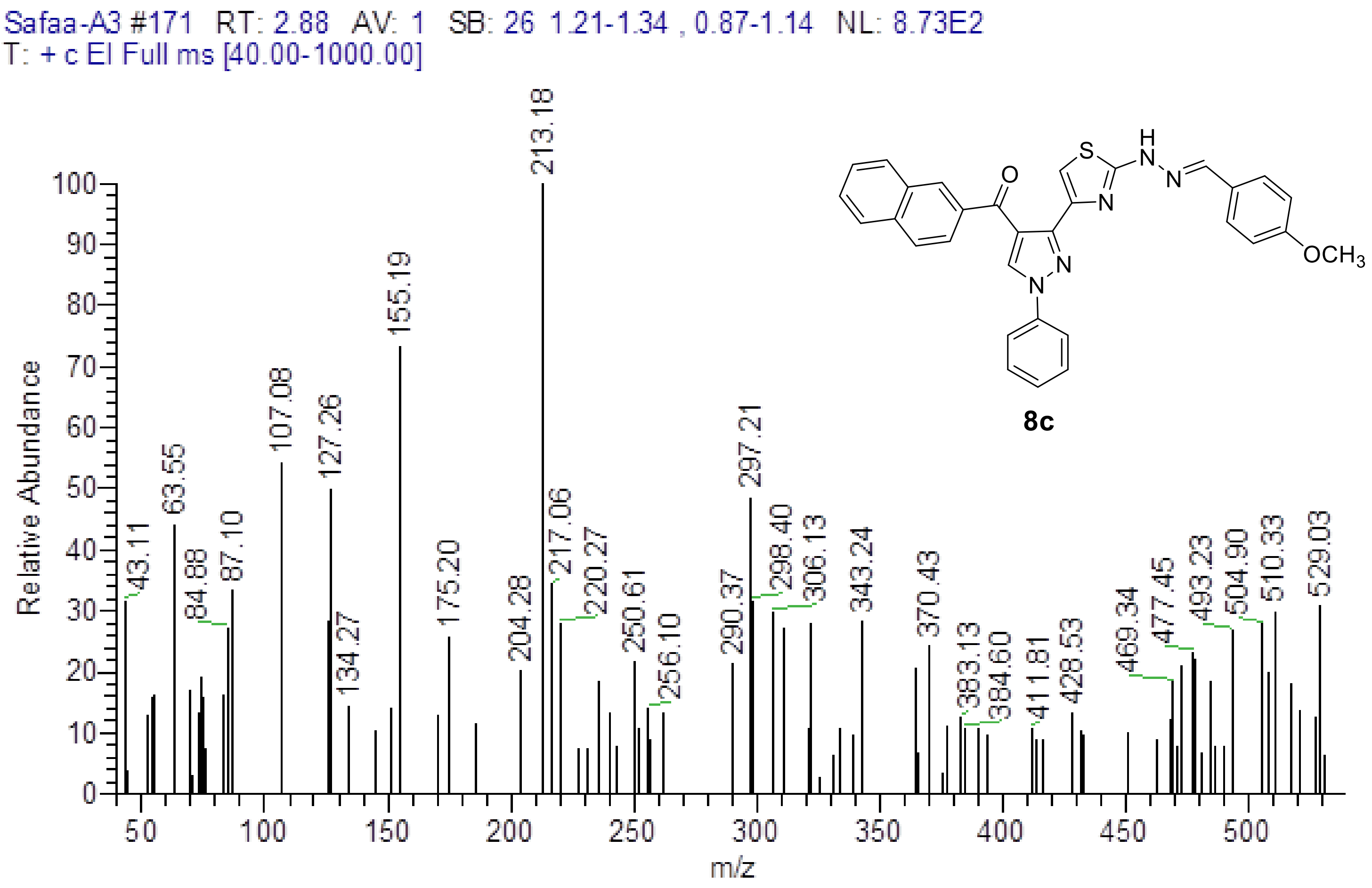

Figure S28: Mass spectrum of compound 8c

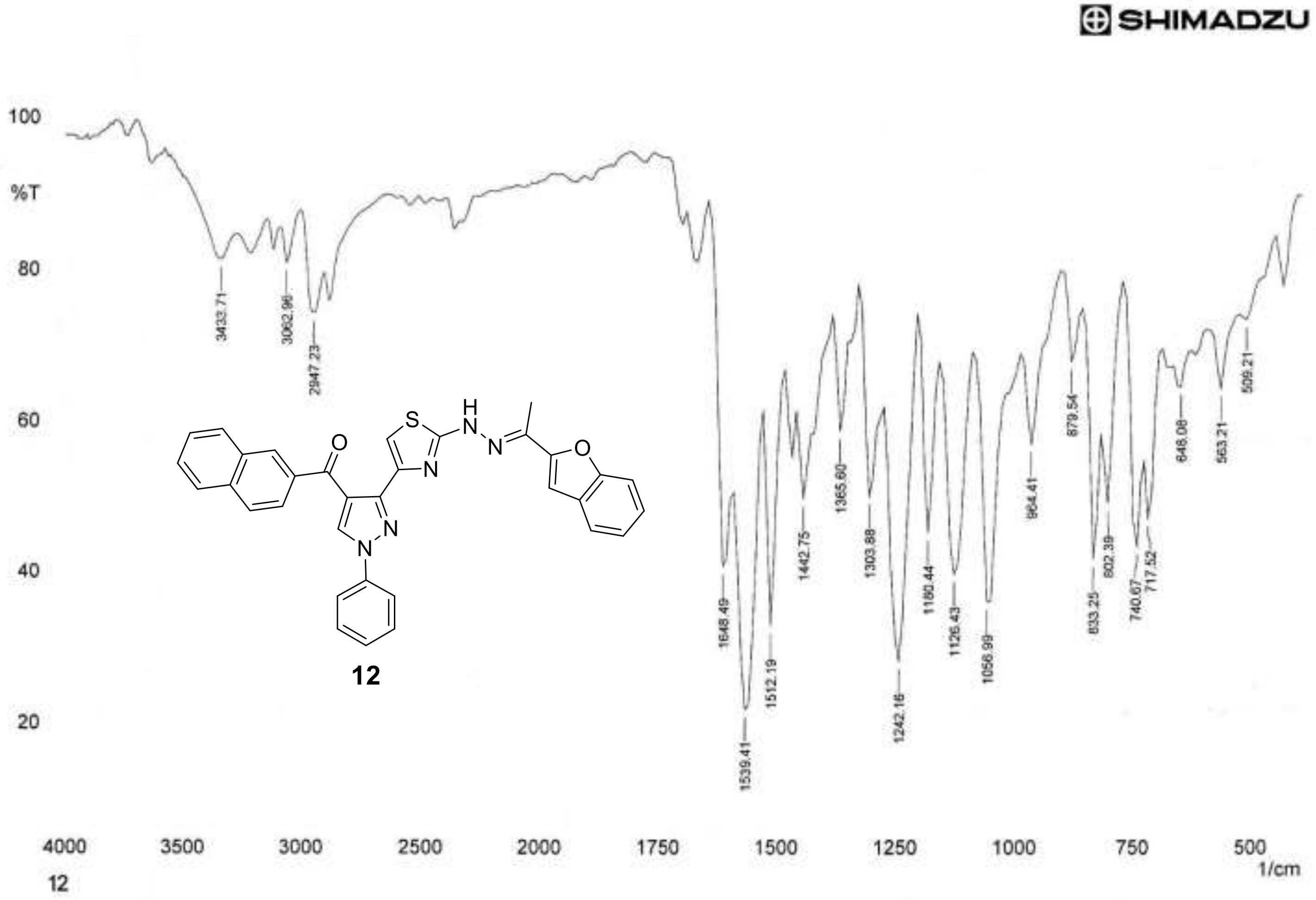

Figure S29: IR spectrum of compound 12

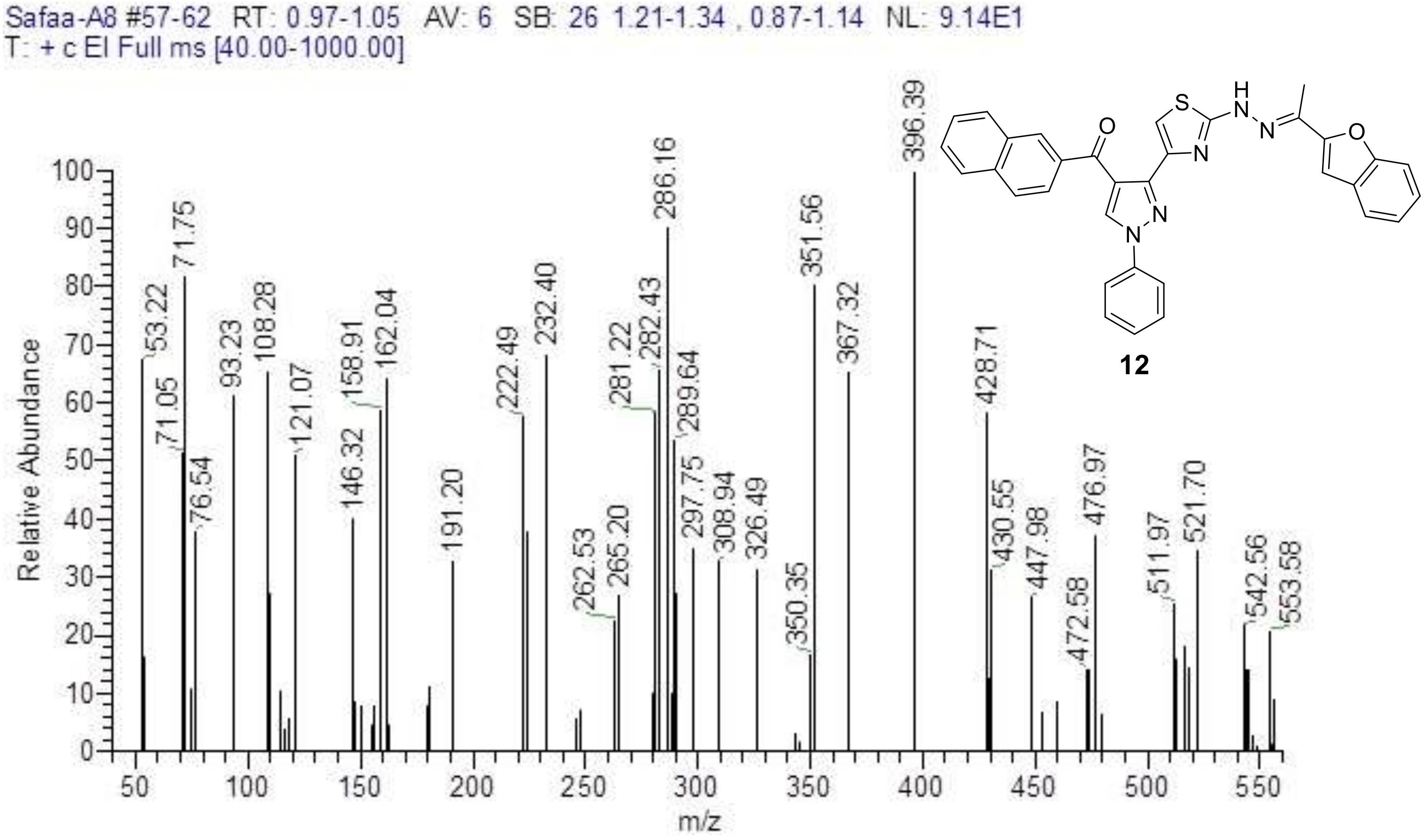

Figure S30: Mass spectrum of compound 12

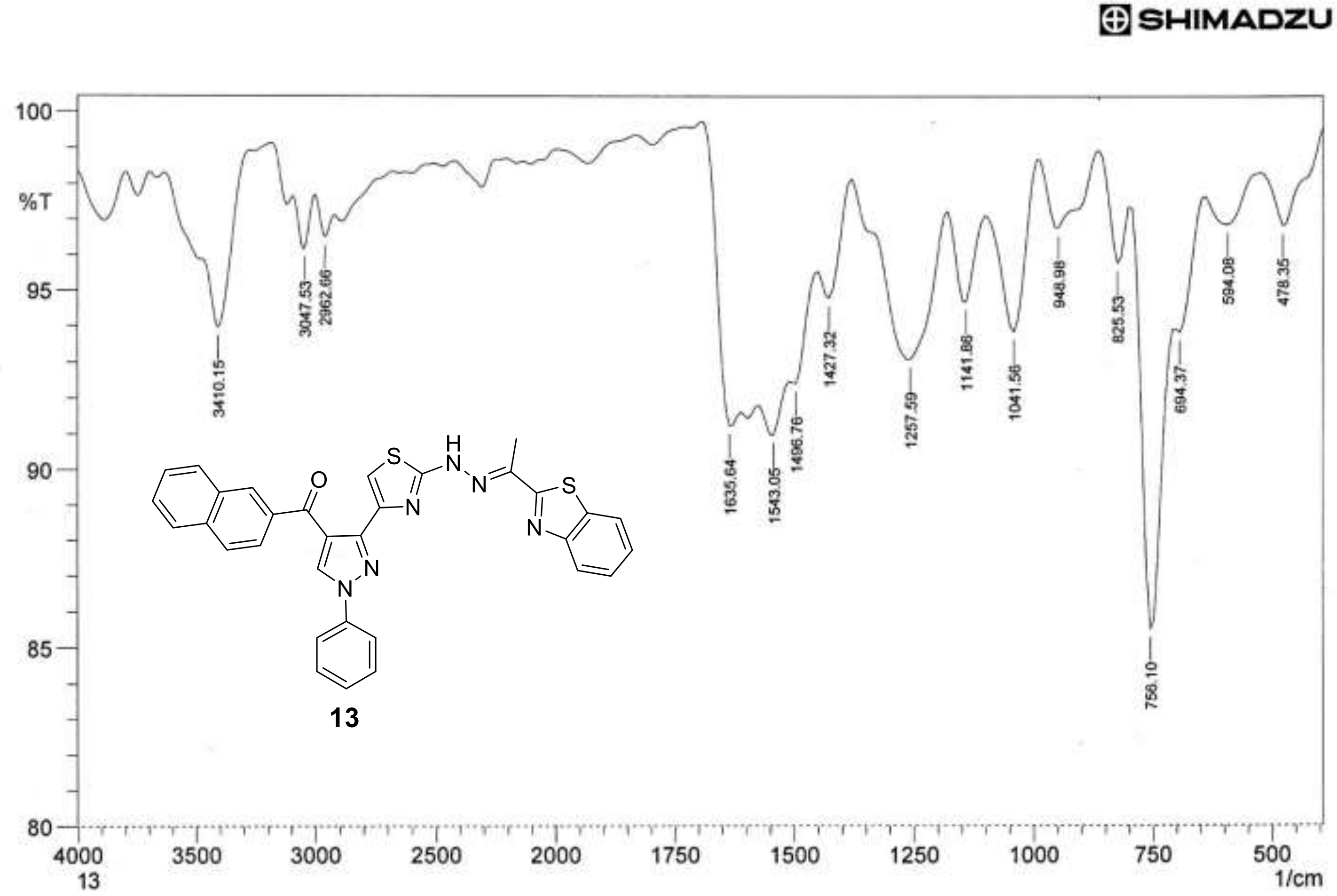

Figure S31: IR spectrum of compound 13

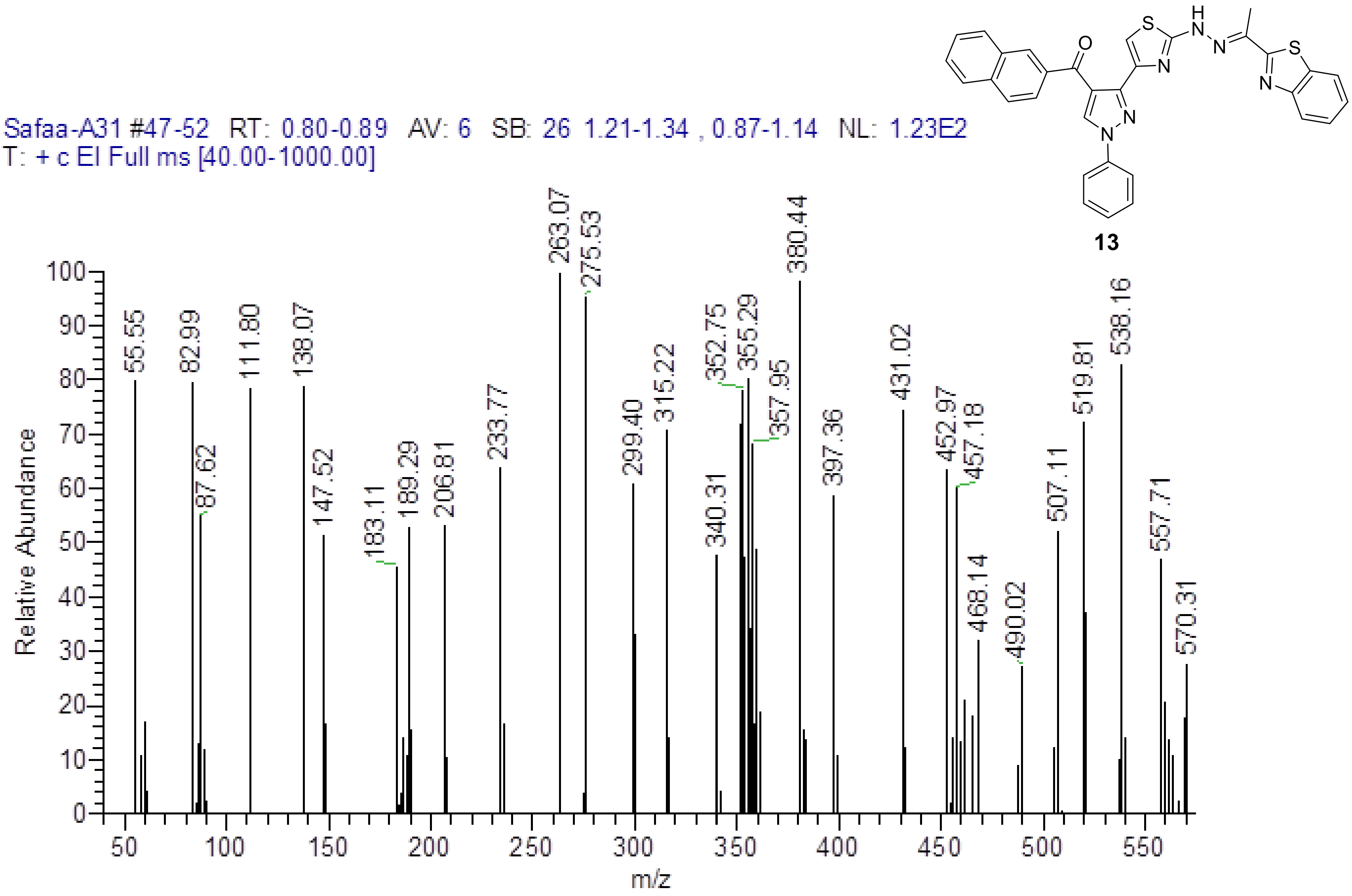

Figure S32: Mass spectrum of compound 13

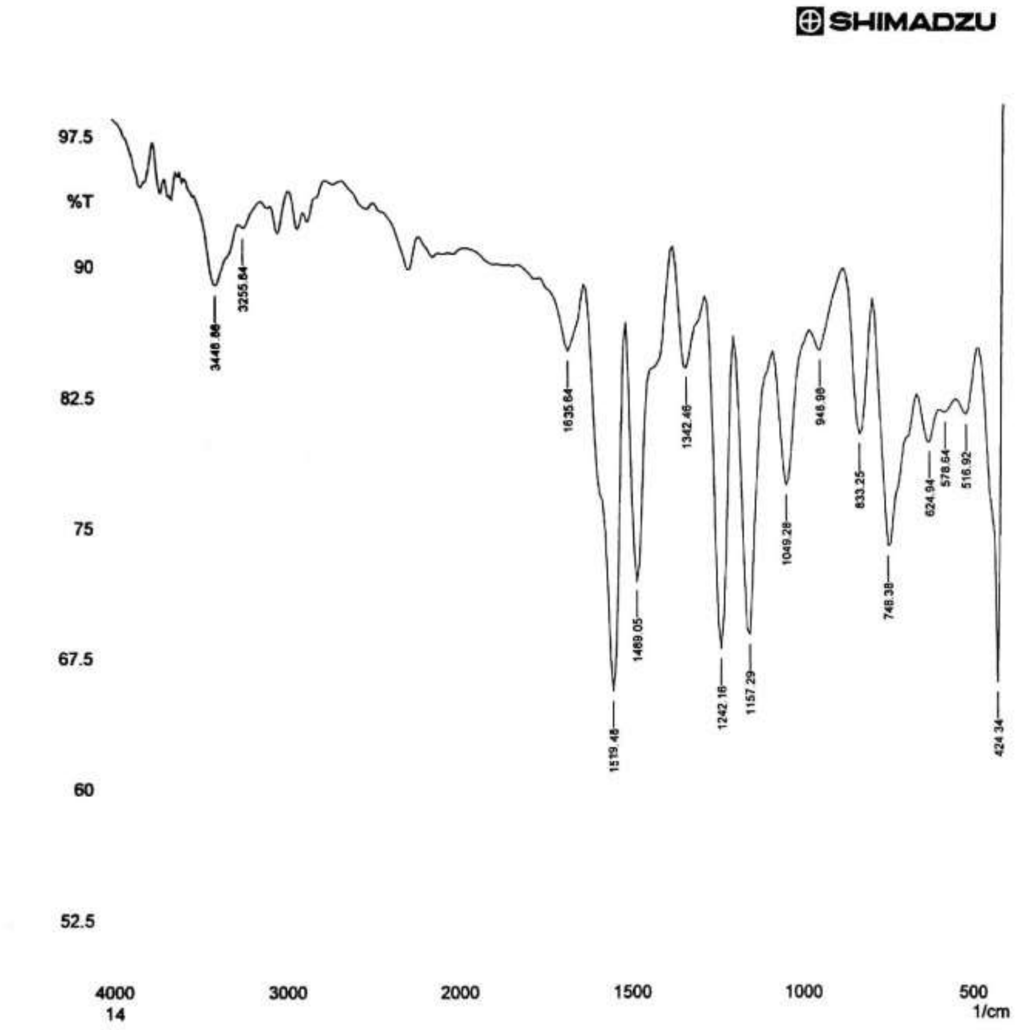

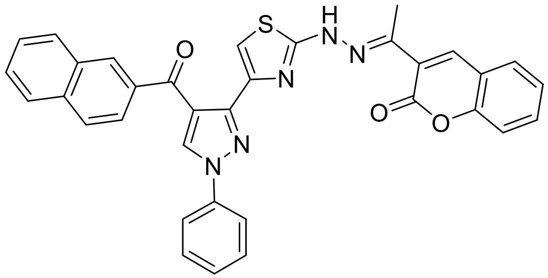

14

Figure S33: IR spectrum of compound 14

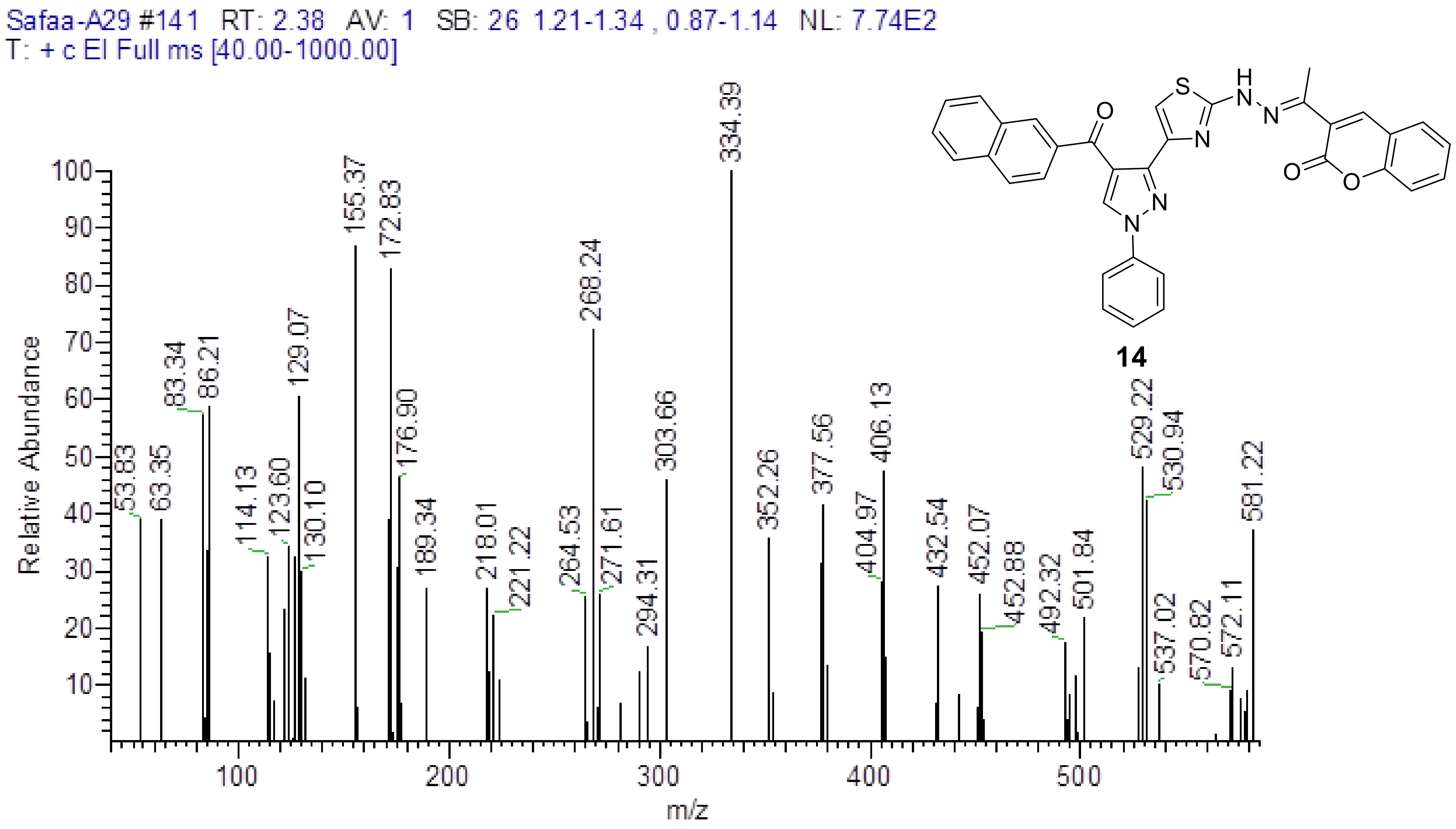

Figure S34: Mass spectrum of compound 14

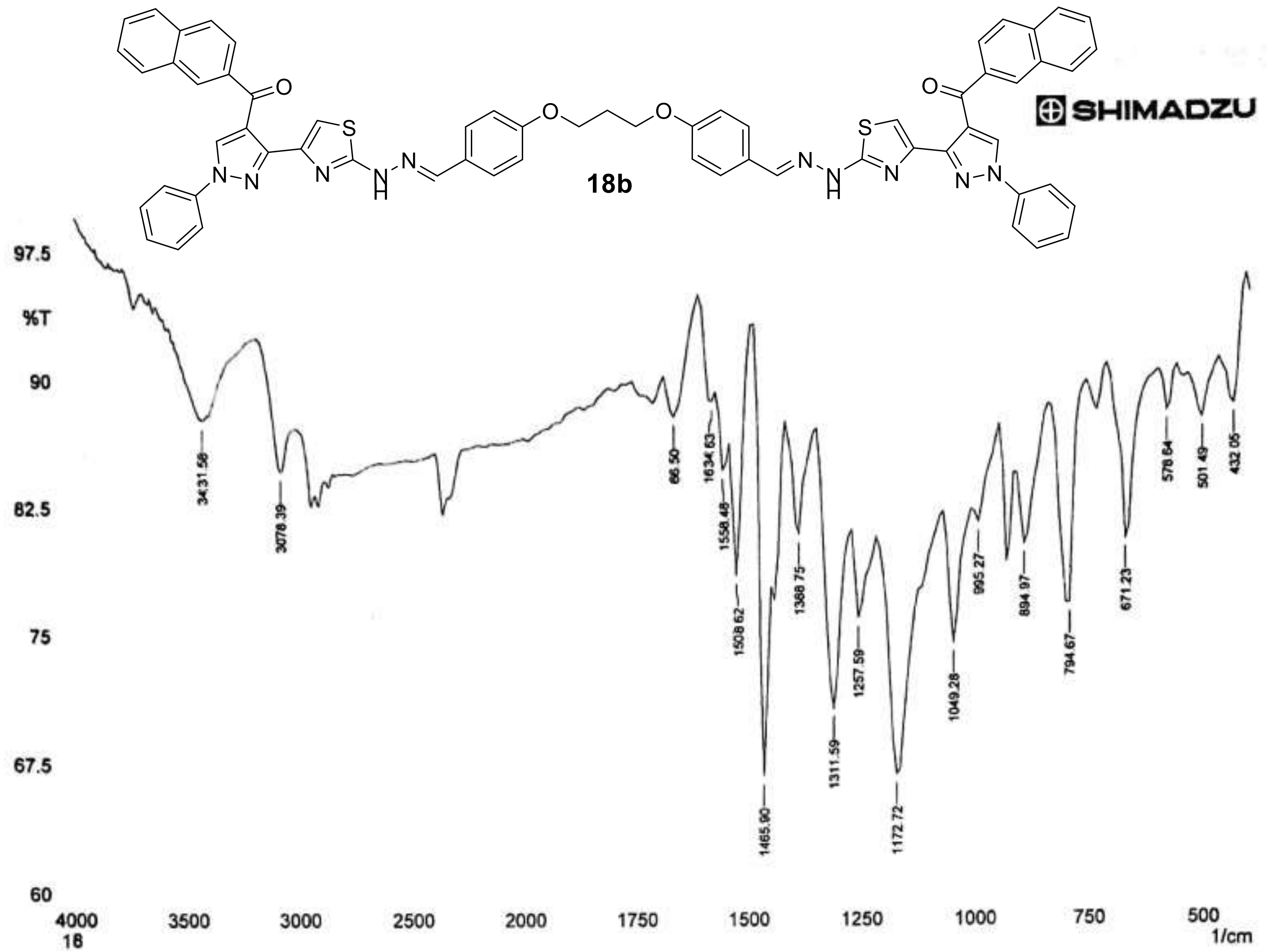

Figure S35: IR spectrum of compound 18b

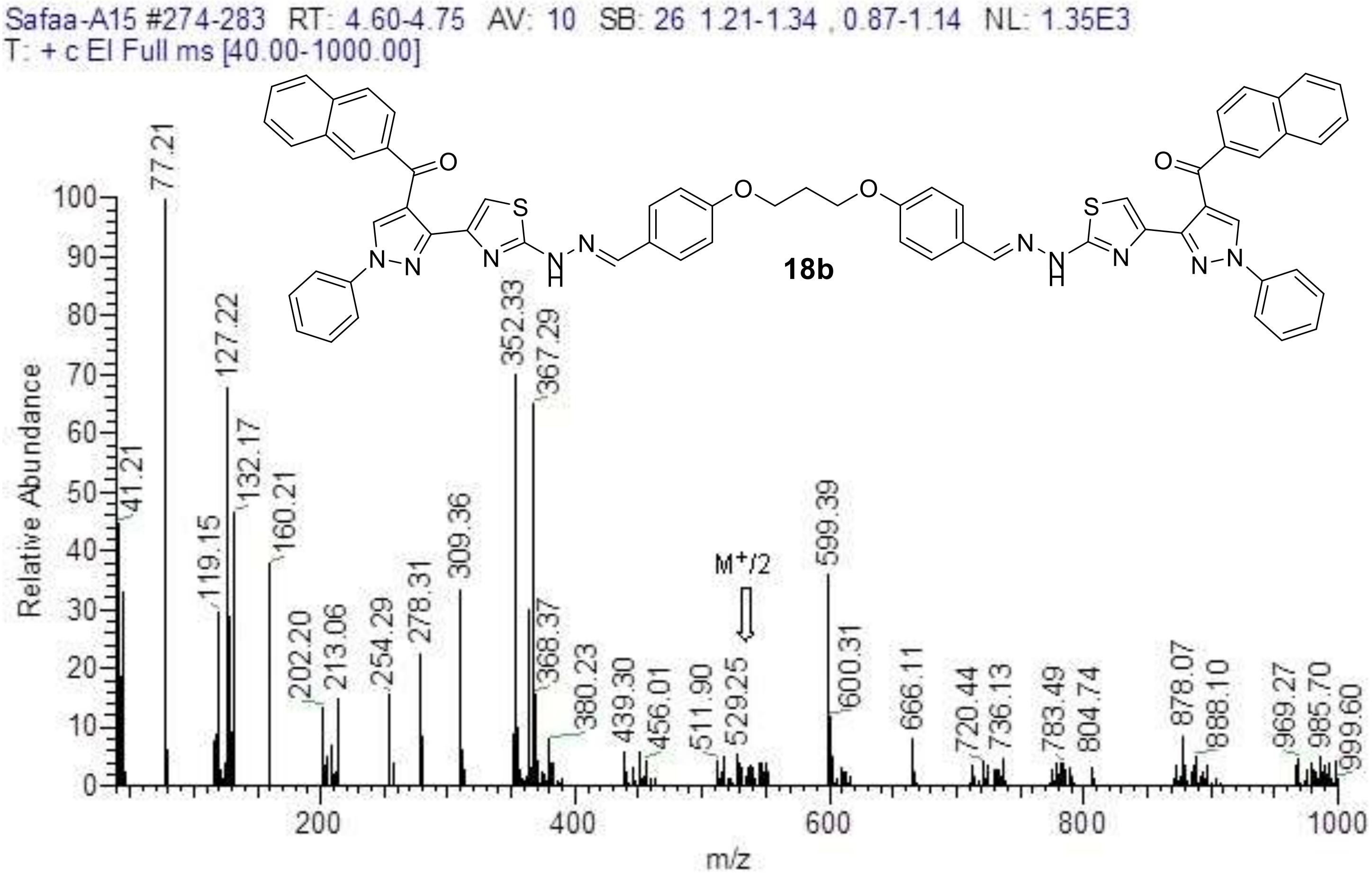

Figure S36: Mass spectrum of compound 18b

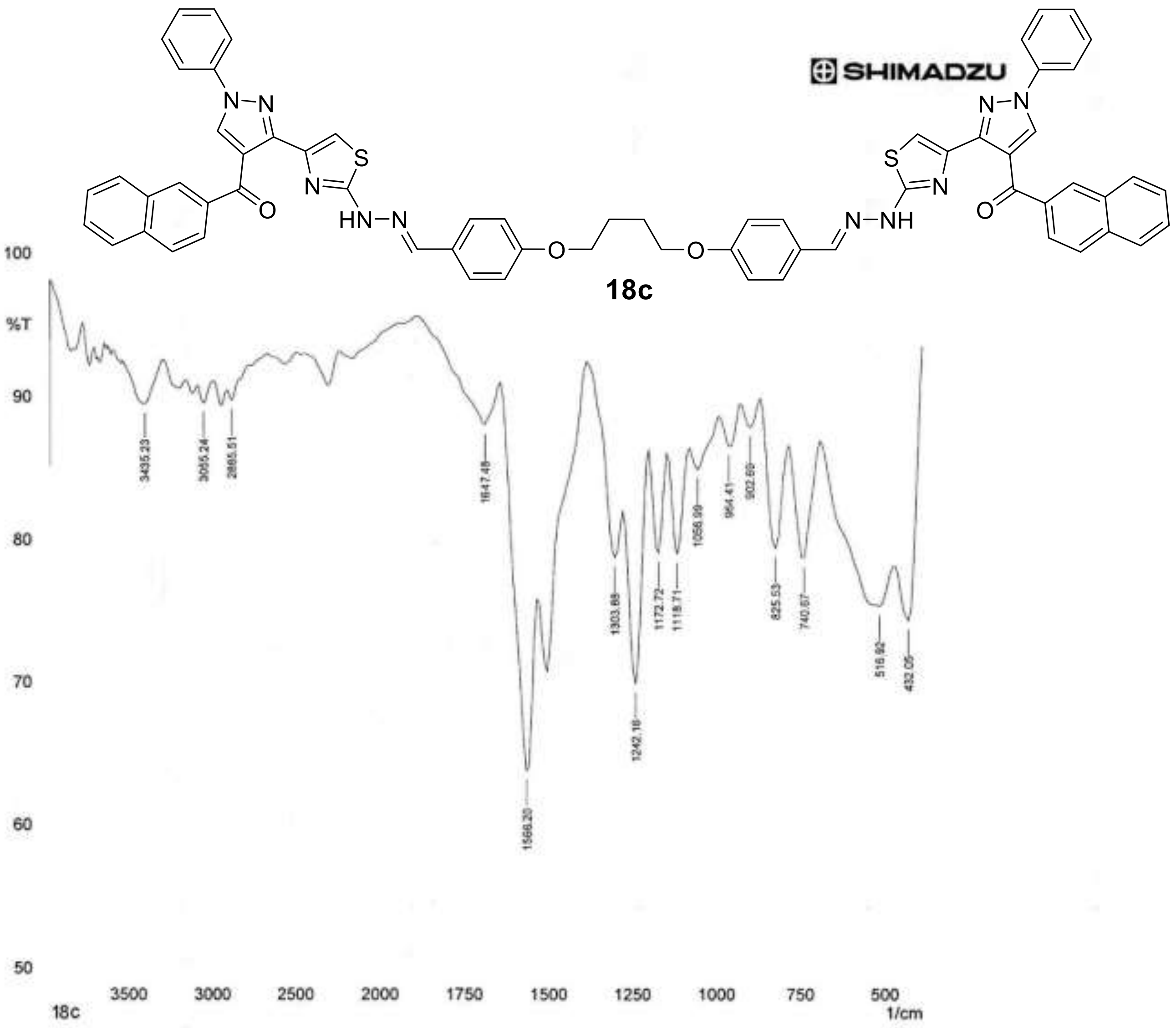

Figure S37: IR spectrum of compound 18c

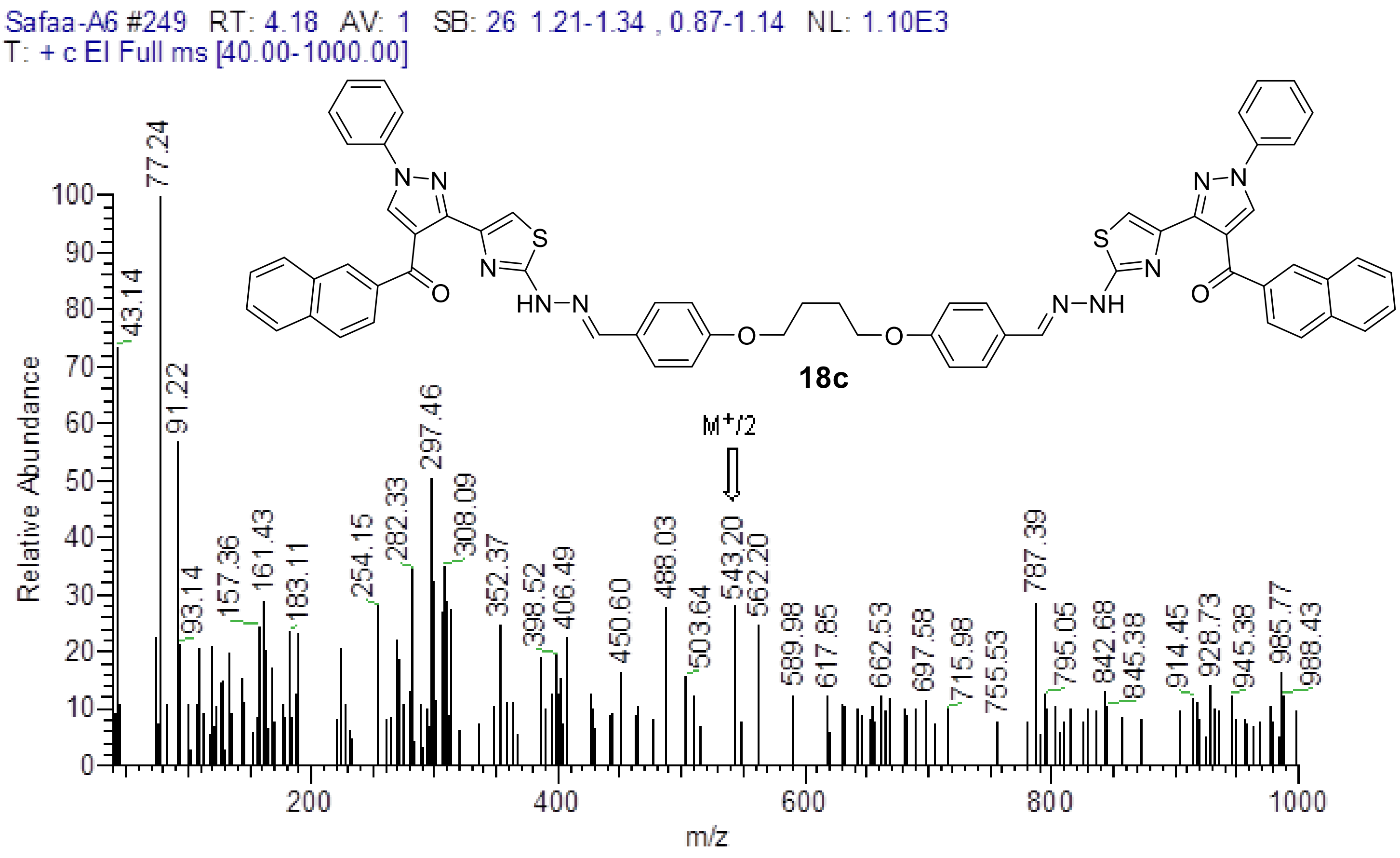

Figure S38: Mass spectrum of compound 18c

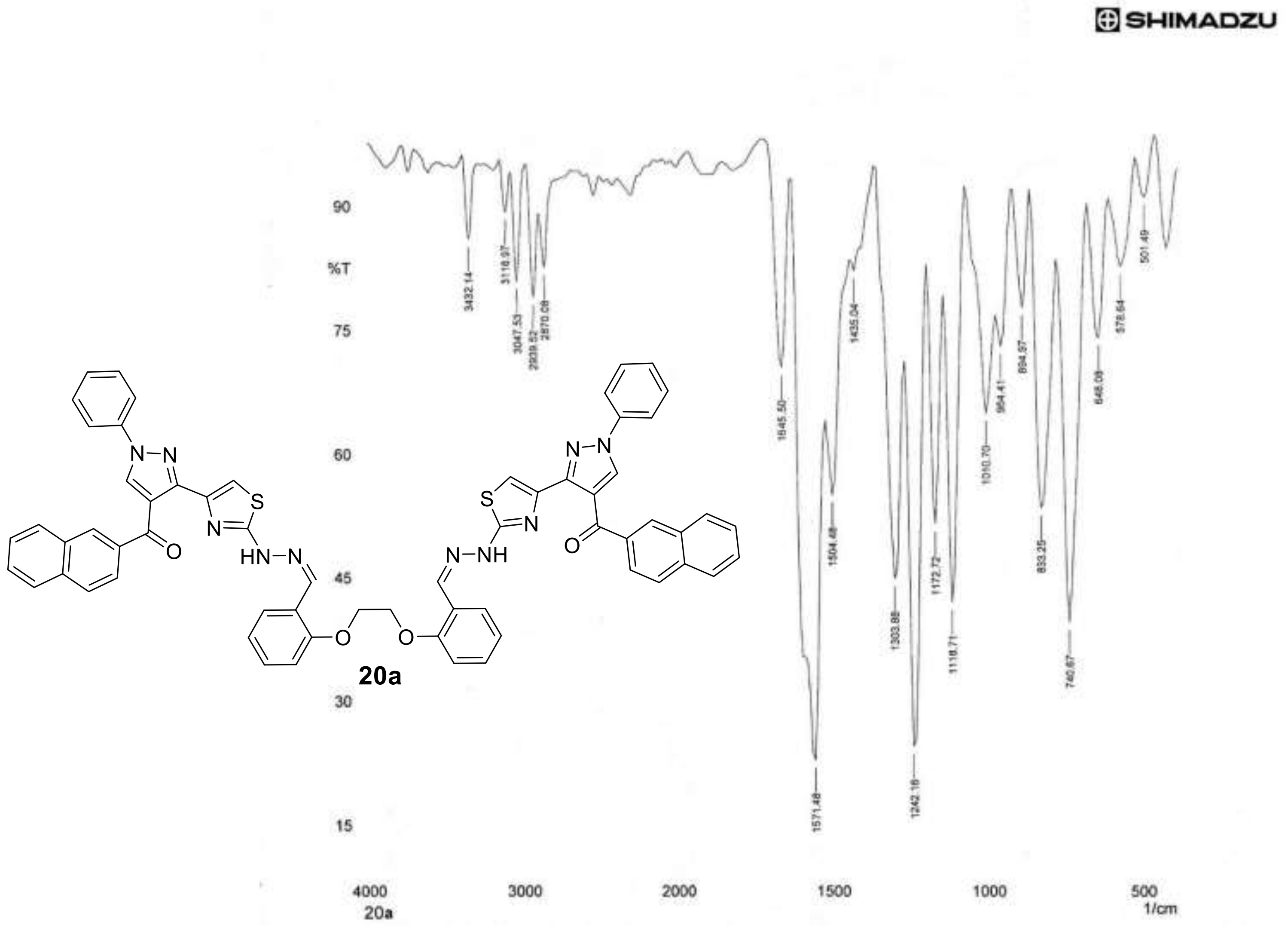

Figure S39: IR spectrum of compound 20a

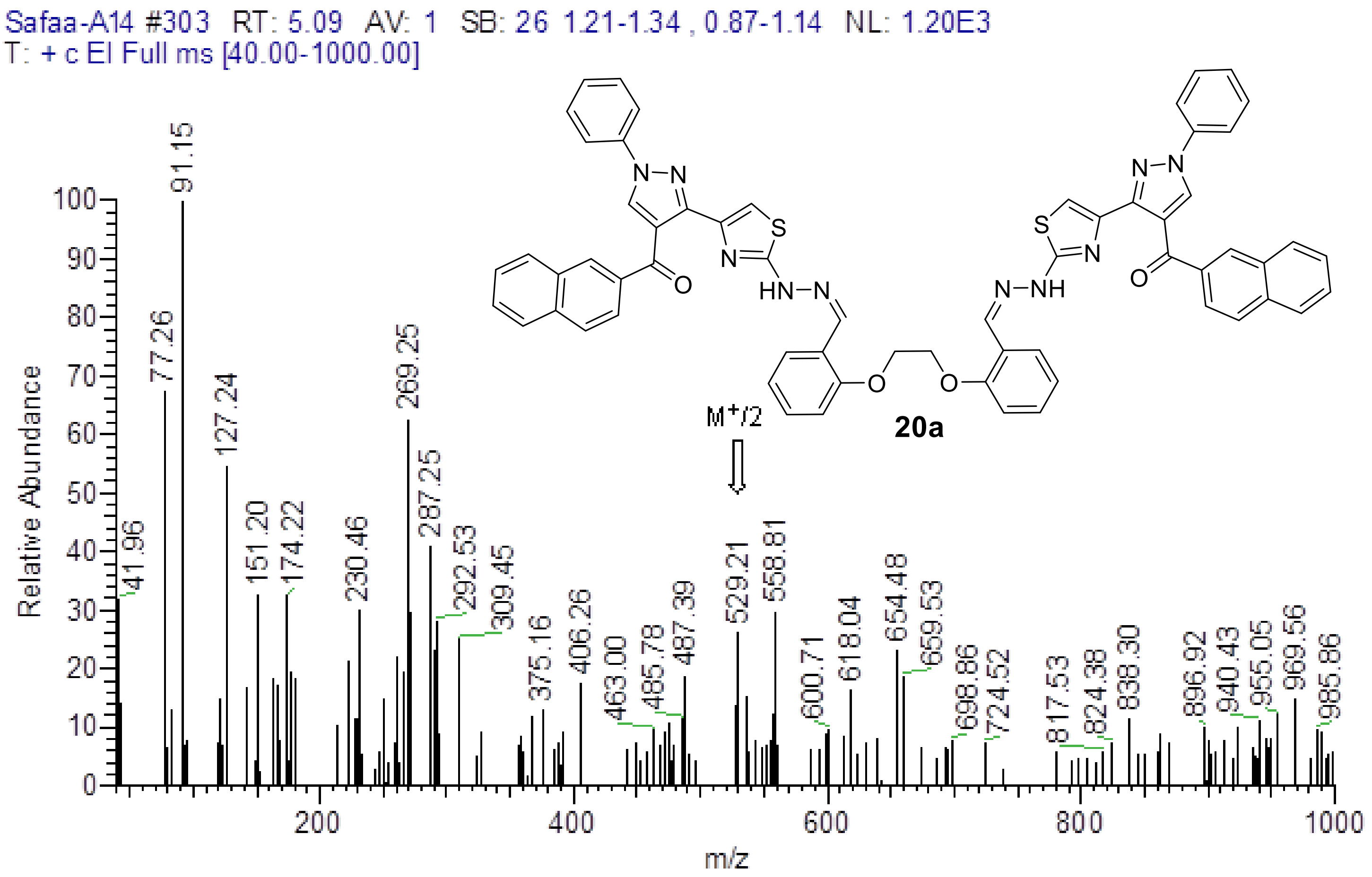

Figure S40: Mass spectrum of compound 20a

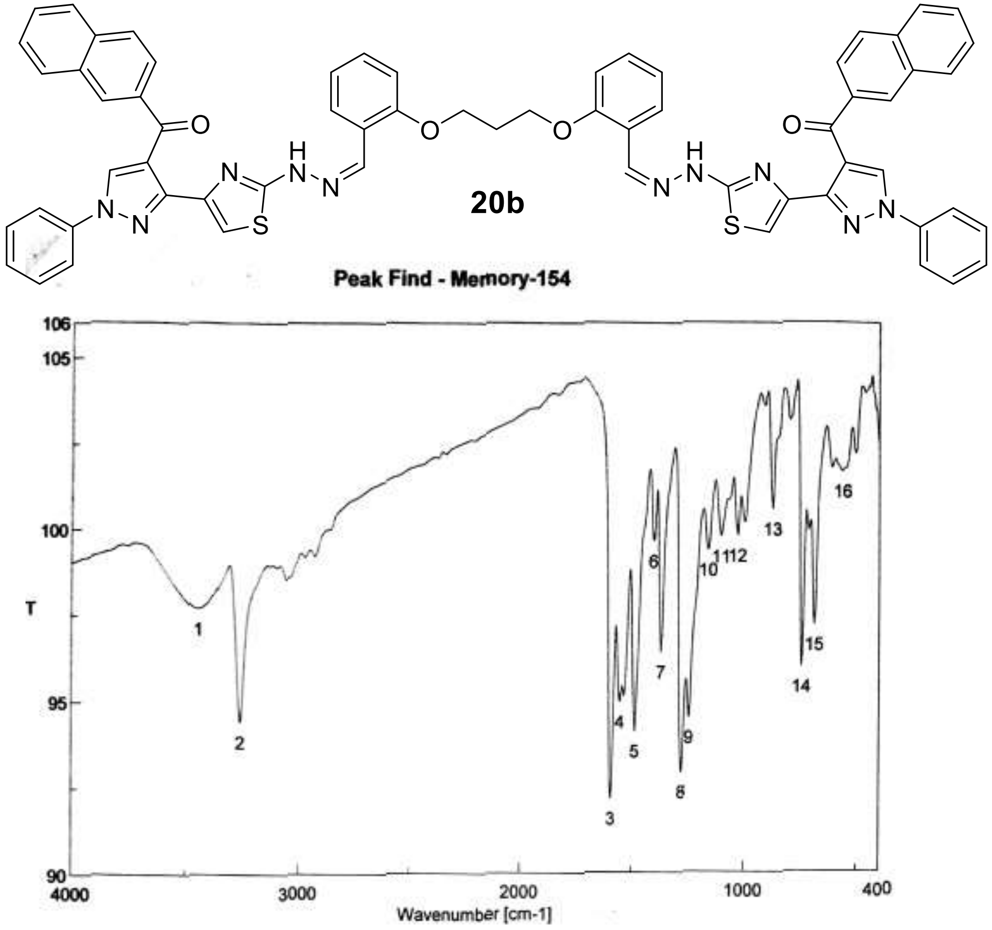

[Result of Peak Picking]

No.	Position	Intensity	No.	Position	Intensity	No.	Position	Intensity
1	3429.39	97.7059	2	3258.14	94.4471	3	1645.98	92.1638
4	1573.31	94.9862	5	1488.78	94.1302	6	1408.75	99.6424
7	1374.03	96.4379	8	1280.5	92.9448	9	1247.72	94.5079
10	1166.72	99.3682	11	1109.83	99.7488	12	1035.59	99.7711
13	880.345	100.541	14	746.317	95.9655	15	690.391	97.1811
16	565.041	101.63						

Figure S41: IR spectrum of compound 20b

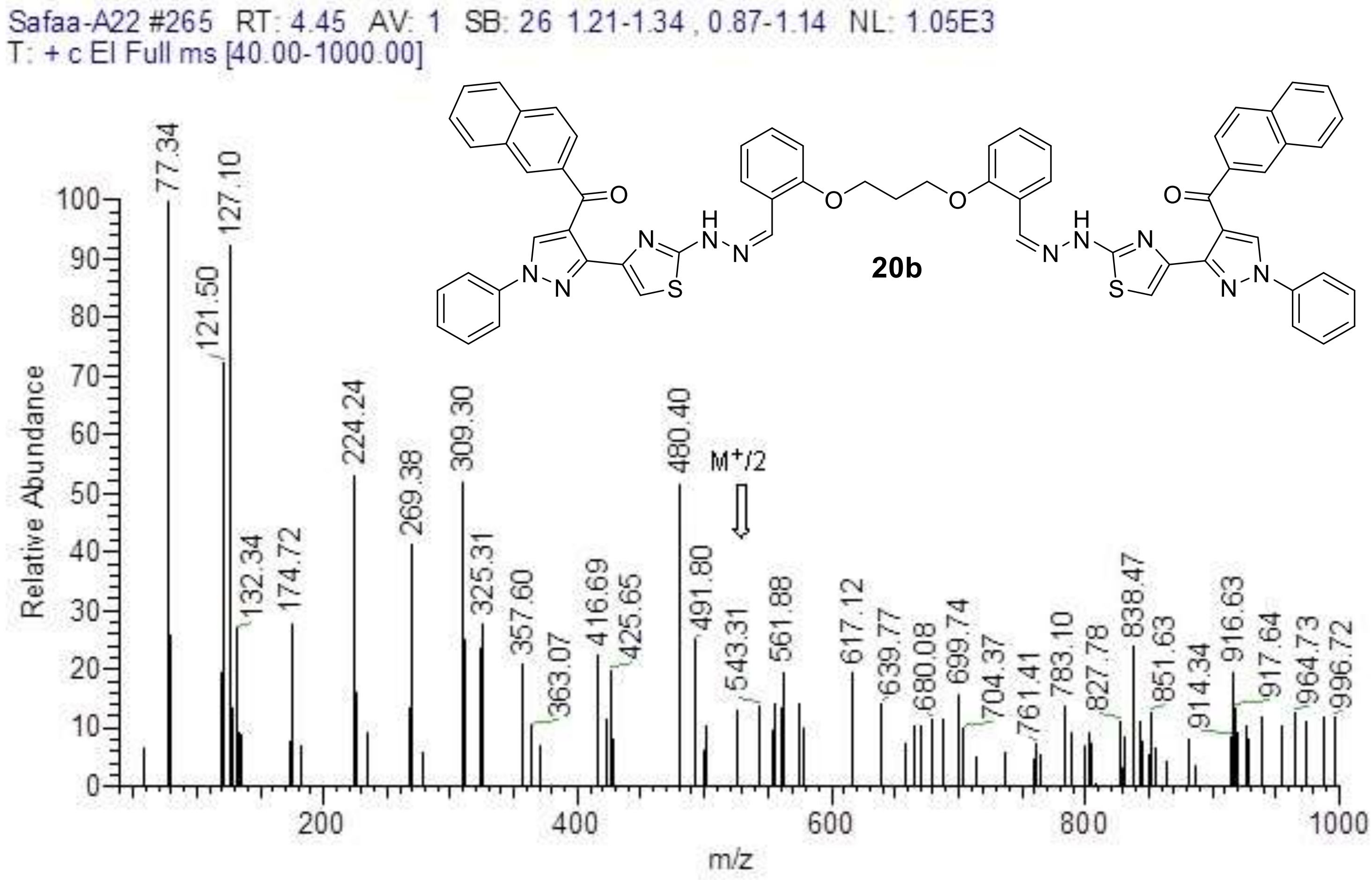

Figure S42: Mass spectrum of compound 20b

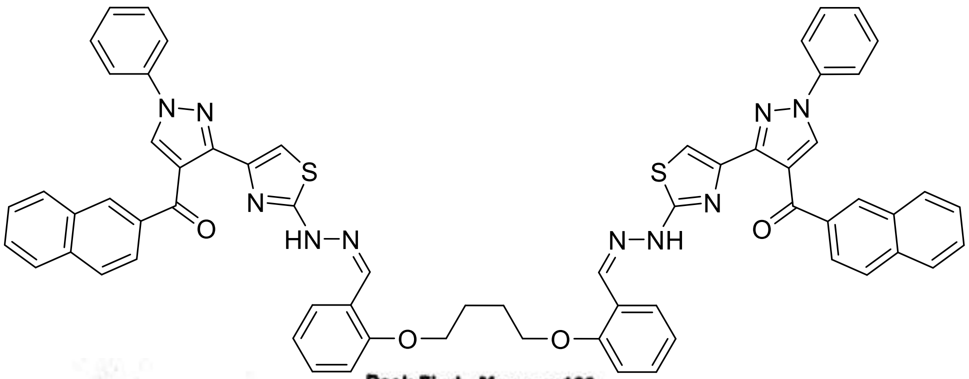

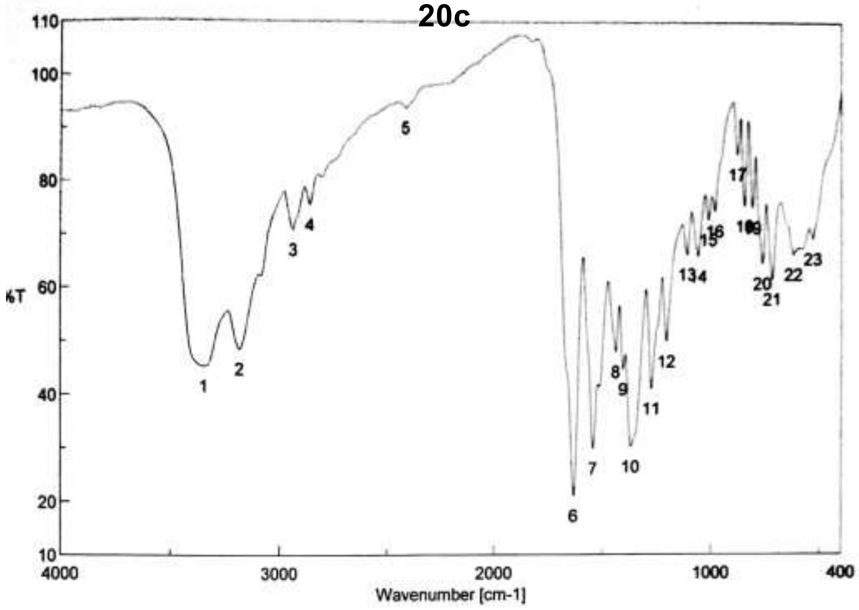

[Result of Peak Picking]

No.	Position	Intensity	No.	Position	Intensity	No.	Position	Intensity
1	3439.85	45.4684	2	3183.9	48.6791	3	2937.06	70.9913
4	2859.92	75.6664	5	2419.26	93.7391	6	1601.41	21.0141
7	1507.67	29.9446	8	1443.46	48.0871	9	1408.75	44.8135
10	1373.07	30.2678	11	1278.57	41.1486	12	1210.11	49.9665
13	1117.55	66.1928	14	1067.41	65.8193	15	1020.16	72.6643
16	990.268	74.2219	17	887.095	84.8298	18	853.347	75.1492
19	816.706	74.8375	20	768.494	64.4113	21	722.211	61.4776
22	622.895	66.0879	23	533.221	69.0038			

Figure S43: IR spectrum of compound 20c

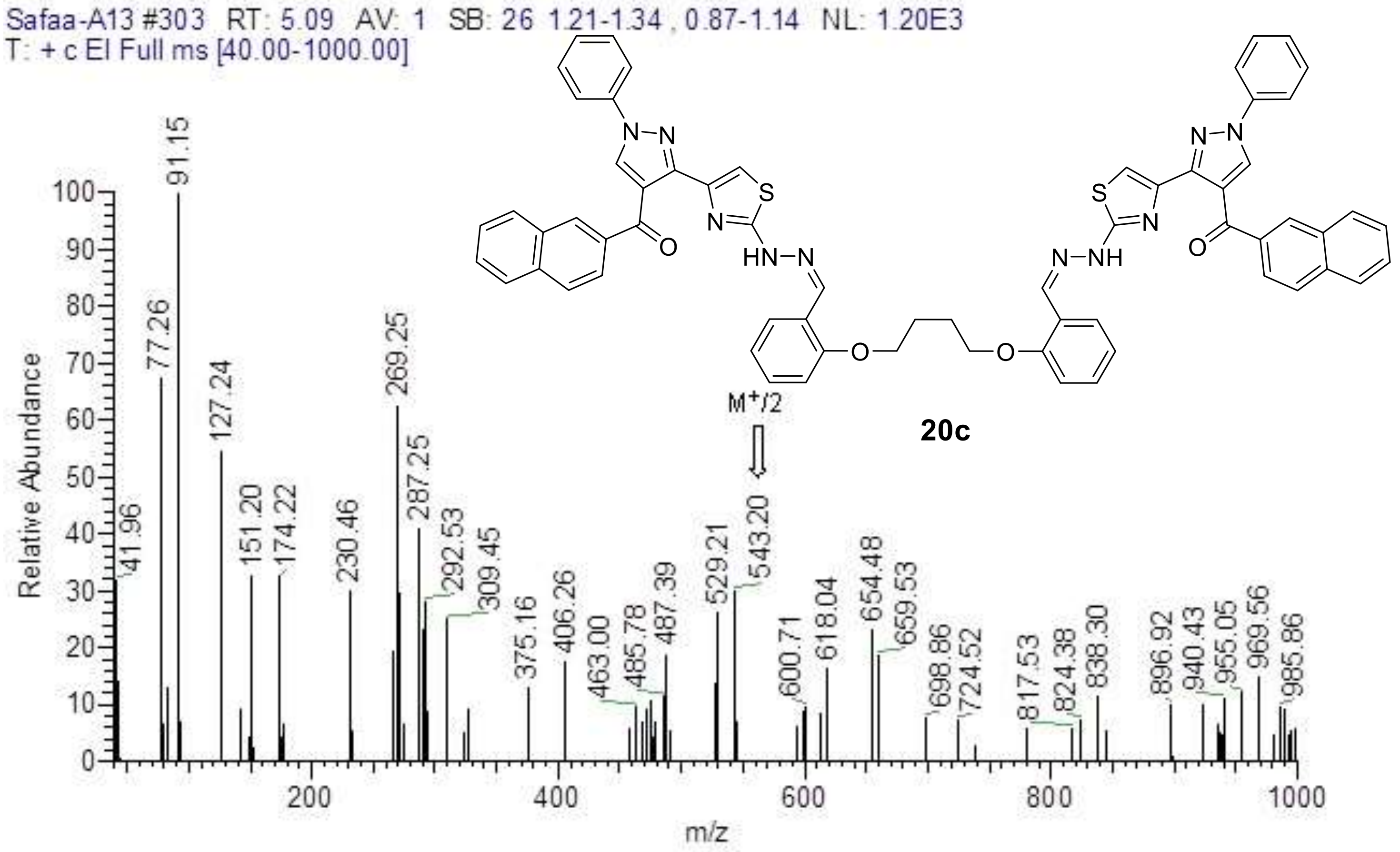

Figure S44: Mass spectrum of compound 20c
